# Integrated Multiomic Analysis Reveals an EBV‐Associated MIF–SPP1^+^ Macrophage Axis Linking Tumor Stemness and Immune Dysfunction in Nasopharyngeal Carcinoma

**DOI:** 10.1002/advs.78450

**Published:** 2026-10-31

**Authors:** Guo‐Ying Liu, Shu‐Hui Lv, Shui‐Qing He, Yao‐Fei Jiang, Xing‐Si Peng, Jian‐Cong Sun, Fen He, Hai‐Yan Qin, Nian Lu, Wei‐Xin Bei, Lin‐Yu Zhao, Wan‐Qin Shen, Ze‐Yu Zhao, Lin Wang, Yan‐Qun Xiang, Wang‐Zhong Li

**Affiliations:** ^1^ Department of Nasopharyngeal Carcinoma Sun Yat‐sen University Cancer Center the State Key Laboratory of Oncology in South China Collaborative Innovation Center for Cancer Medicine Guangdong Key Laboratory of Nasopharyngeal Carcinoma Diagnosis and Therapy Guangzhou China; ^2^ Guangdong Provincial Key Laboratory of Malignant Tumor Epigenetics and Gene Regulation Department of Radiation Oncology Medical Research Center Sun Yat‐Sen Memorial Hospital Sun Yat‐Sen University Guangzhou China; ^3^ Department of Oncology The First Affiliated Hospital of Nanchang University Nanchang 330006 China; ^4^ Department of Radiation Oncology The First Affiliated Hospital of Guangzhou Medical University State Key Laboratory of Respiratory Disease National Clinical Research Center for Respiratory Disease National Center for Respiratory Medicine Guangzhou Institute of Respiratory Health Guangzhou China; ^5^ Department of Medical Imaging Sun Yat‐sen University Cancer Center Guangzhou China

**Keywords:** Epstein–Barr virus, immunosuppressive, Nasopharyngeal carcinoma, SPP1, stemness, tumor microenvironment, tumor‐associated macrophages

## Abstract

Elucidating how Epstein–Barr virus (EBV) shapes tumor–immune interactions is important for improving therapy in nasopharyngeal carcinoma (NPC). This multi‐cohort analysis integrates single‐cell, spatial, and bulk transcriptomic data with multiplex immunofluorescence and functional assays. Single‐cell profiling identifies stemness‐associated epithelial states and enrichment of SPP1^+^ tumor‐associated macrophages (TAMs) in EBV^+^ NPC. SPP1^+^ TAM abundance is associated with stemness‐associated epithelial features and dysfunctional T‐cell states, while high MIF and SPP1^+^ macrophage signatures are associated with shorter progression‐free survival. Within the EBV^+^ spatial cohort, SPP1^+^ TAMs co‐occur with stemness‐associated tumor cells, exhausted CD8^+^ T cells, and regulatory T cells in Niche 5, which is more frequent in non‐response tumors following chemoimmunotherapy. Functional experiments show increased MIF expression and secretion by EBV^+^ epithelial cells. MIF inhibition attenuates conditioned‐medium‐induced monocyte migration and SPP1‐associated TAM‐like programming, whereas recombinant MIF reproduces these effects. Conversely, macrophage‐derived SPP1 increases stemness‐associated marker expression and tumorsphere formation in EBV^+^ epithelial and tumor models. It also impairs primary CD8^+^ T‐cell proliferation, effector function, and cytotoxic activity while increasing exhaustion‐associated markers; CD44 blockade partially reverses these effects. Collectively, these findings support an EBV‐associated MIF–SPP1 macrophage–tumor/T‐cell crosstalk model linking stemness‐associated epithelial phenotypes with immune dysfunction and adverse clinical outcomes.

## Graphical abstract

This study maps how Epstein–Barr virus reshapes the cellular ecosystem of nasopharyngeal carcinoma. It identifies SPP1^+^ macrophages as partners of stemness‐associated tumor cells and dysfunctional T cells, links tumor‐derived MIF to macrophage recruitment and programming, and shows that macrophage‐derived SPP1 promotes tumorsphere formation and impairs CD8^+^ T‐cell function partly through CD44.

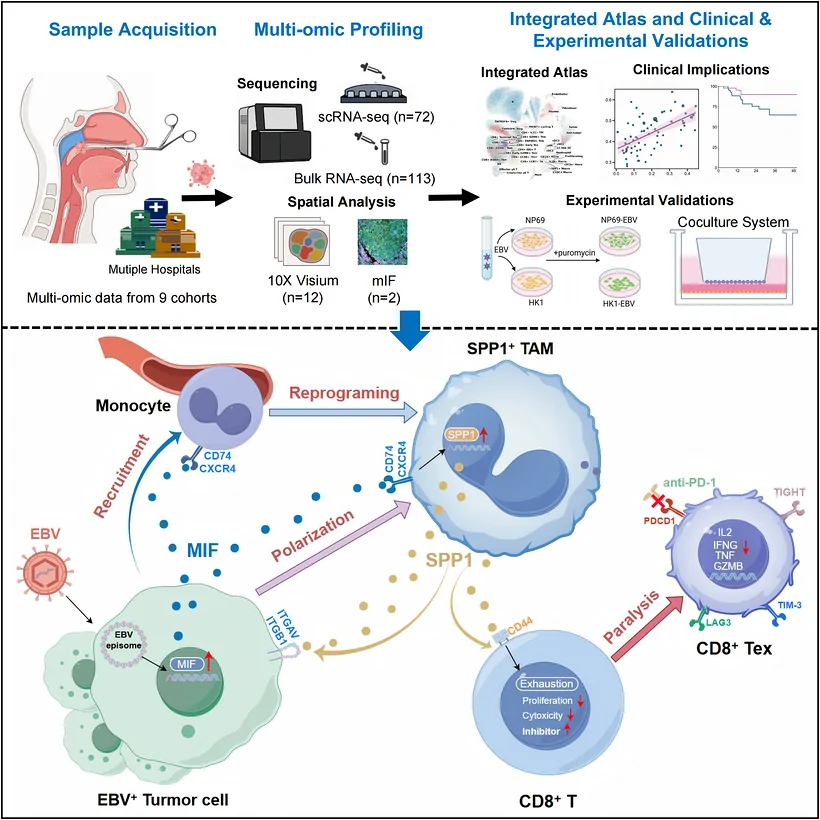

## 1 Introduction

Nasopharyngeal carcinoma (NPC) is an epithelial malignancy of the nasopharynx, marked by aggressive behavior, early metastatic dissemination, and a high propensity for distant spread [1]. Over 75% of patients present with locoregionally advanced disease at diagnosis, for which concurrent chemoradiotherapy remains the standard of care [2]. While recent clinical trials—including those conducted by our group—have achieved locoregional control rates approaching 90% in this setting, distant metastasis still occurs in approximately 20% of cases following treatment [3, 4, 5, 6, 7]. Our recent retrospective and prospective studies further demonstrate that metastatic NPC responds poorly to standard therapies, with a median progression‐free survival of less than one year—highlighting a major obstacle to improving long‐term outcomes [8, 9, 10]. These challenges underscore an urgent need to elucidate the molecular and cellular mechanisms that drive NPC progression. A deeper understanding of these mechanisms is critical for identifying actionable targets and developing more effective therapeutic strategies [11]. This includes a critical examination of the malignant features of NPC cells and the dynamic interactions within the tumor microenvironment that facilitate disease progression.

Epstein‐Barr virus (EBV) infection is the predominant etiological driver of NPC, with EBV positivity observed in up to 95% of patients in endemic regions [12]. A defining feature of EBV‐associated NPC is its highly inflamed tumor microenvironment (TME), characterized by dense infiltration of immune cells such as lymphocytes, macrophages, and mast cells [13]. Among these, tumor‐associated macrophages (TAMs) comprise a key immunoregulatory population, playing pivotal roles in tumor progression, immune evasion, and therapeutic resistance [14]. Through the secretion of diverse cytokines and growth factors, TAMs facilitate tumor cell invasion, promote angiogenesis, and suppress anti‐tumor immunity. In addition, TAMs serve as key drivers of cancer‐related inflammation, sustaining tumor cell stemness and contributing to disease recurrence and refractoriness [15, 16, 17]. In this context, elucidating the complex interplay between EBV infection, malignant cells, and TAMs is critical to understanding the underlying mechanisms that drive NPC progression [13, 18]. Our previous clinical studies, along with those of others, have demonstrated a robust link between TAM phenotypes and the risk of recurrence and metastasis, underscoring their functional importance in driving NPC progression [19, 20]. Using single‐cell RNA sequencing (scRNA‐seq) analysis, we further demonstrated that EBV‐associated NPCs with high plasma EBV DNA levels exhibit a distinct immunosuppressive tumor TME enriched in M2‐like TAMs and activated myeloid innate immunity programs—including pathways involved in angiogenesis, cell cycle regulation, and cytokine signaling—suggesting a role for EBV infection in shaping a pro‐tumor immune landscape [21].

However, prior studies, including our own, have been limited by small sample sizes, insufficient statistical power, and the lack of stratification by EBV infection status, underscoring the need for further work to delineate the contribution of EBV to TME evolution during malignant progression [21, 22, 23, 24, 25, 26, 27, 28]. To address this gap, we conducted a comprehensive integrative analysis leveraging scRNA‐seq, spatial transcriptomics (ST), bulk RNA‐seq, and multiplex immunofluorescence (mIF) across multiple NPC cohorts. By systematically characterizing the heterogeneity of malignant epithelial and immune cells, and mapping intercellular communication networks and spatial niches under distinct EBV infection statuses, we reveal an EBV‐associated SPP1^+^ macrophage–tumor crosstalk that is associated with enhanced tumor stemness and immunosuppressive features in NPC.

## 2 Results

### 2.1 scRNA‐seq Reveals Distinct Tumor Microenvironment Between EBV^+^ and EBV^−^ NPCs

The overall study design and computational workflow of multi‐omics analysis are illustrated in Figure 1a and Figure S1, respectively. A total of 72 biopsy specimens from six scRNA‐seq cohorts, including 15 non‐tumor samples, 7 EBV^−^ NPC samples, and 50 EBV^+^ NPC samples, were included in the scRNA‐seq analysis (Table S1). EBV^−^ and EBV^+^ NPC samples were distributed across three and five independent cohorts, respectively. Following stringent quality control (Figure S2) and batch correction with Harmony, selected for its superior integration performance (Figure S3), we constructed a large‐scale single‐cell atlas comprising 560,231 cells. Using canonical markers curated from the literature and top‐ranked genes (Figure 1b and Figure S4), we annotated cells into eight major types (Figure 1c). Sample‐level cell yields and cellular composition, together with EBV status, sex, and clinical stage, are summarized in Figure 1d. Although the major cell populations were broadly represented across samples, their proportions varied across disease stages and between EBV^−^ and EBV^+^ NPC. Stratified analyses revealed T/NK‐cell enrichment and B‐cell depletion in tumors, with higher epithelial fractions in early‐stage tumors and greater myeloid representation in EBV‐positive tumors (Figure 1e,f). To quantify EBV‐associated transcriptional divergence, we calculated Euclidean distances between the average gene‐expression profiles of each major cell type in the EBV^−^ and EBV^+^ groups (Figure 1g). Epithelial cells exhibited the greatest transcriptional divergence, whereas myeloid cells showed the largest difference among the immune cell populations

**FIGURE 1:**
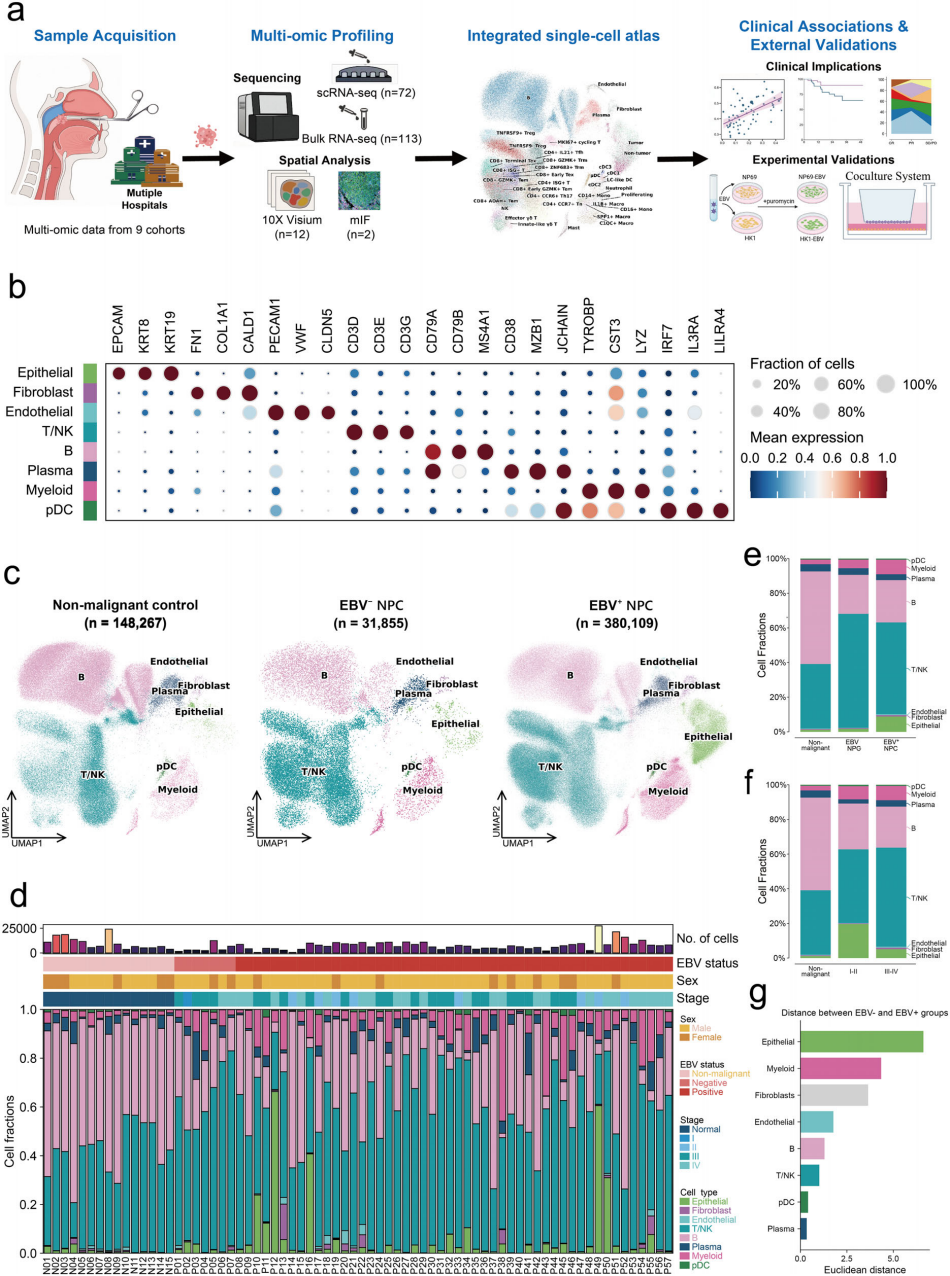
Construction of an integrated single‐cell atlas of EBV^−^ and EBV^+^ NPC. (a) Overview of the study design. (b) Dot plot of canonical markers for the eight major cell types. (c) UMAP visualization of the major cell types stratified by EBV status. (d) Sample‐level cell numbers and cellular compositions, with annotations of EBV status, sex, and clinical stage. (e) Cellular compositions across non‐malignant controls, EBV^−^ and EBV^+^ NPC. (f) Cellular compositions across non‐malignant controls and NPC clinical stages. (g) Euclidean distances between the average gene‐expression profiles of the major cell types in EBV^−^ and EBV^+^ NPC. Abbreviations: EBV, Epstein–Barr virus; mIC, multiplex immunofluorescence; NPC, nasopharyngeal carcinoma; pDC, plasmacytoid dendritic cell; scRNA‐seq, single‐cell RNA sequencing; T/NK, T and natural killer cells; UMAP, uniform manifold approximation and projection.

### 2.2 EBV^+^ NPC Exhibit Stemness‐Associated and Developmental‐Plasticity Features

We first inferred CNVs from the transcriptomic profiles of epithelial cells and calculated CNV scores for each cell (Figure S5a). To distinguish malignant from normal epithelial cells, we performed Leiden clustering based on CNV signatures (Figure S5b). Clusters exhibiting elevated CNV scores and dominated by one or few samples rather than being broadly represented across multiple samples, were classified as tumor cells (Figure S5c–f and Figure 2a,b). Inferred CNV heatmaps revealed more extensive chromosomal gains and losses in tumor epithelial cells than in non‐tumor epithelial cells (Figure S5g). Both EBV^−^ and EBV^+^ tumor epithelial cells exhibited widespread but distinct CNV patterns (Figure 2c,d), highlighting potential EBV‐associated differences in chromosomal alteration profiles. Analysis of viral transcripts showed that EBV^+^ epithelial cells predominantly expressed the latency II‐associated genes LMP1 and LMP2B, together with the noncoding transcript EBER2. In contrast, latency III‐associated genes, including EBNA2, EBNA3A–C, and EBNA‐LP, and the immediate‐early lytic genes BZLF1 and BRLF1 were barely detected, indicating a predominantly latency II‐like program with minimal lytic activation (Figure 2e). Consistent with the predominantly latent EBV program in patient tumors, EBV‐infected NP69 and HK1 cells and C666‐1 cells expressed latency‐associated genes, whereas representative lytic‐cycle genes showed no significant induction (Figure S6).

**FIGURE 2:**
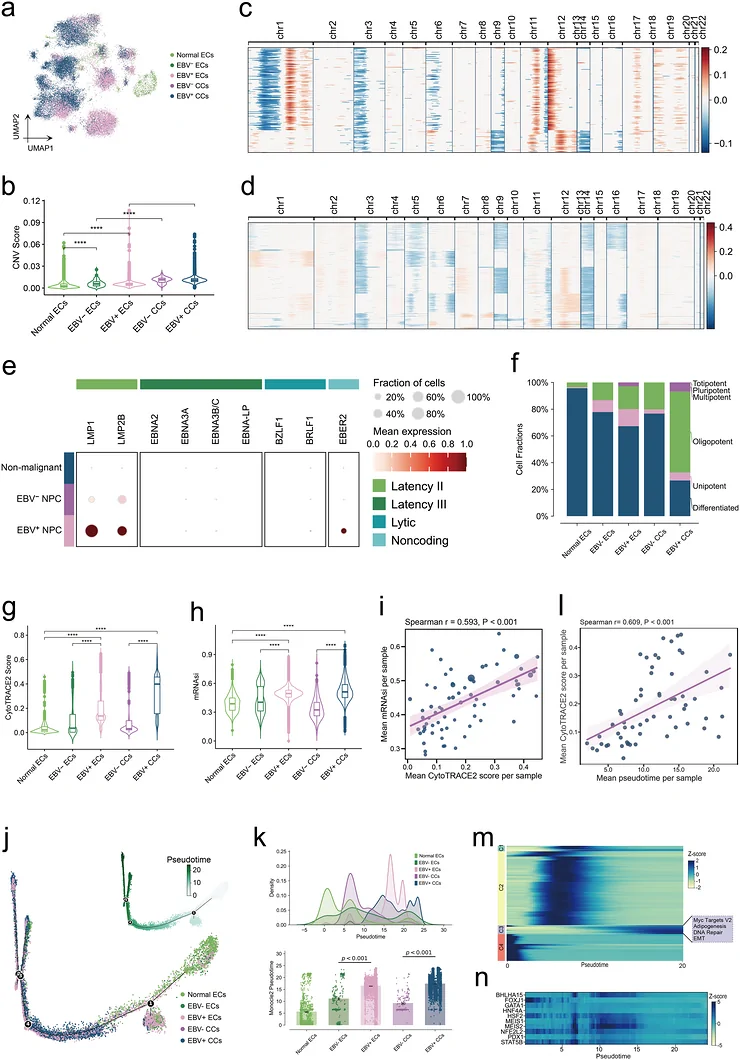
CNV‐based classification and stemness profiling of epithelial cells in NPC. (a) UMAP visualization of epithelial cells stratified by CNV‐defined cellular identity and EBV status. (b) Distribution of inferred CNV scores across epithelial‐cell groups. (c‐d) Inferred chromosomal CNV profiles of tumor epithelial cells from EBV^−^ (c) and EBV^+^ (d) NPC. Red and blue indicate inferred chromosomal gains and losses, respectively. (e) Dot plot showing EBV latency‐associated, lytic, and noncoding transcripts across epithelial‐cell from non‐malignant controls and EBV^−^ and EBV^+^ NPC. (f) Distribution of CytoTRACE2‐inferred cellular potency states. (g) CytoTRACE2 scores and (h) OCLR‐derived mRNAsi across epithelial‐cell groups. (i) Sample‐level correlation between mean CytoTRACE2 and mRNAsi scores. (j) Monocle 2‐based epithelial trajectory colored by cellular group or inferred pseudotime. (k) Distribution of epithelial‐cell groups along pseudotime. (l) Sample‐level correlation between mean CytoTRACE2 score and Monocle 2‐based pseudotime. (m) Heatmap of pseudotime‐associated gene expression modules and their Hallmark annotations. (n) Dynamic transcription factor activities along pseudotime. Normal ECs denote epithelial cells from non‐malignant controls; EBV^−^ and EBV^+^ ECs denote CNV‐defined non‐tumor epithelial cells from EBV^−^ and EBV^+^ NPC, respectively; EBV^−^ and EBV^+^ CCs denote CNV‐defined tumor epithelial cells from the corresponding groups. Abbreviations: CC, cancer cell; CNV, copy‐number variation; EBV, Epstein–Barr virus; EC, epithelial cell; mRNAsi, mRNA expression‐based stemness index; NPC, nasopharyngeal carcinoma; OCLR, one‐class logistic regression; UMAP, uniform manifold approximation and projection.

CytoTRACE2‐based potency inference revealed that EBV^+^ tumor epithelial cells were enriched for oligopotent and multipotent states, whereas EBV^−^ tumor cells were predominantly differentiated (Figure 2f). Consistently, both non‐tumor and tumor epithelial cells from EBV^+^ NPC exhibited higher CytoTRACE2 scores than their EBV^−^ counterparts (Figure 2g). OCLR‐derived mRNAsi showed a similar pattern: EBV^+^ tumor cells had higher mRNAsi than EBV^−^ tumor cells, while non‐tumor epithelial cells from EBV^+^ NPC also had higher scores than EBV^−^ and normal‐control epithelial cells (Figure 2h). At the sample level, mean CytoTRACE2 and mRNAsi scores were positively correlated (Spearman r = 0.593, *P* < 0.001; Figure 2i). Monocle 2 analysis reconstructed a branched epithelial trajectory (Figure 2j). EBV^+^ epithelial cells, particularly tumor cells, occupied later pseudotime positions, whereas normal‐control and EBV^−^ tumor epithelial cells were enriched at earlier positions (Figure 2k). Consistent with these findings, sample‐level mean CytoTRACE2 scores were positively correlated with mean Monocle 2 pseudotime (Spearman r = 0.609, *P* < 0.001; Figure 2l). Genes showing significant expression changes along pseudotime were clustered into four modules with distinct temporal patterns. Cluster 3, which was upregulated toward the trajectory terminus, was enriched for MYC targets V2, adipogenesis, DNA repair, and epithelial–mesenchymal transition (Figure 2m). Transcription‐factor activity analysis further revealed coordinated activation of MEIS1 and MEIS2 during intermediate‐to‐late pseudotime, implicating developmental and progenitor‐related regulatory programs (Figure 2n). Collectively, these analyses define an EBV‐associated epithelial state characterized by stemness‐associated and developmental‐plasticity features.

### 2.3 EBV^+^ NPC Exhibits Enhanced Stemness with Clinical Implications

Stemness‐associated genes, including *EPCAM*, *MYC*, *CCND2*, *ALDH1A1*, and *THY1*, showed higher expression in EBV^+^ than in EBV^−^ tumor epithelial cells (Figure 3a). CollecTRI analysis predicted increased activity of MYC and CUX1 in EBV^+^ tumor cells (Figure 3b). PROGENy further predicted higher activity of PI3K, estrogen, MAPK, and EGFR signaling, together with more modest increases in TNFα and NF‐κB pathway activity (Figure 3c). GSEA revealed enrichment of both a curated stemness signature and proliferation‐associated MYC targets V1/V2 and E2F targets in EBV^+^ tumor cells (Figure 3d), indicating that stemness‐associated and proliferative transcriptional features coexist in this population (Figure 3d). At the protein level, western blotting confirmed increased expression of SOX2, CD44, OCT‐4, and NANOG in EBV‐positive HK1 and NP69 derivatives relative to their parental counterparts, with similarly high expression observed in C666‐1 cells (Figure 3e,f).

**FIGURE 3:**
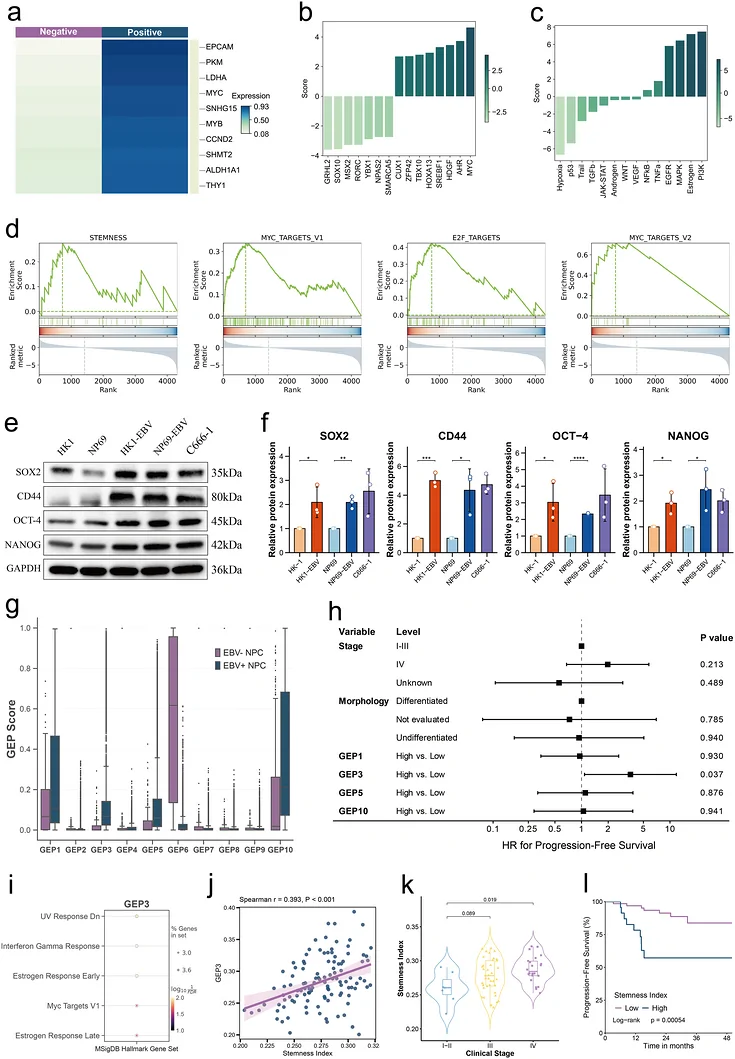
Stemness‐associated transcriptional programs and their clinical relevance in NPC. (a) Heatmap of representative stemness‐associated genes in EBV^−^ and EBV^+^ tumor epithelial cells. (b) Differential transcription‐factor activities inferred using CollecTRI. (c) Differential pathway activities inferred using PROGENy. Positive and negative scores indicate higher inferred activity in EBV^+^ and EBV^−^ tumor cells, respectively. (d) Gene‐set enrichment plots for the indicated stemness‐associated and Hallmark gene sets. (e) Representative western blots of SOX2, CD44, OCT‐4, and NANOG in parental, EBV‐infected, and C666‐1 cells. (f) Densitometric quantification of the corresponding protein bands from three independent experiments (n = 3). (g) Distribution of usage scores for identified GEPs in EBV^−^ and EBV^+^ malignant epithelial cells. (h) Forest plot of multivariable Cox regression evaluating the prognostic value of GEPs enriched in EBV^+^ NPC for progression‐free survival. Squares and horizontal lines represent hazard ratios and 95% confidence intervals, respectively. (i) Hallmark gene‐set annotation of GEP3. (j) Sample‐level correlation between mean GEP3 activity and the stemness index. (k) Distribution of the stemness index across clinical stages. (l) Kaplan–Meier analysis of progression‐free survival stratified by the stemness index. Data in (f) are presented as mean ± SD from three independent biological replicates; pairwise comparisons were performed using two‐sided unpaired Student's t‐tests. Abbreviations: CI, confidence interval; cNMF, consensus non‐negative matrix factorization; EBV, Epstein–Barr virus; FDR, false discovery rate; GEP, gene expression program; HR, hazard ratio; NPC, nasopharyngeal carcinoma.

To delineate recurrent transcriptional programs in malignant epithelial cells, consensus non‐negative matrix factorization (cNMF) was performed following component‐selection and spectrum‐filtering analyses, resolving ten gene expression programs (GEPs) (Figure S7a,b). The cellular distributions and dominant assignments of these programs revealed substantial intratumoral transcriptional heterogeneity (Figure S7c,d), while pairwise Euclidean distances quantified the similarity among GEPs. GEP1, GEP3, GEP5, and GEP10 showed higher usage scores in EBV^+^ tumor cells, whereas GEP6 was more prominent in EBV^−^ tumor cells (Figure 3g). Among the elevated programs in EBV^+^ NPC, high GEP3 usage was independently associated with shorter progression‐free survival (PFS) in multivariable Cox regression analysis (HR = 3.57, 95% CI = 1.08–11.8, *P* = 0.037; Figure 3h), consistent with the Kaplan–Meier analysis (log‐rank *P* = 0.02; Figure S7e). Hallmark annotation associated GEP3 with MYC targets V1, interferon‐γ response, and estrogen‐response signatures (Figure 3i). GEP3 usage also correlated positively with the stemness index across samples (Spearman r = 0.393, *P* < 0.001; Figure 3j). The stemness index was higher in stage IV than in stage III disease (P = 0.019; Figure 3k), and a high stemness index was associated with shorter PFS (log‐rank *P* = 0.0005; Figure 3l). Collectively, these analyses identify an EBV‐associated epithelial transcriptional state with higher potency and stemness scores, together with proliferative and developmental‐plasticity features.

### 2.4 SPP1^+^ TAMs Are Enriched in EBV^+^ NPC and Linked to Stemness and Poor Prognosis

Among immune compartments, myeloid cells showed the largest EBV‐associated transcriptional shifts; we therefore isolated this lineage for sub‐clustering. Canonical marker expression and top‐ranked genes resolved 12 myeloid subpopulations, including C1QC^+^, IL1B^+^, and SPP1^+^ macrophage subsets (Figure 4a,b and Figure S8a,b). Myeloid‐cell composition differed across non‐malignant controls, EBV^−^ NPC, and EBV^+^ NPC (Figure 4c). Observed‐to‐expected enrichment analysis revealed preferential representation of dendritic‐cell subsets in non‐malignant and EBV^−^ samples, whereas macrophage and monocyte populations were broadly enriched in EBV^+^ NPC (Figure 4d). Sample‐level quantification further demonstrated that the proportion of SPP1^+^ macrophages within the myeloid compartment was significantly higher in EBV^+^ NPC than in non‐malignant controls and EBV^−^ NPC (Figure 4e). Pathway enrichment analysis revealed distinct functional states among the three macrophage subsets. SPP1^+^ macrophages exhibited a mixed activation state characterized by prominent pro‐ and anti‐inflammatory programs, together with enhanced M2‐polarization, type I interferon‐response, hypoxia, and angiogenesis activities (Figure 4f). Consistently, quantitative PCR (qPCR) validation showed that *SPP1* overexpression generally increased, whereas *SPP1* knockdown decreased, the expression of genes associated with hypoxia, interferon responses, and angiogenesis in macrophages (Figure 4g). Across scRNA‐seq samples, the proportion of SPP1^+^ macrophages correlated positively with the mean epithelial CytoTRACE2 score (Spearman r = 0.412, *P* < 0.001; Figure 4h). Similarly, across bulk RNA‐seq samples, *SPP1* expression correlated positively with the stemness index (Spearman r = 0.363, *P* < 0.001; Figure 4i). Kaplan–Meier analyses showed no significant associations between PFS and the *C1QC* or *IL1B* signatures, including their corresponding macrophage signatures (all *P* > 0.05; Figure S9a–d). In contrast, high scores for the SPP1 and SPP1^+^ macrophage signatures were associated with shorter PFS (log‐rank *P* = 0.041 and 0.043, respectively; Figure S9e,f). In multivariable Cox regression adjusted for clinical stage and histological morphology, a high SPP1^+^ macrophage signature remained independently associated with shorter PFS (HR = 6.94, 95% CI = 1.72–27.9, *P* = 0.006; Figure 4j). Collectively, these findings identify SPP1^+^ TAMs as an EBV‐associated myeloid population linked to epithelial stemness and adverse clinical outcomes.

**FIGURE 4:**
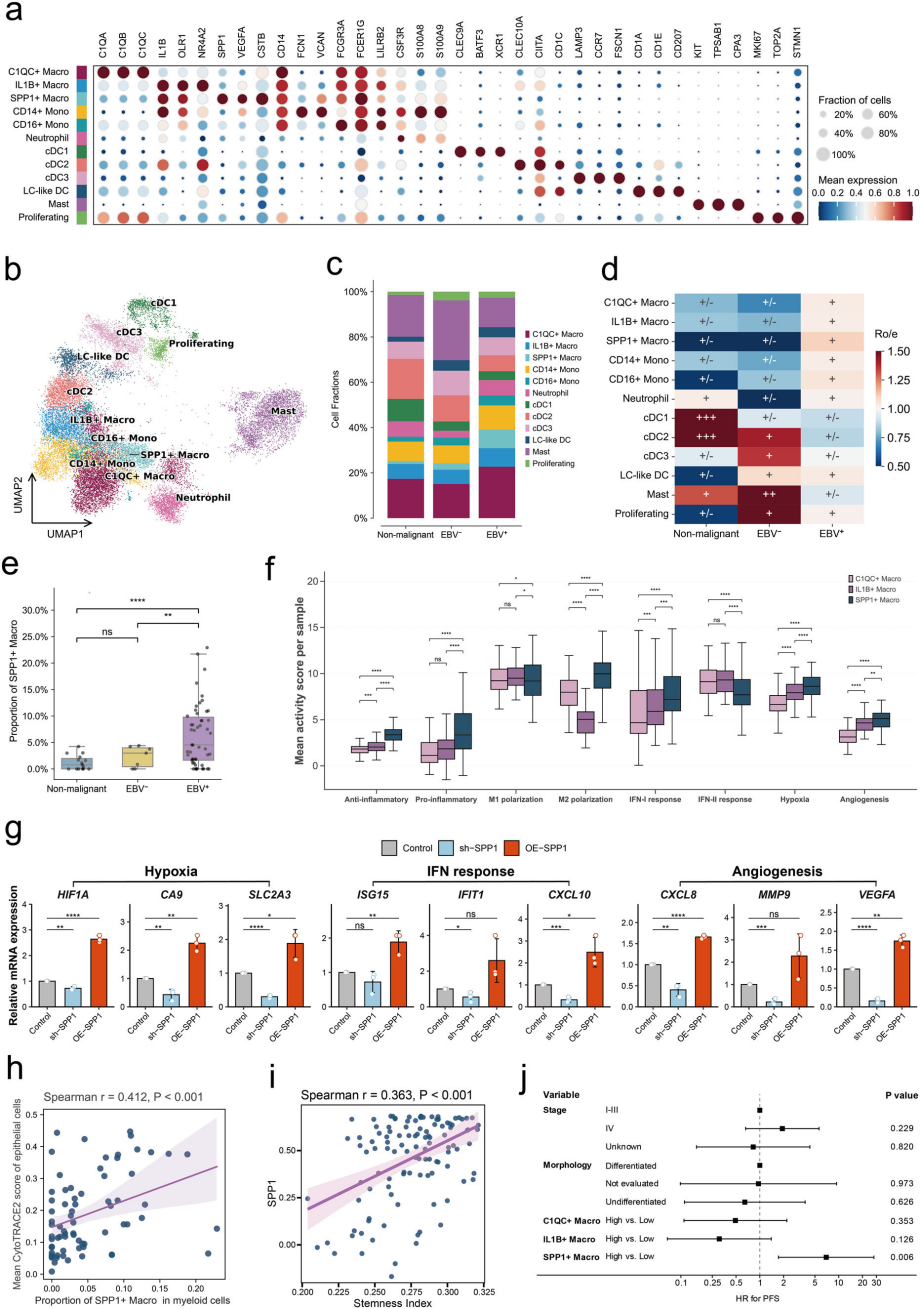
SPP1^+^ macrophages are enriched in EBV^+^ NPC and associated with tumor stemness and prognosis. (a) Dot plot of curated markers across the 12 myeloid subpopulations. (b) UMAP visualization of the myeloid subpopulations. (c) Relative composition of myeloid subpopulations in non‐malignant controls and EBV^−^ and EBV^+^ NPC. (d) Observed‐to‐expected enrichment of each myeloid subpopulation across disease groups. (e) Sample‐level proportion of SPP1^+^ macrophages within the myeloid compartment in non‐malignant controls, EBV^−^ NPC, and EBV^+^ NPC. (f) Sample‐level mean activity scores for the indicated functional programs in C1QC^+^, IL1B^+^, and SPP1^+^ macrophages. (g) qPCR validation of representative genes associated with hypoxia, interferon responses, and angiogenesis pathways in control, *SPP1*‐knockdown, and *SPP1*‐overexpressing macrophages. (h) Sample‐level correlation between the proportion of SPP1^+^ macrophages and the mean epithelial CytoTRACE2 score in the scRNA‐seq cohort. (i) Correlation between *SPP1* expression and the stemness index in the bulk RNA‐seq cohort. (j) Forest plot of multivariable Cox regression evaluating the associations of the indicated macrophage signatures with progression‐free survival. Squares indicate HRs, and horizontal lines indicate 95% CIs. Data in (g) are presented as mean ± SD from three independent biological replicates. Pairwise comparisons in (e,g) were performed using two‐sided unpaired Student's t‐tests. Pairwise comparisons in (f) were performed using two‐sided paired Wilcoxon signed‐rank tests. Correlations in (h,i) were assessed using two‐sided Spearman rank correlation. ns, not significant; *P < 0.05, **P < 0.01, ***P < 0.001, and ****P < 0.0001. Abbreviations: CI, confidence interval; EBV, Epstein–Barr virus; HR, hazard ratio; IFN, interferon; Macro, macrophage; Mono, monocyte; NPC, nasopharyngeal carcinoma; Ro/e, observed‐to‐expected ratio; scRNA‐seq, single‐cell RNA sequencing; UMAP, uniform manifold approximation and projection.

### 2.5 SPP1^+^ TAMs Are Associated with an Immunosuppressive T‐Cell Landscape in EBV^+^ NPC

Having linked SPP1^+^ TAM accumulation to epithelial stemness and adverse clinical outcomes, we next investigated whether it was also associated with remodeling of the T‐cell compartment. Canonical marker expression and functional gene‐program scores resolved 18 T/NK‐cell subpopulations (Figure S10a,b and Figure 5a–c). Their relative abundances differed among non‐malignant controls, EBV^−^ NPC, and EBV^+^ NPC (Figure 5d). Observed‐to‐expected enrichment analysis showed that CD8^+^ early and terminally exhausted T cells and both Treg subsets were enriched in EBV^+^ NPC (Figure 5e), paralleling the enrichment of SPP1^+^ TAMs in the same group. Across scRNA‐seq samples, the proportion of SPP1^+^ macrophages correlated positively with CD8^+^ terminally exhausted T (Tex) cells (Spearman r = 0.475, *P* < 0.001), TNFRSF9^+^ Tregs (r = 0.543, *P* < 0.001), and TNFRSF9^−^ Tregs (r = 0.448, *P* < 0.001), but not with CD8^+^ early Tex cells (r = 0.020, *P* = 0.869; Figure 5f). Consistently, in the bulk RNA‐seq cohort, the SPP1^+^ macrophage signature correlated positively with Tex and both Treg signatures, as well as with the T‐cell exhaustion score and the checkpoint genes *LAG3*, *HAVCR2*, and *CTLA4* (all *P* < 0.01), whereas its association with the early Tex signature was not significant (P = 0.179; Figure 5g).

**FIGURE 5:**
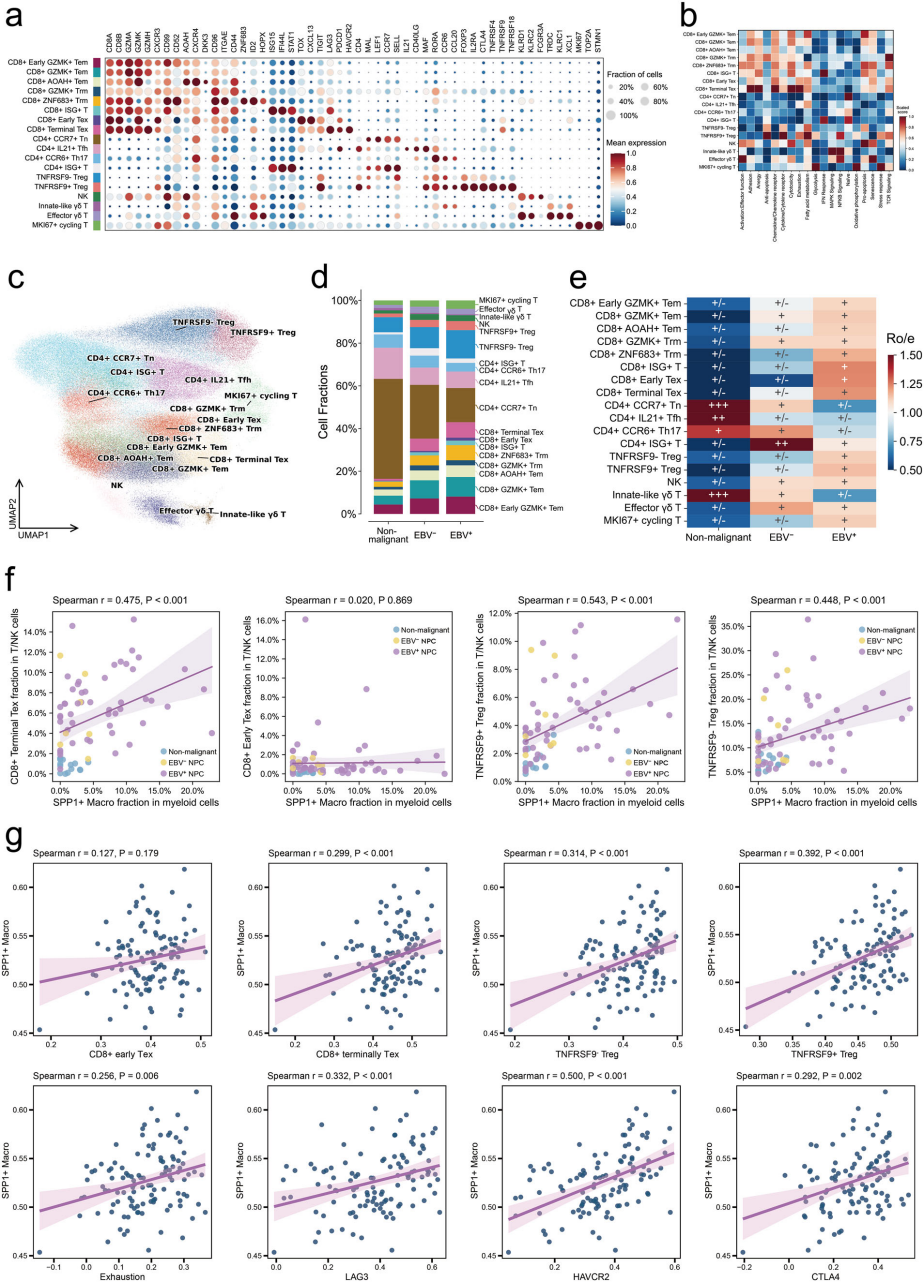
SPP1^+^ macrophages are associated with an immunosuppressive T‐cell landscape in EBV^+^ NPC. (a) Dot plot of curated markers across the 18 T/NK‐cell subpopulations. (b) Heatmap of scaled functional gene‐program scores across T/NK‐cell subpopulations. (c) UMAP visualization of the 18 T/NK‐cell subpopulations. (d) Relative composition of T/NK‐cell subpopulations in non‐malignant controls and EBV^−^ and EBV^+^ NPC. (e) Observed‐to‐expected enrichment of T/NK‐cell subpopulations across disease groups. (f) Sample‐level Spearman correlations between the proportion of SPP1^+^ macrophages and the proportions of CD8^+^ terminally Tex cells, CD8^+^ early Tex cells, TNFRSF9^+^ Tregs, and TNFRSF9^−^ Tregs in the scRNA‐seq cohort. (g) Spearman correlations between the SPP1^+^ macrophage signature and the indicated T‐cell‐state signatures, T‐cell exhaustion score, and inhibitory genes in the bulk RNA‐seq cohort. Each point represents one sample; shaded bands indicate 95% CIs around the fitted lines. All correlation tests were two‐sided. Abbreviations: CI, confidence interval; EBV, Epstein–Barr virus; ISG, interferon‐stimulated gene; NK, natural killer; NPC, nasopharyngeal carcinoma; Ro/e, observed‐to‐expected ratio; scRNA‐seq, single‐cell RNA sequencing; Tem, effector‐memory T cell; Tex, exhausted T cell; Tfh, follicular‐helper T cell; Tn, naïve T cell; Treg, regulatory T cell; Trm, tissue‐resident memory T cell; UMAP, uniform manifold approximation and projection.

### 2.6 ST Identifies an SPP1^+^ TAM‐Enriched Niche Linked to Tumor Stemness and Immunosuppression

Given the concurrent associations of SPP1^+^ TAMs with epithelial stemness and immunosuppressive T‐cell states, we next examined whether these populations co‐localized within spatially defined niches in EBV^+^ NPC. We analyzed 21,087 ST spots from 12 EBV^+^ NPC specimens. Sensitivity analyses across spatial‐radius cutoffs and Leiden resolutions supported a 300‐µm radius and a resolution of 0.2, yielding a stable eight‐niche solution (Figure S11a–c). Using these parameters, cross‐sample integration resolved eight spatial niches with heterogeneous representation across individual tumors (Figure S12 and Figure 6a–b). Mapping clinical response onto this landscape revealed distinct spatial distributions of complete response, partial response, and stable/progressive disease (Figure 6c). Niche5 was preferentially represented in stable/progressive disease but scarce in responding tumors, associating it with poor chemoimmunotherapy response (Figure 6d). To characterize the cellular composition of these spatial niches, we applied Tangram to deconvolve the ST data using subsampled cells from the annotated scRNA‐seq atlas as the reference (Figures S13 and S14). Cell‐type enrichment and fraction analyses showed that Niche5 was co‐enriched for tumor cells, SPP1^+^ macrophages, CD8^+^ terminally exhausted T cells, and TNFRSF9^+^ Tregs, together with IL1B^+^ macrophages, whereas C1QC^+^ macrophages were not preferentially enriched (Figure 6e,f). Transcription‐factor activity analysis showed that Niche 5 had relatively high inferred NFE2L2, HIF1A, NFKB, KDM5B, PPARG, and JUND activities, collectively supporting tumor stemness and immunosuppressive remodeling (Figure 6g). Hallmark scoring further showed relatively high activity of metabolic and stress‐adaptation programs, including fatty‐acid metabolism, glycolysis, oxidative phosphorylation, mTORC1 signaling, hypoxia, ROS, and apoptosis pathways (Figure 6h).

**FIGURE 6:**
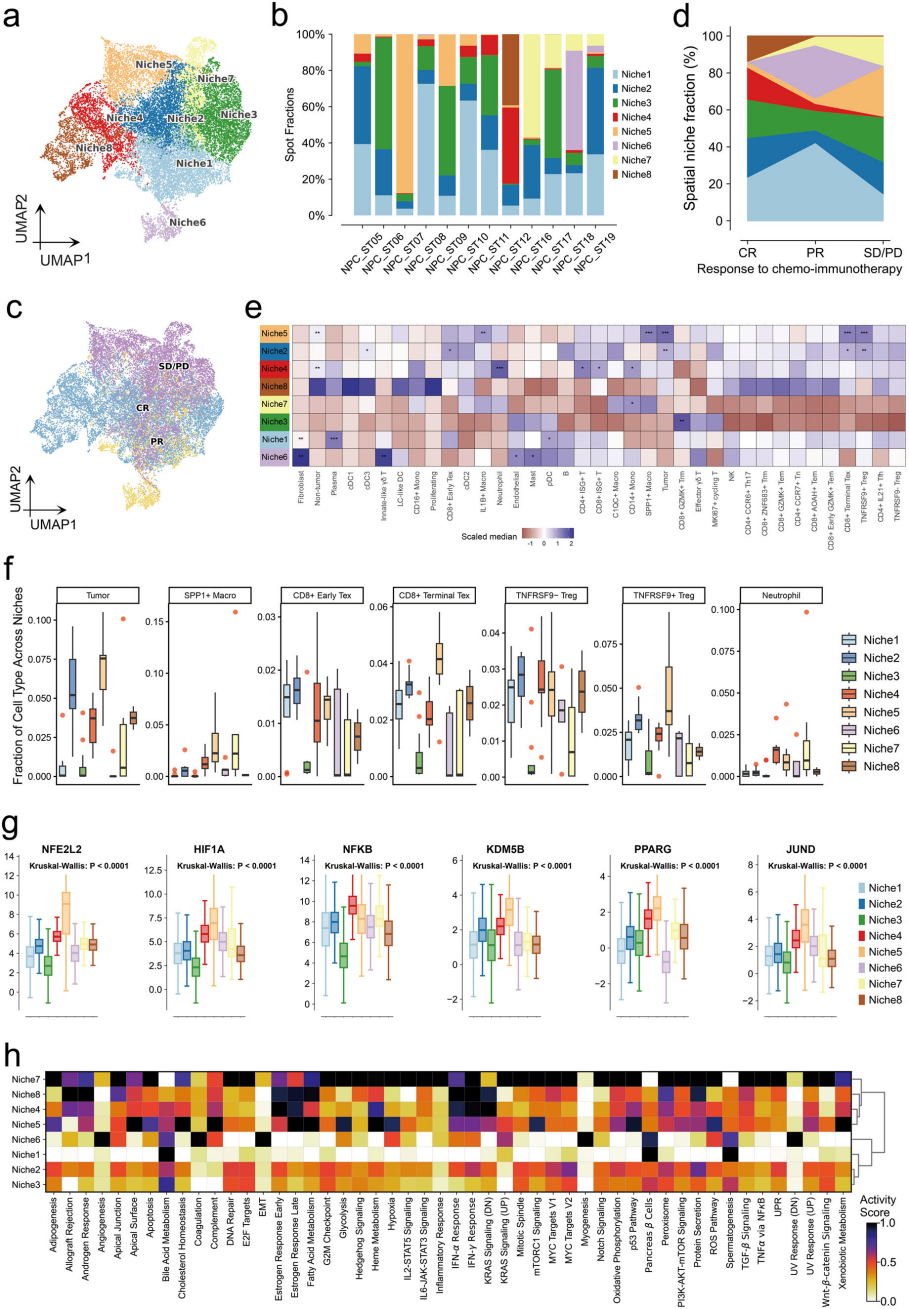
Spatial characterization of an SPP1^+^ TAM‐enriched niche in EBV^+^ NPC. (a) UMAP visualization of spatial transcriptomic spots from 12 EBV^+^ NPC specimens. (b) Relative niche composition of each spatial specimen. (c) UMAP visualization colored by clinical response to chemoimmunotherapy. (d) Relative niche composition across CR, PR, and SD/PD groups. (e) Heatmap of cell‐type enrichment across spatial niches. Color indicates the scaled sample–niche median inferred abundance, and asterisks indicate significances. (f) Distributions of the inferred fractions of the indicated cell populations across spatial niches. (g) Distributions of spot‐level activities of the indicated transcription factors across spatial niches. (h) Heatmap of scaled Hallmark gene set activities across spatial niches. *P < 0.05, **P < 0.01, and ***P < 0.001. For panel (e), pairwise comparisons between spatial niches were performed using one‐sided Wilcoxon rank‐sum tests, with *P* values adjusted using the Benjamini–Hochberg method. For panel (g), overall differences across spatial niches were evaluated using Kruskal–Wallis tests. Abbreviations: CR, complete response; EBV, Epstein–Barr virus; Macro, macrophage; Mono, monocyte; NPC, nasopharyngeal carcinoma; PR, partial response; SD/PD, stable or progressive disease; Tex, exhausted T cell; Treg, regulatory T cell; UMAP, uniform manifold approximation and projection.

### 2.7 EBV^+^ Tumor Cells Recruit and Program SPP1^+^ TAMs Through MIF‐Dependent Signaling

To identify upstream signals contributing to SPP1^+^ TAM accumulation, we compared intercellular communication networks between EBV^−^ and EBV^+^ NPC. EBV^+^ tumors exhibited a modest increase in the number of inferred interactions but slightly lower aggregate interaction strength, indicating remodeling rather than uniform enhancement of intercellular communication (Figure S15a). Within EBV^+^ tumors, SPP1^+^ macrophages exhibited the strongest outgoing interaction strength and were also among the populations with the highest incoming interaction strength (Figure S15b). Global pathway‐level comparison identified MIF and SPP1 among the signal pathways significantly increased in EBV^+^ tumors (Figure S15c). Focusing on the upstream tumor‐to‐macrophage direction, network visualization showed markedly enhanced MIF signaling from tumor cells to SPP1^+^ macrophages in EBV^+^ compared with EBV^−^ tumors (Figure S15d). Ligand–receptor analysis further demonstrated higher inferred communication probabilities for MIF–(CD74+CXCR4) and MIF–(CD74+CD44) between tumor cells and SPP1^+^ macrophages in the EBV^+^ group (Figure S15e). These findings prioritized tumor‐derived MIF as a candidate mediator of macrophage recruitment and SPP1^+^ TAM programming. This relationship was independently supported in the bulk RNA‐seq cohort, in which *MIF* and *SPP1* expression were positively correlated (Spearman r = 0.348, *P* < 0.001; Figure S16a). A high MIF signature was associated with shorter PFS (log‐rank *P* = 0.023; Figure S16b) and remained independently associated with PFS after adjustment (HR = 3.46, 95% CI = 1.07–11.1, *P* = 0.038; Figure S16c).

At the single‐cell level, EBV^+^ epithelial and tumor cells expressed higher levels of MIF and several chemotactic or macrophage‐regulatory genes, including CCL2–5, TGFB1, VEGFA, and CSF1, than their EBV^−^ counterparts (Figure 7a). Multiplex profiling of cell‐culture supernatants further demonstrated increased secretion of CCL3, CCL4, CCL5, and CXCL12 by EBV^+^ cell models (Figure 7b). Western blotting, qPCR, and ELISA further demonstrated increased cellular MIF protein, MIF transcript abundance, and MIF secretion in NP69‐EBV and HK1‐EBV cells relative to their parental counterparts, with high MIF levels also observed in C666‐1 cells (Figure 7c,d). Functionally, conditioned medium (CM) derived from EBV^+^ cell models promoted primary monocyte migration, whereas MIF098 attenuated this response; conversely, rhMIF alone was sufficient to enhance migration (Figure S17 and Figure 7e). In primary human monocyte‐derived macrophages (MDMs), CM from EBV^+^ and C666‐1 cells increased the mRNA and protein levels of SPP1, CD206, CD163, TGF‐β, and IL‐10 and enhanced SPP1 secretion. These effects were generally attenuated by MIF098, whereas rhMIF recapitulated the SPP1^+^ TAM‐like phenotype (Figure 7f–k). Consistent changes in marker expression and SPP1 secretion were independently reproduced in THP‐1‐derived macrophages (Figure 7l–q). Together, these findings identify tumor cell‐derived MIF as an important soluble mediator of EBV‐associated monocyte recruitment and SPP1^+^ macrophage programming.

**FIGURE 7:**
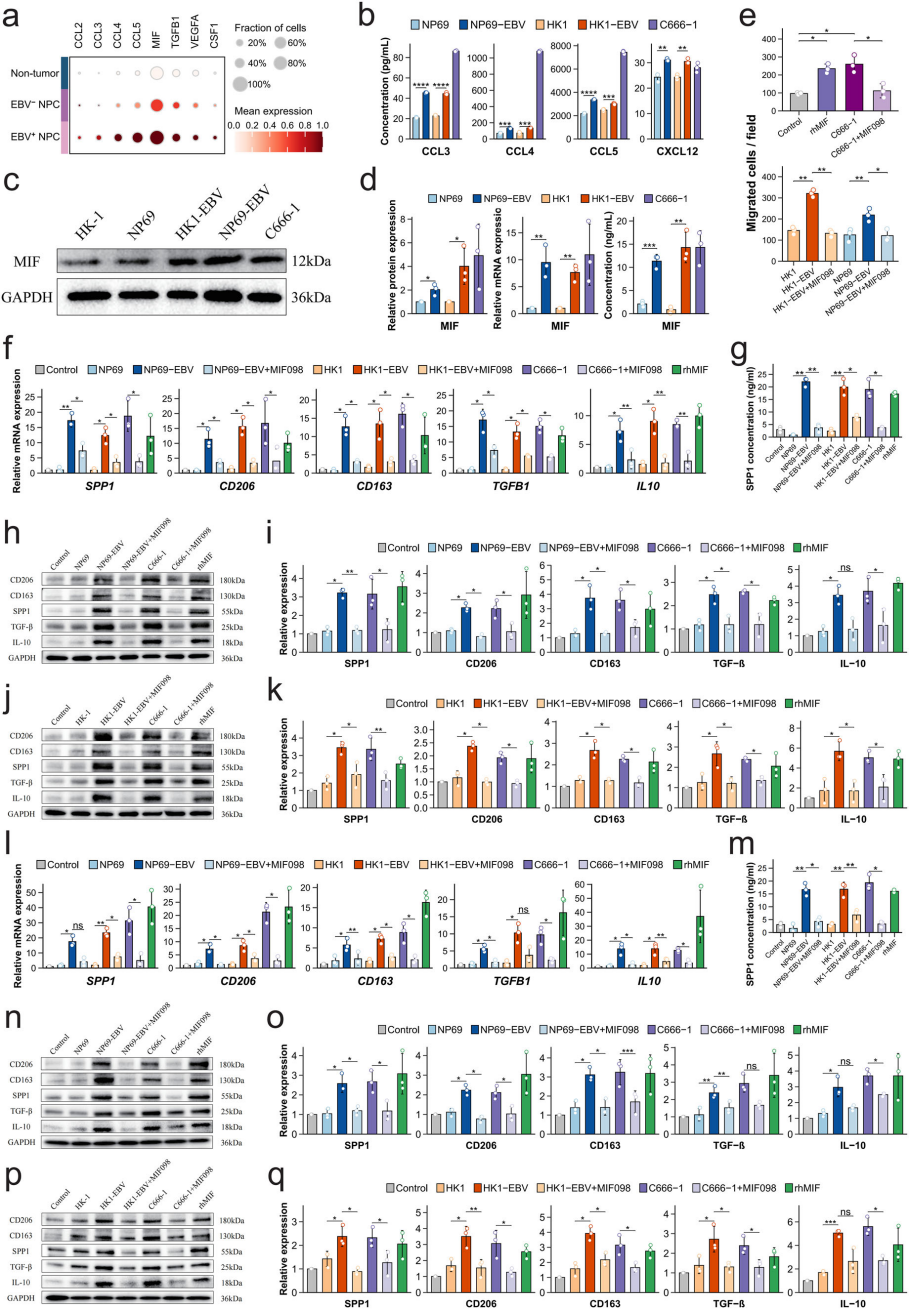
EBV^+^ cells recruit and program SPP1^+^ TAMs through MIF‐dependent signaling. (a) Dot plot showing the single‐cell expression of selected chemotactic and macrophage‐regulatory genes in epithelial cells from non‐malignant controls and EBV^−^ and EBV^+^ NPC. (b) Concentrations of CCL3, CCL4, CCL5, and CXCL12 in supernatants from the indicated epithelial and NPC cell models. (c) Representative cropped immunoblots of MIF in parental, EBV‐infected, and C666‐1 cells. (d) Densitometric quantification of MIF protein, qPCR analysis of MIF mRNA, and ELISA measurement of secreted MIF. (e) Quantification of primary human peripheral blood monocyte migration toward the indicated cell‐derived CM, with MIF098 added where indicated, or toward rhMIF. (f) qPCR analysis of *SPP1*, *CD206*, *CD163*, *TGFB1*, and *IL10* in primary human MDMs subjected to the indicated treatments. (g) ELISA measurement of SPP1 secretion by treated primary human MDMs. (h–k) Representative cropped immunoblots (h,j) and corresponding densitometric quantification (i,k) of SPP1, CD206, CD163, TGF‐β, and IL‐10 in primary human MDMs under the NP69‐based (h,i) or HK1‐based (j,k) treatment conditions. (l) qPCR analysis of *SPP1*, *CD206*, *CD163*, *TGFB1*, and *IL10* in THP‐1‐derived macrophages subjected to the indicated treatments. (m) ELISA measurement of SPP1 secretion by treated THP‐1‐derived macrophages. (n–q) Representative cropped immunoblots (n,p) and corresponding densitometric quantification (o,q) of SPP1, CD206, CD163, TGF‐β, and IL‐10 in THP‐1‐derived macrophages under the NP69‐based (n,o) or HK1‐based (p,q) treatment conditions. Unless otherwise indicated, quantitative data are presented as mean ± SD from three independent biological replicates. For the Transwell migration assay in (e), each biological replicate included three technical replicates, which were averaged before statistical analysis. Prespecified pairwise comparisons were evaluated using two‐sided Student's t‐tests. Paired tests were used for donor‐matched primary‐cell experiments in (e–k) and experiment‐matched THP‐1‐derived macrophage experiments in (l–q), whereas unpaired tests were used for the remaining comparisons in (b,d). ns, not significant; *P < 0.05, **P < 0.01, ***P < 0.001, and ****P < 0.0001. Abbreviations: CM, conditioned medium; EBV, Epstein–Barr virus; ELISA, enzyme‐linked immunosorbent assay; MDM, monocyte‐derived macrophage; MIF, macrophage migration inhibitory factor; MIF098, a small‐molecule MIF inhibitor; NPC, nasopharyngeal carcinoma; qPCR, quantitative polymerase chain reaction; rhMIF, recombinant human MIF; SPP1, secreted phosphoprotein 1; TAM, tumor‐associated macrophage.

### 2.8 Validation of SPP1^+^ Macrophage‐Associated Tumor Stemness and CD8^+^ T‐Cell Dysfunction

We next investigated the downstream consequences of SPP1^+^ TAM accumulation. Network visualization showed that SPP1 signaling from SPP1^+^ macrophages toward tumor cells and CD8^+^ T cells was markedly increased in EBV^+^ compared with EBV^−^ tumors (Figure S15d). Ligand–receptor analysis linked enhanced SPP1^+^ macrophage communication with tumor and CD8^+^ terminally exhausted T cells in EBV^+^ tumors to SPP1–CD44 and SPP1–integrin interactions (Figure S15e). Spatial communication analysis provided complementary evidence linking this signaling axis to treatment response. Non‐responder niches exhibited greater overall inferred cell–cell communication strength than responder niches (Figure S15f), with SPP1^+^ macrophages acting as prominent signal‐sending and signal‐receiving populations (Figure S15g). Pathway‐level comparisons further identified MIF and SPP1 signaling as significantly enhanced in non‐responder niches (Figure S15h).

To evaluate the tumor‐directed effects of macrophage‐derived SPP1, we generated SPP1‐knockdown and *SPP1*‐overexpressing macrophages and confirmed the corresponding changes in cellular and secreted SPP1 (Figure 8a,b). CM from *SPP1*‐overexpressing macrophages increased the mRNA and protein expression of SOX2, CD44, NANOG, and OCT‐4 in NP69‐EBV, HK1‐EBV, and C666‐1 cells (Figure 8c,d). Recombinant SPP1 produced similar effects and increased these markers when added to CM from SPP1‐knockdown macrophages. CM from *SPP1*‐overexpressing macrophages and recombinant SPP1 also enhanced cell viability and invasion (Figure 8e,f) and increased tumorsphere formation in HK1‐EBV and C666‐1 cells (Figure 8g). By contrast, CM from SPP1‐knockdown macrophages alone produced comparatively limited or inconsistent changes. At the tissue level, descriptive multiplex immunofluorescence analysis showed higher densities of CD206^+^SPP1^+^ macrophages and EPCAM^+^MIF^+^, EPCAM^+^SOX2^+^, and EPCAM^+^MIF^+^SOX2^+^ epithelial populations in the examined EBV^+^ section than in the EBV^−^ section (Figure 8h,i). These findings spatially associate SPP1^+^ macrophage accumulation with MIF‐expressing and stemness‐associated epithelial states.

**FIGURE 8:**
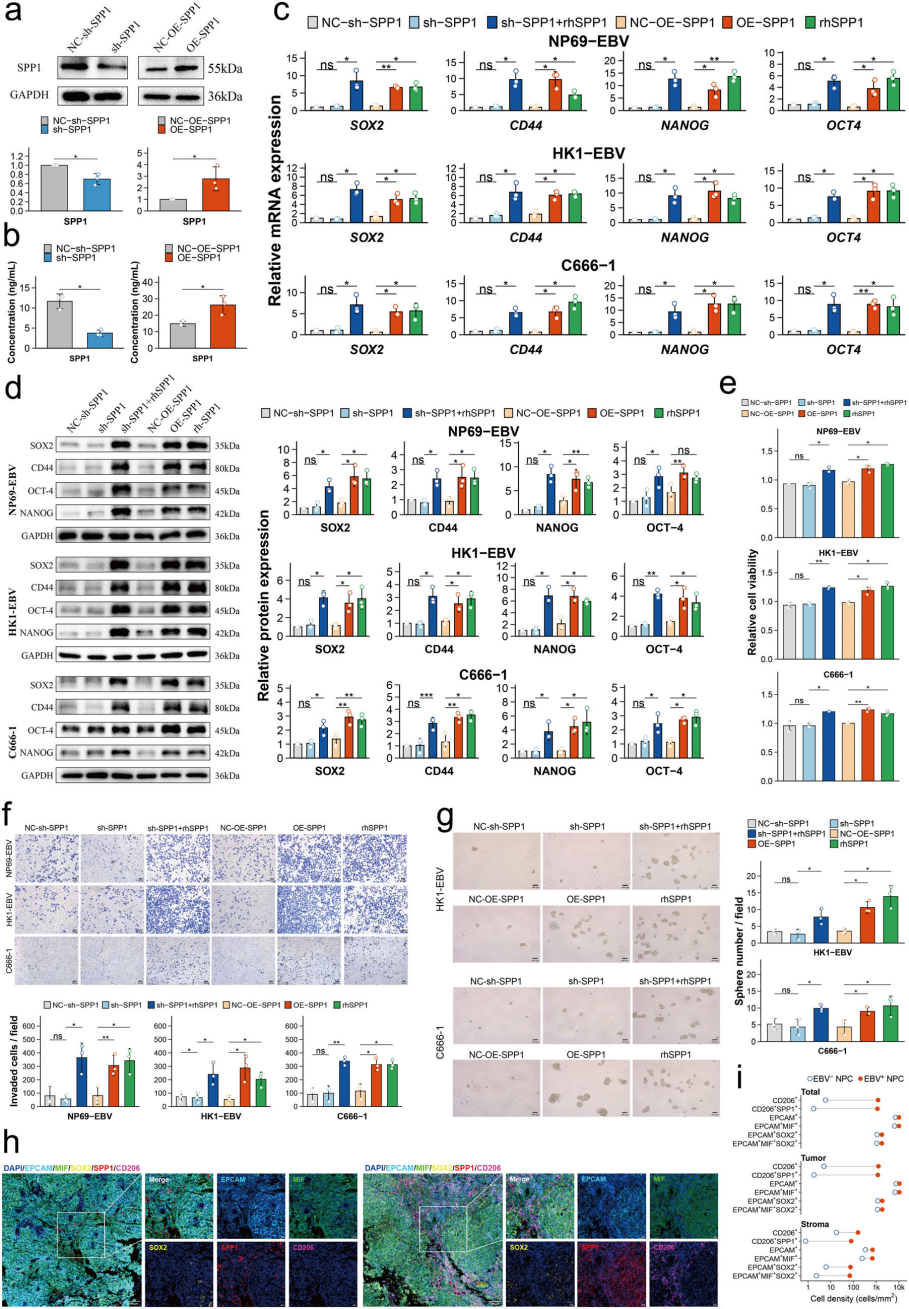
Macrophage‐derived SPP1 promotes stemness‐associated phenotypes in NPC. (a) Representative cropped immunoblots and densitometric quantification confirming SPP1 knockdown and overexpression in macrophages. (b) ELISA measurement of SPP1 in macrophage CM. (c) qPCR analysis of *SOX2*, *CD44*, *NANOG*, and *OCT4* in NP69‐EBV, HK1‐EBV, and C666‐1 cells treated with CM from *SPP1*‐manipulated macrophages or with rhSPP1. (d) Representative cropped immunoblots and densitometric quantification of SOX2, CD44, NANOG, and OCT‐4 in the indicated cells and treatment groups. (e) CCK‐8 analysis of cell viability. (f) Representative Transwell invasion images and quantification of invaded cells per field. Scale bars, 100 µm. (g) Representative tumorsphere images and quantification of sphere numbers in HK1‐EBV and C666‐1 cells. Scale bars, 100 µm. (h) Representative multiplex immunofluorescence images of EBV^−^ (left) and EBV^+^ (right) NPC tissues. Scale bars, 100 µm in the overview images and 20 µm in the enlarged images. (i) Densities of CD206^+^, CD206^+^SPP1^+^, EPCAM^+^, EPCAM^+^MIF^+^, EPCAM^+^SOX2^+^, and EPCAM^+^MIF^+^SOX2^+^ populations in the total, tumor, and stromal compartments of the analyzed EBV^−^ and EBV^+^ tissue sections. Quantitative data in (a–g) are presented as mean ± SD from three independent biological replicates. For the Transwell invasion and tumorsphere assays in (f–g), each biological replicate included three technical replicates, which were averaged before statistical analysis. Prespecified pairwise comparisons were performed using two‐sided unpaired Student's t‐tests. Panel (i) presents descriptive density measurements; no inferential statistical test was applied. ns, not significant; *P < 0.05, **P < 0.01, ***P < 0.001, and ****P < 0.0001. Abbreviations: CCK‐8, Cell Counting Kit‐8; CM, conditioned medium; DAPI, 4′,6‐diamidino‐2‐phenylindole; EBV, Epstein–Barr virus; ELISA, enzyme‐linked immunosorbent assay; MIF, macrophage migration inhibitory factor; NC, negative control; NPC, nasopharyngeal carcinoma; OE, overexpression; rhSPP1, recombinant human SPP1; sh, short hairpin RNA; SPP1, secreted phosphoprotein 1.

We next examined whether macrophage‐derived SPP1 impaired CD8^+^ T‐cell function. Exposure to CM from *SPP1*‐overexpressing macrophages or to recombinant SPP1 shifted CD8^+^ T cells toward fewer divisions and reduced their CFSE‐based proliferation index (Figure 9a). CD44 blockade substantially restored proliferation, whereas recombinant SPP1 supplementation in the *SPP1*‐knockdown setting reinstated the suppressive phenotype. These treatments also increased the exhaustion‐associated markers PD‐1, TIM‐3, and LAG‐3 (Figure 9b), while reducing the effector cytokines IFN‐γ, TNF, and IL‐2 (Figure 9c) and the cytotoxic molecules perforin and GZMB (Figure 9d). These changes were partially reversed by CD44 blockade, supporting a contribution of SPP1–CD44 signaling to macrophage‐mediated CD8^+^ T‐cell dysfunction.

**FIGURE 9:**
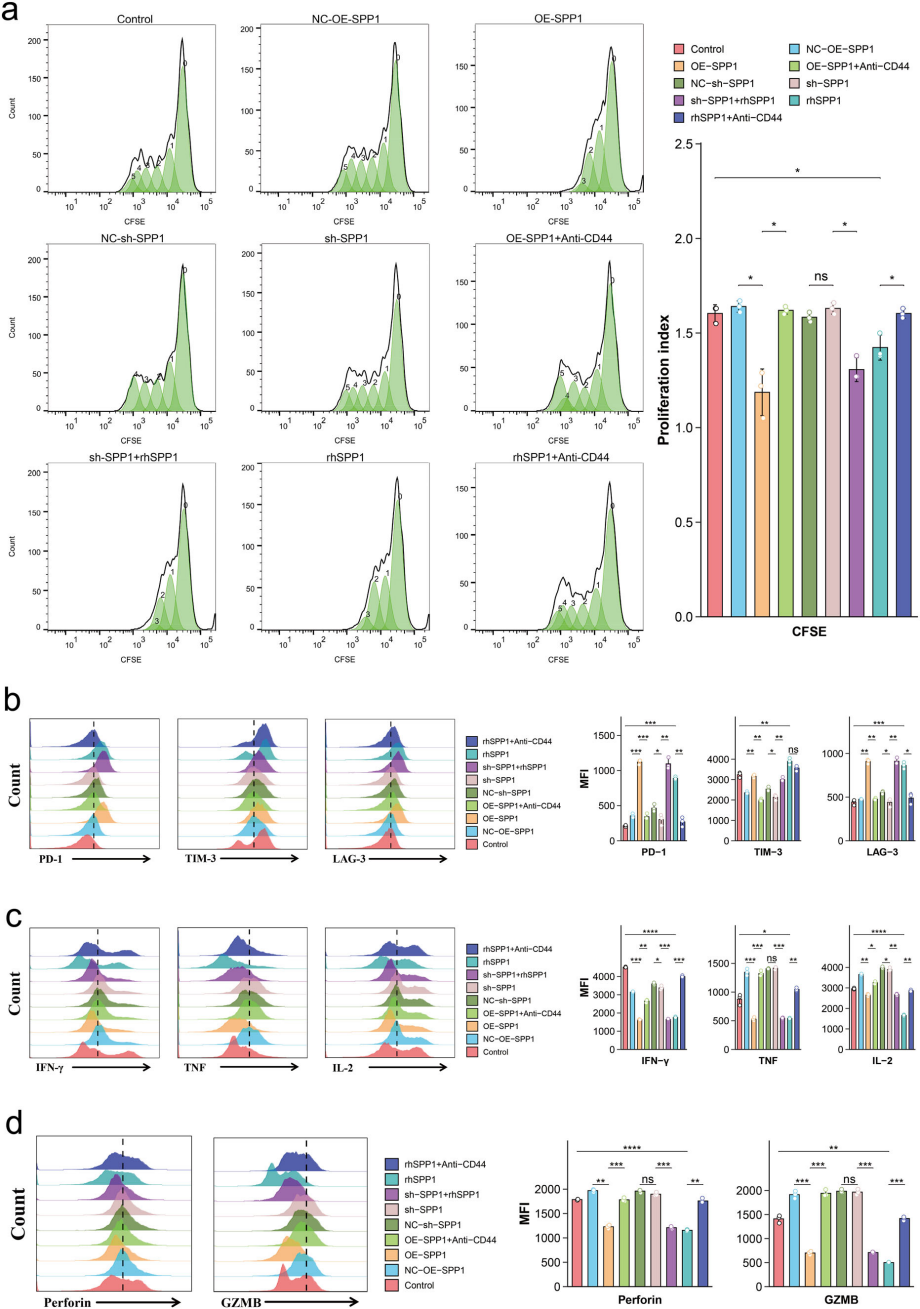
Macrophage‐derived SPP1 suppresses CD8^+^ T‐cell function. (a) Representative CFSE division profiles and quantification of the proliferation index. Numbers indicate successive cell divisions. (b) Representative flow‐cytometry distributions and MFI quantification of the exhaustion‐associated markers PD‐1, TIM‐3, and LAG‐3. (c) Representative distributions and MFI quantification of the effector cytokines IFN‐γ, TNF, and IL‐2. (d) Representative distributions and MFI quantification of the cytotoxic molecules perforin and GZMB. CD8^+^ T cells were exposed to CM from *SPP1*‐manipulated macrophages or to rhSPP1, with or without CD44 blockade. Quantitative data are presented as mean ± SD from three independent biological replicates. Prespecified pairwise comparisons were performed using two‐sided paired t‐tests. ns, not significant; *P < 0.05, **P < 0.01, ***P < 0.001, and ****P < 0.0001. Abbreviations: CFSE, carboxyfluorescein succinimidyl ester; CM, conditioned medium; GZMB, granzyme B; MFI, mean fluorescence intensity; NC, negative control; OE, overexpression; rhSPP1, recombinant human SPP1; sh, short hairpin RNA; SPP1, secreted phosphoprotein 1.

## 3 Discussion

Through integrated single‐cell, spatial, and bulk transcriptomic analyses, together with functional validation, we found that a conserved SPP1^+^ TAM transcriptional state previously described across multiple solid tumor types was preferentially enriched in EBV^+^ NPC and was associated with stemness‐related epithelial phenotypes, dysfunctional T‐cell states, and shorter PFS. The main advance of this study does not lie in identifying SPP1^+^ TAMs as a previously unrecognized macrophage population. Rather, our findings connect this conserved TAM program to an EBV‐associated upstream signal, define its spatial organization with tumor and T‐cell states, and examine its functional effects on both tumor cells and primary CD8^+^ T cells. Our findings support bidirectional crosstalk involving tumor‐derived MIF and macrophage‐derived SPP1. EBV^+^ tumor cells showed increased MIF expression and secretion, and experiments with MIF098 and rhMIF implicated MIF in monocyte recruitment and SPP1^+^ TAM‐like programming. Conversely, CM from *SPP1*‐overexpressing macrophages and rhSPP1 enhanced stemness‐marker expression, cell viability, invasion, and tumorsphere formation in EBV^+^ epithelial and tumor models. Macrophage‐derived SPP1 also impaired CD8^+^ T‐cell proliferation and effector and cytotoxic functions while increasing exhaustion‐associated markers, effects that were partially reversed by CD44 blockade. Spatial analyses identified Niche 5 as an SPP1^+^ TAM‐enriched ecosystem containing tumor cells, CD8^+^ terminally exhausted T cells, and TNFRSF9^+^ Tregs; this niche was more frequent in tumors with SD/PD. Multiplex immunofluorescence further associated CD206^+^SPP1^+^ macrophages with MIF‐expressing and stemness‐associated epithelial populations, while bulk transcriptomic analyses linked high SPP1^+^ macrophage and MIF signatures to shorter PFS. Collectively, these findings support an EBV‐associated MIF–SPP1 macrophage–tumor/T‐cell crosstalk model that connects tumor stemness with immune dysfunction and provides a rationale for therapeutic evaluation of this signaling axis in NPC.

EBV remodels the NPC microenvironment through multifaceted interactions between infected tumor cells and the surrounding stromal and immune compartments [29, 30, 31]. Viral latent proteins such as LMP1 and LMP2A activate oncogenic PI3K–AKT–mTOR and NF‐κB signaling cascades, thereby driving epithelial–mesenchymal transition and the acquisition of stem‐like properties [32, 33]. EBV infection also induces the secretion of VEGF and GM‐CSF, which in turn promote angiogenesis and myeloid infiltration and help establish an inflammatory yet immunosuppressive microenvironment [34]. LMP1‐mediated metabolic reprogramming has been linked to myeloid‐derived suppressor‐cell expansion, whereas EBV‐induced ATR activation has been associated with M2‐like macrophage polarization [35, 36]. Previous NPC studies have therefore established that EBV can promote epithelial stem‐like properties and reshape the myeloid compartment. However, how these tumor‐derived signals connect to the recurrent SPP1^+^ TAM program and its downstream effects on tumor and T‐cell states remains less clear. Here, we provide evidence that EBV^+^ epithelial‐cell‐derived MIF contributes to monocyte recruitment and SPP1‐associated TAM‐like programming. Together, these observations support bidirectional crosstalk in which tumor‐derived MIF contributes to monocyte recruitment and SPP1^+^ macrophage programming, while macrophage‐derived SPP1 promotes stemness‐associated tumor phenotypes.

SPP1^+^ TAMs are not unique to NPC but represent a broadly shared macrophage transcriptional state across multiple solid tumor types. Across diverse tumor types, this SPP1‐high macrophage program has been associated with stromal remodeling, angiogenesis, tumor progression, and impaired antitumor immunity [37, 38, 39, 40, 41, 42]. Our study builds on previous works by linking the recurrent SPP1^+^ TAM program to an EBV‐associated upstream signal and defining its spatial and functional relationships with both tumor and T‐cell states in NPC. In the EBV^+^ spatial cohort, SPP1^+^ TAMs, stemness‐associated tumor cells, exhausted CD8^+^ T cells, and Tregs were enriched together in Niche 5. This niche was more frequent in tumors with SD/PD than in those with CR/PR, suggesting an association with poor chemoimmunotherapy response. However, because the spatial cohort included only EBV^+^ tumors, these data do not establish that Niche 5 is EBV‐specific or that it directly causes treatment resistance. Functionally, CM from *SPP1*‐overexpressing macrophages and rhSPP1 increased stemness‐associated marker expression and tumorsphere formation, and rhSPP1 add‐back restored these effects in the SPP1‐knockdown setting. Because *SPP1* knockdown alone produced limited or inconsistent changes, these experiments support a role for elevated macrophage‐derived SPP1 in promoting stemness‐associated phenotypes but do not establish its absolute requirement. Previous studies have shown that SPP1^+^ macrophages can promote tumor progression through different paracrine mechanisms. In liver cancer, this effect was linked to a VTN–CCL15 macrophage–tumor circuit, [43] whereas in head and neck squamous cell carcinoma, SPP1^+^ macrophages acted partly through TNF‐α and IL‐1β [39]. Whereas these studies highlight context‐dependent paracrine mediators released by SPP1^+^ macrophages, our results show that elevated macrophage‐derived SPP1 itself is sufficient to promote stemness‐associated phenotypes in EBV^+^ epithelial and tumor cells under the tested conditions.

Macrophage‐derived SPP1 and rhSPP1 also reduced primary CD8^+^ T‐cell proliferation and effector and cytotoxic functions while increasing exhaustion‐associated markers. CD44 blockade substantially restored proliferation and partially reversed these changes. This result is consistent with studies in liver metastases and ovarian cancer showing that SPP1^+^ macrophages impair CD8^+^ T‐cell function through SPP1–CD44 signaling [44, 45]. Our study extends these observations to EBV‐related NPC using primary human CD8^+^ T cells and shows that macrophage‐derived soluble SPP1 is sufficient to impair CD8^+^ T‐cell function under cell‐free conditions. However, additional contact‐dependent interactions in vivo cannot be excluded. Together, these findings connect an EBV‐associated upstream signal with the tumor‐directed and T‐cell‐directed effects of a conserved SPP1^+^ TAM program, without establishing a closed feed‐forward loop. The antitumor activity of targeting SPP1‐high myeloid cells in preclinical models provides a rationale for evaluating this axis in NPC [40]. Whether such targeting can enhance the efficacy of immune‐checkpoint blockade remains to be determined.

## 4 Limitations

Despite its comprehensive multi‐omic design and experimental validation, this study has several limitations. First, although our functional experiments included primary human monocytes, monocyte‐derived macrophages, primary CD8^+^ T cells, THP‐1‐derived macrophages, and established epithelial and NPC cell lines, the conditioned‐medium assays used cell‐free supernatants and therefore evaluated only soluble‐factor‐mediated effects. These experiments demonstrate that soluble mediators, including tumor‐derived MIF and macrophage‐derived SPP1, are sufficient to induce the observed phenotypes under the tested conditions. However, they do not capture direct cell–cell contact, phagocytosis, antigen presentation, juxtacrine signaling, or other contact‐dependent mechanisms and therefore cannot exclude their additional contributions in vivo. Future organoid–myeloid co‐culture and three‐dimensional models incorporating direct cellular interactions would provide a more physiologically relevant assessment of this signaling axis. Second, we did not perform an in‐depth mechanistic characterization of the intracellular signaling pathways and transcriptional networks downstream of the MIF–SPP1 axis. Although our in vitro findings support bidirectional communication between EBV^+^ tumor cells and SPP1^+^ macrophages, the transcriptional, metabolic, and epigenetic mechanisms underlying macrophage programming and tumor stemness remain to be elucidated. Third, there is currently no established physiologically relevant animal model of NPC that faithfully recapitulates EBV‐associated tumorigenesis and immune interactions. Consequently, the functional relevance of the EBV–MIF–SPP1 signaling axis and the causal role of SPP1^+^ TAMs in promoting tumor stemness and immune suppression could not be verified in vivo. This limitation underscores the need for EBV‐permissive or humanized NPC models for mechanistic validation and therapeutic testing. Fourth, EBV^−^ specimens were limited across the study cohorts. Despite cohort expansion, the scRNA‐seq dataset included only seven EBV^−^ NPC cases and remained unbalanced by EBV status. Multiplex immunofluorescence was limited to one EBV^−^ and one EBV^+^ case and did not include CD68, precluding inferential or stage‐stratified comparisons, while insufficient fresh EBV^−^ tissue prevented matched flow‐cytometric or cell‐sorting validation. Moreover, the spatial treatment cohort comprised only EBV^+^ tumors; therefore, whether Niche 5 is specific to EBV‐associated NPC remains unresolved. Validation in larger, EBV‐status‐balanced cohorts is required. Finally, although our findings support contributions of MIF and SPP1–CD44 signaling to macrophage programming and CD8^+^ T‐cell dysfunction, the association between SPP1^+^ TAM enrichment and treatment resistance remains correlative. This axis was not perturbed in vivo during PD‐1 blockade; further studies incorporating functional genomics, macrophage‐specific targeting, and appropriate preclinical models will therefore be required to establish its therapeutic relevance.

## 5 Conclusion

In summary, this study defines an EBV‐associated MIF–SPP1 macrophage–tumor/T‐cell crosstalk model in NPC. Integrative single‐cell, spatial, bulk‐transcriptomic, and functional analyses showed that SPP1^+^ TAMs were enriched in EBV^+^ NPC and associated with stemness‐related epithelial states, dysfunctional T‐cell populations, and shorter progression‐free survival. Within the EBV^+^ spatial cohort, SPP1^+^ TAMs co‐occurred with stemness‐associated tumor cells, exhausted CD8^+^ T cells, and Tregs in Niche 5, which was more frequent in tumors with poor chemoimmunotherapy response. Functional experiments further supported bidirectional signaling in which tumor‐derived MIF promoted monocyte recruitment and SPP1‐associated TAM‐like programming, whereas macrophage‐derived SPP1 enhanced tumor stemness‐associated phenotypes and impaired primary CD8^+^ T‐cell function under the tested conditions. Together, these findings link an EBV‐associated macrophage program to tumor plasticity, immune dysfunction, and adverse clinical outcomes, providing a rationale for further therapeutic evaluation of the MIF–SPP1 axis in NPC.

## 6 Methods

### 6.1 Study Participants and Ethical Approval

For scRNA‐seq analysis, treatment‐naïve NPC tumor biopsies were prospectively collected from six patients enrolled in the ChiCTR2000032317 clinical trial at Sun Yat‐sen University Cancer Center (SYSUCC) [27, 46]. To expand the cohort and increase statistical power, these data were integrated with five previously published scRNA‐seq datasets, including four generated at SYSUCC and one from Peking University Shenzhen Hospital and Shenzhen People's Hospital [23, 24, 25, 26, 28]. We excluded one recurrent case and one patient with an uncertain EBV infection status, resulting in a final cohort of 72 samples for downstream analyses, including 15 normal controls, 7 EBV^−^ NPCs, and 50 EBV^+^ NPCs. For ST analysis, formalin‐fixed paraffin‐embedded (FFPE) tumor samples were also obtained from 12 patients with EBV^+^ NPC at SYSUCC (Table S2) [47]. The 10× Genomics scRNA‐seq and 10X Visium ST assays were performed as previously described [24, 25, 26, 27, 47]. For mIC staining, FFPE specimens from one EBV^−^ and one EBV^+^ NPC case at SYSUCC were analyzed to provide preliminary protein‐level validation of the transcriptomic findings (Table S3). An independent NPC cohort with bulk RNA‐seq and accompanying clinical data was further analyzed to assess clinicopathological associations and prognostic relevance (Table S4) [48]. All patients were histopathologically diagnosed with primary NPC according to the World Health Organization classification. EBV positivity was confirmed by in situ hybridization for Epstein–Barr virus–encoded small RNAs (EBERs) in tumor biopsy specimens. Clinical staging was determined based on the eighth edition of the Union for International Cancer Control classification. Studies involving newly collected SYSUCC specimens were approved by the Institutional Review Board of SYSUCC. For the previously published datasets, ethics approval was obtained by the original investigators from their respective institutions. Written informed consent was obtained from all participants or waived by the relevant ethics committee, as applicable. All procedures were conducted in accordance with the Declaration of Helsinki.

### 6.2 Single‐cell RNA‐seq Data Preprocessing

Raw sequencing data were processed using the Cell Ranger pipeline (10x Genomics, USA) to perform alignment, filtering, and UMI counting, yielding gene‐by‐cell expression matrices. These count matrices were subsequently merged and analyzed using the Omicverse framework (v2.2.1) [49]. Doublet removal was performed using Scrublet with an expected doublet rate of 5%, applying an automatically determined threshold for each sample to exclude predicted doublets while retaining high‐confidence singlets. Subsequent quality control filtered cells based on well‐established thresholds: ≥ 500 UMIs, ≥ 200 detected genes, and ≤ 20% mitochondrial gene content. Highly variable genes (HVGs) were identified using a two‐step normalization strategy, involving shift‐log normalization to a target sum of 5×10^5^ transcripts per cell, followed by HVG selection based on Pearson residual variance. A total of 2,000 HVGs were retained for downstream dimensionality reduction. Principal component analysis was performed on scaled expression values stored in the scaled layer. The top 30 principal components were used to compute the neighborhood graph. Cell embeddings were generated using the UMAP algorithm. Graph‐based clustering was performed using the Leiden algorithm on the shared nearest‐neighbor graph with a resolution of 1.5, which is adequate for first‐round clustering based our experience. To ensure comparability across samples and mitigate technical variability, we conducted data integration and batch correction benchmarking using the scIB framework (v0.5.5) [50]. Initial inspection of the integrated dataset revealed substantial batch‐associated variation. We systematically evaluated multiple batch correction algorithms, including Harmony, cellANOVA, scANVI, ComBat, scVI, CONCORD, CCA, and Scanorama. Each method generated low‐dimensional embeddings, which were visualized using UMAP and quantitatively assessed via a suite of scIB benchmarking metrics. Integration performance was evaluated across multiple dimensions, including biological conservation and batch correction metrics. The approach that demonstrated the most balanced and robust performance was selected for final data integration.

### 6.3 Manual Cell Type Annotation

We manually annotated eight major cell lineages based on canonical marker expression and top‐ranked differentially expressed genes identified by one‐vs.‐rest Wilcoxon rank‐sum tests. Epithelial cells were identified by EPCAM, KRT8, and KRT19; fibroblasts by FN1, COL1A1, and CALD1; endothelial cells by PECAM1, VWF, and CLDN5; T/NK cells by CD3D, CD3E, and CD3G; B cells by CD79A, CD79B, and MS4A1; plasma cells by CD38, MZB1, and JCHAIN; myeloid cells by TYROBP, CST3, and LYZ; and plasmacytoid dendritic cells by IRF7, IL3RA, and LILRA4. Marker expression was visualized across clusters using dot and violin plots, with ambiguous cases further refined via literature and reference atlas comparison. Myeloid and T/NK cells were separately subsetted and subjected to iterative reclustering for higher‐resolution annotation. Clustering was performed at compartment‐specific resolutions, with additional rounds of subclustering applied where necessary to resolve transcriptionally distinct populations. Subclusters were manually annotated based on canonical marker expression and top‐ranked differentially expressed genes identified by one‐vs.‐rest Wilcoxon rank‐sum tests, with reference to established myeloid and T/NK cell‐state classifications from published pan‐cancer and disease‐specific single‐cell studies [17, 51, 52, 53]. Functional gene‐program scores were additionally used to distinguish closely related T/NK‐cell states [53].

### 6.4 Transcriptional Divergence Analysis

To quantify transcriptional divergence between EBV^+^ and EBV^−^ NPCs within defined cell types, we computed pairwise group distances using the distance module implemented in PertPy [54]. Specifically, we applied the Euclidean metric to compare the relative positions of cell populations in a common low‐dimensional space derived via PCA, using the top components from the PCA representation. For each cell type, distance calculations were performed between cells stratified by EBV status. The Euclidean distance here corresponds to the L^2^ norm between group‐wise centroids in PCA space, approximating pseudobulk‐level differences. While not accounting for intragroup variability, this metric offers an intuitive measure of the global shift in transcriptional state attributable to EBV infection.

### 6.5 Observed‐to‐Expected Cell‐Type Enrichment Analysis

To quantify differences in the relative representation of cell populations among disease groups, we calculated the ratio of observed to expected cell numbers (Ro/e) following previously described approaches [17]. Contingency tables of cell counts were constructed for each combination of cell population and disease group. Under the assumption that cell‐population identity and disease group were independent, the expected cell count was calculated as follows: *E_ij_* =  *N*_*i*._ *N*_.*j*_/*N*; where *E_ij_* represents the expected number of cells belonging to cell population i in disease group j; *N*_*i*. _ represents the total number of cells belonging to cell population i; *N*_.*j*_ represents the total number of cells in disease group j; and N represents the total number of cells included in the analysis. The enrichment ratio was then calculated as follows: *R*_o/e_  =  *O*_ij_ / *E_ij_*; where O_ij_ and *E_ij_* represent the observed and expected cell numbers, respectively. Ro/e values greater than 1 indicated relative enrichment, whereas values below 1 indicated relative underrepresentation. For visualization, Ro/e values were categorized as +/− (< 1), + (1–< 1.5), ++ (1.5–< 2), or +++ (≥ 2) and displayed as heat maps. Analyses were performed separately for the myeloid and T/NK compartments.

### 6.6 Copy Number Variation (CNV) Inference and Malignant Cell Identification

To identify putative malignant epithelial cells, we inferred large‐scale chromosomal CNVs from single‐cell transcriptomic profiles using a smoothing‐based approach implemented in the infercnvpy module [55]. The non‐malignant epithelial cells from non‐tumor controls were designated as the normal reference population. Gene positions were mapped using the GENCODE v48 GTF annotation, and smoothed CNV profiles were computed using a sliding window strategy (window size  =  500) across the genome. This enabled the construction of chromosome‐level relative expression maps and quantification of per‐cell CNV deviation. To facilitate inter‐cellular comparison, we calculated a CNV score for each cell, defined as the average absolute deviation from reference‐normalized expression across the genome. Cells with elevated CNV scores were indicative of large‐scale genomic instability.

We further performed dimensionality reduction and graph‐based clustering based on CNV profiles, including PCA, neighborhood graph construction, and Leiden clustering. Clusters exhibiting high average CNV scores and predominantly originating from individual or limited numbers of tumor‐bearing samples—rather than being distributed broadly across donors—were classified as malignant. In contrast, clusters with low CNV scores and broad sample representation were considered non‐malignant.

### 6.7 Assessment of Developmental Potential and Stemness

To quantify the differentiation continuum and transcriptional plasticity of epithelial subpopulations, we employed CytoTRACE2 (v1.1.0), a state‐of‐the‐art deep learning framework for inferring absolute developmental potential from single‐cell transcriptomic data [56]. CytoTRACE2 outputs a continuous potency score ranging from 0 (terminally differentiated) to 1 (totipotent), while simultaneously categorizing each cell into one of six biologically defined potency states: Differentiated, Unipotent, Oligopotent, Multipotent, Pluripotent, and Totipotent. The underlying model was trained and validated on 34 scRNA‐seq datasets spanning 24 tissue types from both human and mouse, enabling robust cross‐dataset comparison of cellular ontogeny. To further assess the stem‐like characteristics of malignant epithelial cells, we applied the mRNA expression‐based stemness index (mRNAsi) developed by Malta et al. [57]. This index was derived using a one‐class logistic regression (OCLR) model trained on pluripotent stem cell samples (ESCs and iPSCs) from the Progenitor Cell Biology Consortium. The mRNAsi score for each cell was computed as the Spearman correlation between its gene expression profile and the OCLR‐derived weight vector, a method shown to be robust to inter‐dataset batch effects. Scores were then linearly scaled to the [0, 1] interval, with higher values reflecting stronger transcriptional stemness. This approach enabled high‐resolution quantification of stemness potential at the single‐cell level within the tumor microenvironment.

### 6.8 Trajectory Inference and Pseudotime Analysis

To reconstruct lineage trajectories of epithelial subpopulations, we performed Monocle 2 trajectory analysis [58]. Genes were detected using a minimum expression threshold of 0.1, followed by size‐factor normalization and dispersion estimation. Up to 3000 ordering genes with a mean expression level greater than 0.1 were selected for trajectory inference. Dimensionality reduction and principal‐graph learning were performed using discriminative dimensionality reduction with trees, with two reduced dimensions, logarithmic normalization, and the accelerated implementation. Cells were subsequently ordered along the learned principal tree to calculate pseudotime and assign trajectory states.

### 6.9 Consensus Non‐negative Matrix Factorization (cNMF)

To infer gene expression programs (GEPs) in malignant epithelial cells, we applied cNMF, a matrix factorization–based approach for scRNA‐Seq data [59]. cNMF decomposes the cell‐by‐gene expression matrix (N × G) into two non‐negative matrices: a program‐by‐gene matrix (K × G), representing GEPs, and a cell‐by‐program usage matrix (N × K), reflecting the contribution of each program to individual cells. The raw count matrix of malignant epithelial cells from EBV‐negative and EBV‐positive samples was used as input, and 2000 highly variable genes were selected. For each value of K from 6 to 15, 100 independent NMF factorizations were performed. The optimal number of programs was determined by jointly examining solution stability and reconstruction error in the K‐selection plot, resulting in the selection of K = 10. Before consensus clustering, unstable factorization spectra with local‐density values ≥ 0.2, defined according to their mean distances to nearest‐neighbor spectra, were excluded as outliers. The remaining spectra were aggregated to generate the final consensus programs and corresponding normalized program‐usage scores for individual cells.

### 6.10 Pseudo‐bulk Differential Expression and Enrichment Analysis

To compare gene expression between EBV^+^ and EBV^−^ malignant epithelial cells while preserving biological replication, raw transcript counts from tumor epithelial cells were aggregated by biological sample using the decoupleR framework with sum aggregation [60]. This strategy mitigates the pseudo‐replication bias inherent in single‐cell analyses by treating biological replicates, rather than individual cells, as independent units of observation, thereby improving statistical power and better capturing inter‐sample variability. Pseudo‐bulk profiles with insufficient cell numbers or transcript coverage were excluded during quality control. Differential expression analysis was performed using PyDESeq2 [61]. EBV status and clinical stage were included in the statistical design, with EBV^−^ samples serving as the reference group. PyDESeq2 estimated size factors and gene‐wise dispersions, fitted negative binomial generalized linear models, refitted genes identified as Cook's‐distance outliers, and performed Wald tests for the EBV^+^ vs. EBV^−^ contrast. Raw and Benjamini–Hochberg‐adjusted *P* values were calculated. In the code‐generated upregulated‐gene list, genes were selected using a nominal *P* < 0.05 and a positive log_2_ fold change. For enrichment analysis, the complete vector of gene‐level Wald statistics from the EBV^+^ vs. EBV^−^ comparison was used as input to decoupleR. Differential TF activity was inferred using the Univariate Linear Model with signed TF–target interactions from CollecTRI [62]. Differential pathway activity was likewise estimated using Univariate Linear Model with weighted pathway–target relationships from PROGENy [63]. Positive scores indicate relatively greater TF or pathway activity in EBV^+^ tumors, whereas negative scores indicate relatively greater activity in EBV^−^ tumors. In addition, to further contextualize the transcriptional differences, we performed functional enrichment analysis using curated gene sets from the MSigDB Hallmark collection and previously defined stemness‐, myeloid immunity‐, and immune function–related gene sets [64, 65].

### 6.11 Single‐Cell Cell–Cell Communication Analysis

Cell–cell communication was analyzed using CellChat in R [66]. Condition‐specific CellChat objects generated from the annotated scRNA‐seq data of EBV^−^ and EBV^+^ NPC were merged for comparative analysis, with the two datasets designated as Negative and Positive, respectively. Overall communication activity was compared between the two conditions using both the total number of inferred interactions and the aggregate interaction strength. The outgoing and incoming interaction strengths of individual cell populations were quantified to characterize their relative roles as signal senders and receivers. Differences in communication between individual sender–receiver cell pairs were examined using differential interaction networks and heatmaps. Signaling pathways were ranked according to differences in the number of inferred interactions. Statistical significance was evaluated using the CellChat‐implemented paired Wilcoxon test, and pathways with a nominal *P* < 0.05 were retained. Pathway‐specific networks were further visualized for MIF signaling from tumor cells to macrophage populations and SPP1 signaling from SPP1^+^ macrophages to tumor cells and terminally exhausted CD8^+^ T cells. At the ligand–receptor level, communication probabilities were compared between tumor cells and SPP1^+^ macrophages in both directions and between SPP1^+^ macrophages and terminally exhausted CD8^+^ T cells.

### 6.12 Calculation of Functional Module Scores, Correlation, and Survival Analyses

To validate transcriptional programs in external cohorts, we calculated gene set activity scores in bulk RNA‐seq datasets using the single‐sample gene set enrichment analysis (ssGSEA) method implemented in the GSVA package. Gene sets included stemness‐ and exhaustion‐related programs curated from previous studies, [53, 65] as well as top marker gene signatures derived from the single‐cell analysis. Expression matrices were log‐transformed and quantile‐normalized prior to scoring. To explore biological associations, Spearman correlation analysis was performed between module scores and key immune‐related signatures. Unless otherwise specified, patients were stratified into high‐ and low‐score groups using the median score as the cutoff. Progression‐free survival was evaluated using Kaplan–Meier curves and log‐rank tests. Multivariable Cox proportional hazards regression was performed with adjustment for clinical stage and histological subtype.

### 6.13 ST Data Processing and Analysis

The procedures for sample processing and data generation using the Visium Spatial for FFPE Gene Expression Kit (10X Genomics, USA) have been described in previous studies [47]. Spatial gene expression matrices processed with the Space Ranger software (v1.3.1, 10X Genomics) were imported into OmicVerse framework for downstream analyses. Twelve EBV^+^ NPC spatial transcriptomic samples were included. For each sample, only in‐tissue spots with total UMI counts greater than 500 and less than 60,000 and more than 100 detected genes were retained. A radius‐based spatial neighbor graph was constructed separately for each sample, targeting an average of 5–10 neighbors per spot. Candidate radius cutoffs of 200, 300, and 400 were evaluated through sensitivity analyses of graph density, cluster separation, inter‐sample mixing, spatial continuity, and niche stability. Gene‐expression counts were normalized to 10,000 counts per spot and log‐transformed using log1p, after which 3,000 highly variable genes were selected while accounting for the sample of origin. STAligner, a graph‐based deep‐learning method for spatial data integration, was used to jointly correct inter‐sample batch effects and learn spatially informed latent representations across tissue sections [67]. Model training was performed using 100 nearest neighbors and 600 epochs. Leiden clustering was subsequently performed on the STAligner embedding at resolutions of 0.1, 0.2, 0.4, 0.6, 0.8, and 1.0. The clustering resolution was selected based on clustering‐tree visualization and the combined evaluation of the Silhouette score, Calinski–Harabasz index, and Davies–Bouldin index. The clustering results were visualized using UMAP and mapped back onto the corresponding tissue coordinates for downstream analyses.

### 6.14 Spatial Deconvolution and Niche‐Level Cell‐Type Enrichment Analysis

To resolve the spatial distribution of distinct cell populations, we applied Tangram, a deep learning–based probabilistic framework that integrates scRNA‐seq with spatial transcriptomics data to infer spatially resolved cell‐type maps [68]. Up to 5,000 cells were randomly sampled per annotated subtype, with all cells retained for less abundant subtypes. After normalization to 10,000 counts, 2,000 highly variable genes and 2,000 spatially variable genes were selected from the single‐cell and spatial datasets, respectively. Tangram was run in cells mode with an RNA‐count–based density prior for 500 epochs. Predicted scores were constrained to non‐negative values and normalized within each spot to obtain cell‐type proportions; the cell type with the highest proportion was assigned as the dominant population for visualization. For niche‐level enrichment analysis, median cell‐type proportions were calculated for each sample–niche combination and then summarized across samples to generate a niche‐by‐cell‐type matrix. Hierarchical clustering was performed using Euclidean distance and complete linkage, with values standardized by cell type for visualization. Enrichment was assessed using one‐sided Wilcoxon rank‐sum tests comparing each niche with all other niches, followed by Benjamini–Hochberg correction.

### 6.15 Spatially Resolved Functional Inference and Niche‐Level Enrichment

To infer spatially resolved regulatory and functional states, we applied decoupleR to normalized and log‐transformed gene‐expression profiles at the individual‐spot level. The Univariate Linear Model was used throughout the analysis. Transcription factor activities were inferred using signed TF–target interactions from CollecTRI, whereas pathway activities were estimated using weighted pathway–target relationships from PROGENy. ULM‐based enrichment scores were additionally calculated for MSigDB Hallmark pathways. The resulting activity scores were mapped onto tissue coordinates and summarized across spatial niches. Niche‐associated regulators and pathways were ranked using one‐vs.‐rest t‐tests with overestimated variance, retaining features with positive test statistics; false‐discovery rate–adjusted *P* values were also calculated. Niche‐wise median activity scores were visualized using feature‐wise scaled heatmaps, with hierarchical clustering applied where appropriate.

### 6.16 Spatial Cell–Cell Communication Analysis

Spatially constrained communication analysis was performed using SpatialCellChat (v0.1.0) across 12 EBV^+^ NPC Visium samples. Spatial expression matrices were normalized using the LogNormalize method with a scale factor of 10,000. Tangram‐derived cell‐type proportions were aggregated into major communication groups and used as a cell‐type decomposition matrix for group‐level signaling inference; each spot was additionally labeled by its dominant cell type. Spots belonging to responder‐enriched Niche4, Niche6, and Niche8 were compared with those belonging to non‐responder‐enriched Niche5 and Niche7, whereas the remaining niches were excluded. Tissue coordinates were converted from pixels to micrometers, and individual tissue sections were spatially separated to prevent artificial cross‐sample interactions. Separate SpatialCellChat objects were constructed for the two niche groups using the Secreted Signaling database. Spatially variable signaling genes were identified using MERINGUE, and distance‐constrained communication probabilities were estimated using an interaction range of 500 µm and a Visium contact range of 100 µm. Tangram decomposition–weighted group‐level probabilities were calculated using average aggregation and 100 permutations. Communications supported by fewer than 10 spatial links or fewer than 10 sending or receiving spots were filtered out. The two objects were then compared with respect to interaction number and strength, incoming and outgoing signaling roles, and pathway‐specific communication patterns.

### 6.17 Multiplex Immunofluorescence Staining and Analysis

Multiplex immunofluorescence staining was performed on formalin‐fixed, paraffin‐embedded tumor specimens from one EBV^−^ and one EBV^+^ NPC case using the PANO 7‐plex immunohistochemistr kit (Panovue, cat. no. 0004100100) according to the manufacturer's instructions. Sections (5 µm thick) were deparaffinized, rehydrated, and subjected to antigen retrieval. Sequential staining was performed using primary antibodies against EPCAM, MIF, SOX2, SPP1, and CD206, followed by horseradish peroxidase‐conjugated secondary antibodies and tyramide signal amplification. After each staining cycle, microwave‐mediated antibody stripping was performed before application of the next primary antibody. Following completion of all staining cycles, nuclei were counterstained with 4′,6‐diamidino‐2‐phenylindole (DAPI). Whole‐slide fluorescence images were acquired using an Olympus VS200 slide scanner and analyzed using QuPath. Cells were identified by nuclear segmentation based on DAPI staining, and marker‐positive populations were classified using consistently applied intensity thresholds. Tumor and stromal compartments were delineated based on tissue morphology and EPCAM staining and were manually reviewed. The densities of CD206^+^, CD206^+^SPP1^+^, EPCAM^+^, EPCAM^+^MIF^+^, EPCAM^+^SOX2^+^, and EPCAM^+^MIF^+^SOX2^+^ cells were calculated as the number of positive cells per square millimeter within the total tissue, tumor, and stromal compartments.

### 6.18 Cell lines and EBV Infection

We utilized the normal epithelial cell line NP69 (Abiowell, AW‐CNH626), the EBV^+^ NPC cell line C666‐1 (Abiowell, AW‐CCH168), and the EBV^−^ NPC cell line HK1 (Abiowell, AW‐CCH310). Two additional EBV^+^ cell lines (NP69‐EBV and HK1‐EBV) were generated by infecting their parental cells with recombinant EBV carrying an EGFP reporter, produced from Akata‐EBV‐GFP cells (HyCyte, TCH‐C588G) as previously described [69, 70]. Stable EBV‐infected clones were selected with 0.5 µg/mL Puromycin (Gibco, A1113803) and verified by EGFP fluorescence (Leica DMI8) and qPCR analysis of the EBV latency‐ and lytic‐cycle genes. NP69 and NP69‐EBV cells were maintained in NP69‐specific complete medium (Abiowell, AW‐MNH626) composed of Keratinocyte Serum‐Free Medium (KSFM) supplemented with 1% Keratinocyte Growth Supplement, 2% premium‐grade FBS, 0.2 ng/mL EGF, and 1% penicillin‐streptomycin (P/S). In contrast, HK1, HK1‐EBV, C666‐1, and Akata‐EBV‐GFP cells were cultured in RPMI‐1640 (Gibco, C11875500BT) supplemented with 10% FBS and 1% P/S. All cell lines were maintained at 37°C in a humidified atmosphere containing 5% CO_2_. Cell identity was authenticated by short tandem repeat profiling, and cultures were routinely screened for mycoplasma contamination using a PCR detection kit (Beyotime, C0301S).

### 6.19 Cell Culture and Treatment

The human monocytic leukemia cell line THP‐1 (Abiowell, AW‐CCH098) was cultured in RPMI‐1640 medium supplemented with 10% premium‐grade FBS, 1% penicillin‐streptomycin, and 0.05 mM β‐mercaptoethanol at 37°C in a humidified 5% CO_2_ atmosphere. To induce differentiation into M0 macrophages, THP‐1 cells were treated with 100 ng/mL phorbol 12‐myristate 13‐acetate (PMA; MCE, HY‐18739) for 24 h. Subsequently, the PMA‐containing medium was removed, and cells were cultured in fresh PMA‐free medium for an additional 24 h to allow recovery before subsequent experiments. For generation of primary human monocyte‐derived macrophages (MDMs), CD14^+^ monocytes isolated from human peripheral blood mononuclear cells (PBMCs) were cultured in RPMI‐1640 supplemented with 10% FBS and macrophage colony‐stimulating factor (M‐CSF, 50 ng/mL) for 7 days. THP‐1‐derived M0 macrophages and M‐CSF‐differentiated primary MDMs were treated with CM collected from HK‐1, HK‐1‐EBV, NP69, NP69‐EBV, or C666‐1 cells. To investigate the role of MIF, recombinant human MIF (rhMIF, 50ng/ml) was added to macrophages. For MIF inhibition experiments, macrophages were pretreated with the MIF inhibitor MIF098 (10 µM) for 1 h before treatment with the corresponding NPC‐derived CM.

### 6.20 Multiplex Cytokine Analysis

Cell culture supernatants from HK‐1, NP69, EBV‐HK‐1, EBV‐NP69, and C666‐1 cells were collected and centrifuged twice at 300 g for 10 min at 4°C to remove cellular debris. The supernatants were aliquoted and stored at ‐80°C for analysis. A panel of 46 cytokines was quantified using a Luminex‐based multiplex assay kit (RnD, LKT014B) according to the manufacturer's instructions. Briefly, samples, standards, and quality controls were incubated with antibody‐conjugated magnetic beads, followed by sequential incubation with detection antibodies and phycoerythrin (PE)‐conjugated streptavidin. Beads were washed between incubation steps, and fluorescence signals were measured using a Luminex‐200 system (Luminex). Cytokine concentrations were calculated from the corresponding standard curves.

### 6.21 ELISA

MIF and SPP1 concentrations in cell culture supernatants were measured using ELISA kits according to the manufacturer's instructions. Briefly, MIF concentrations in the culture supernatants of HK‐1, NP69, HK‐1‐EBV, NP69‐EBV and C666‐1 cells were determined using a human MIF ELISA kit (Absin, abs5510114). SPP1 concentrations in the culture supernatants of THP‐1‐derived M0 macrophages and M‐CSF‐differentiated primary human MDMs were measured using a human SPP1 ELISA kit (Absin, abs551913) following treatment with CM collected from HK‐1, HK‐1‐EBV, NP69, NP69‐EBV, or C666‐1 cells.

### 6.22 Magnetic Separation of CD8^+^ T cells and CD14^+^ Monocytes

PBMCs were washed with PBS and resuspended in separation buffer. Cells were adjusted to approximately 10^7^ cells per tube and incubated with human CD8 Nanobeads (STARTED, S0K0010) at 4°C for 15 min in the dark. After washing with separation buffer, the labeled cells were loaded onto L Separation Columns placed in a magnetic field. The columns were washed according to the manufacturer's instructions. CD8^−^ cells were collected from the flow‐through fraction, whereas CD8^+^ T cells were eluted after removal of the columns from the magnetic field. The collected CD8^−^ cells were subsequently subjected to magnetic separation using human CD14 Nanobeads (STARTED, S0K0004) under the same conditions. CD14^+^ monocytes were collected from the magnetic fraction after column removal and elution. The isolated CD8^+^ T cells and CD14^+^ monocytes were collected and used for subsequent experiments.

### 6.23 CD8^+^ T cell Activation and Treatment

Purified CD8^+^ T cells were seeded into 24‐well plates and activated with anti‐CD3 (5µg/ml), anti‐CD28 (5µg/ml) and recombinant human IL‐2 (10 ng/mL) for 24 h. Following activation, the medium containing anti‐CD3 and anti‐CD28 was removed, and the cells were treated with macrophage‐derived CM mixed with fresh T‐cell culture medium at a 1:1 ratio. For CD44 blockade experiments, CD8^+^T cells were pretreated with an anti‐CD44 blocking antibody (10 µg/ml) for 1 h before treatment with the corresponding macrophage‐derived CM or rhSPP1. Cells were subsequently cultured at 37°C for an additional 48 h before flow cytometric analysis.

For CFSE labeling, before seeding into 24‐well plates, the purified CD8^+^T cells were resuspended in serum‐free PBS and labeled with carboxyfluorescein succinimidyl ester (CFSE, 5 µM) at 37°C for 10 min in the dark. The staining reaction was subsequently quenched by incubation on ice for 5 min. Cells were washed with PBS, resuspended in complete culture medium, and seeded for subsequent activation and CM treatment.

### 6.24 Flow Cytometry

Cell surface molecule staining was performed at 4°C for 30 min in PBS in the dark. For intracellular staining, CD8^+^T cells were fixed and permeabilized using an intracellular fixation and permeability kit (invitrogen, 88‐8824‐00) followed by antibody staining according to manufacturer's instructions. After three washes with cold PBS, cells were resuspended in PBS and acquired on an LSRFortessa X‐20 flow cytometer. Data were analyzed using FlowJo v10. The antibodies used in this study are Fixable Viability Dye 777 (STARTER, S0D0026), BB515 anti‐human CD3 (BD Pharmingen, 564465), BUV395 anti‐human CD8 (BD Pharmingen, 563796), BV711 anti‐human PD‐1 (BD Pharmingen, 740814), RY775 anti‐human LAG‐3 (BD Pharmingen, 771755), BV421 anti‐human TIM3 (BD Pharmingen, 747962), RB705 anti‐human IFN‐γ (BD Pharmingen, 570247), PE‐CF594 anti‐human TNF (BD Pharmingen, 562784), PE anti‐human GZMB (BD Pharmingen, 561142), AF647 anti‐human Perforin (BD Pharmingen, 563576) and R718 anti‐human IL‐2 (BD Pharmingen, 567057).

### 6.25 Western Blotting

Cells were washed twice with ice‐cold PBS and lysed in Radio‐Immunoprecipitation Assay (RIPA) Lysis buffer (Strong) (CWBIO, CW2333S) supplemented with a cocktail of protease inhibitors (CWBIO, CW2200S). Lysates were clarified, and protein concentration was determined using a bicinchoninic acid (BCA) kit (Absin, abs9232). Equal amounts of protein were mixed with 5× SDS sample loading buffer to a final concentration of 1× and denatured by heating at 100°C for 10 min, separated by SDS‐PAGE, and transferred onto polyvinylidene difluoride (PVDF) membranes (Merck millipore, IPVH00010) pre‐activated in methanol. Membranes were blocked with 5% nonfat milk in TBST and incubated with primary antibodies overnight at 4°C, followed by incubation with HRP‐conjugated secondary antibodies for 2 h at room temperature. Signals were developed with ECL (NCM Biotech, P10300) and captured using a ChemiDoc XRS+System (BIORAD). Densitometry was performed in ImageJ 1.53t and target proteins were normalized to GAPDH. The primary antibodies used are anti‐SPP1 (Proteintech, 22952‐1‐AP), anti‐CD163 (CST, 93498T), anti‐CD206 (CST, 24595T), anti‐IL‐10 (CST, 12163T), anti‐TGF‐β (CST, 3709T), anti‐MIF (CST, 87501T), anti‐SOX2 (CST, 3579T), anti‐NANOG (CST, 4903T), anti‐OCT‐4 (CST, 75463T), anti‐CD44 (CST, 37259T) and anti‐GAPDH (Proteintech, 60004‐1‐Ig).

### 6.26 RNA Extraction and Quantitative Real‐Time PCR

Cells were lysed directly in culture wells with Buffer RL and total RNA was purified using the FastPure Cell/Tissue TotalRNA Isolation Kit V2 (Vazyme, RC112‐01) according to the manufacturer's protocol, including on‐column gDNA removal. RNA concentration and purity were assessed on a Nanodrop one system (Thermo Scientific) and reverse transcribed with a synthesis kit (Takara, RR036A). qRT‐PCR was performed with ChamQ Universal SYBR qPCR Master Mix (Vazyme, Q711‐02) on a CFX96 Touch instrument (Bio‐Rad, USA). Relative expression was calculated by the 2^−ΔΔCt^ method with β‐actin as the internal control.

### 6.27 Cell Viability Assay

Cell viability was assessed using the Cell Counting Kit‐8 (CCK‐8; Beyotime, C0038). NPC‐EBV cells in the logarithmic growth phase were seeded into 96‐well plates at a density of 2000 cells per well and incubated at 37°C in a humidified atmosphere with 5% CO_2_. After cell attachment, the culture medium was replaced with the corresponding CM collected from THP‐1‐derived macrophages or medium containing rhSPP1 (200 ng/ml). Following treatment for 24 h, 10 µL of CCK‐8 solution was added to each well and incubated for 2 h at 37°C. The absorbance at 450 nm was measured using a cytaion3 imaging reader.

### 6.28 Transwell Invasion Assay

NPC‐EBV cells were washed twice with serum‐free RPMI medium and resuspended at a density of 3× 10^4^ cells in 200 µL serum‐free RPMI medium. The cell suspension was seeded into Matrigel‐coated Transwell inserts with 8‐µm pores. The lower chambers were filled with 600 µL of corresponding CM collected from THP‐1‐derived macrophages or with rhSPP1 (200 ng/ml). After incubation for 24 h at 37°C, cells remaining on the upper surface of the membrane were gently removed with a cotton swab. Cells were fixed with 4% paraformaldehyde for 15 min and stained with 1% crystal violet for 15 min. After washing with PBS, the membranes were imaged using an inverted microscope. Three non‐overlapping fields were quantified for each technical replicate. Technical replicates were averaged to generate one value for each biological replicate before statistical analysis.

### 6.29 Primary Human Monocyte Transwell Migration Assay

Primary human peripheral blood monocytes were labeled with CellTracker Green CMFDA (Thermo Fisher Scientific, C2925) at 37°C for 30 min, washed, and resuspended in serum‐free RPMI 1640 medium at 5 × 10^5^ cells/mL. Conditioned media collected from parental NP69 and HK1 cells, their EBV‐infected derivatives, or C666‐1 cells were added to the lower chambers. MIF098 was added to the indicated CM conditions, whereas recombinant human MIF (rhMIF) diluted in serum‐free medium was used as a positive control. CMFDA‐labeled primary monocytes were added to the upper chambers of Transwell inserts. After incubation for 24 h at 37°C, migrated monocytes were imaged and quantified by fluorescence microscopy. Three non‐overlapping fields were quantified for each technical replicate. Technical replicates were averaged to generate one value for each biological replicate before statistical analysis.

### 6.30 Tumor Sphere Formation Assay

HK‐1‐EBV and C666‐1 cells were dissociated into single‐cell suspensions and seeded into ultra‐low attachment plates at densities of 3,000 cells per well, respectively. Cells were cultured in serum‐free DMEM/F12 medium supplemented with B27 (1×), epidermal growth factor (EGF, 20 ng/mL), basic fibroblast growth factor (FGF, 20 ng/mL), and penicillin/streptomycin (1×). To investigate the effect of macrophage‐derived factors on tumor sphere formation, conditioned media collected from THP‐1‐derived macrophages with different SPP1 expression levels, including NC‐sh‐SPP1, sh‐SPP1, NC‐OE‐SPP1 and OE‐SPP1 macrophages, were mixed with fresh sphere‐forming medium at a 1:1 ratio and added to the tumor cells. For recombinant SPP1 treatment, rhSPP1 (200 ng/ml) was added to the sphere‐forming medium. The culture medium was replenished every 3 days using the corresponding treatment medium. After approximately 7 days of culture, tumor sphere formation was evaluated by phase‐contrast microscopy, and representative images were captured.

### 6.31 Statistical Analysis

All statistical analyses were performed using R (version 4.5.0) or Python (version 3.9.5). The differences between groups were analyzed by Fisher's exact test, Chi‐square test, Student's t‐test, Wilcoxon rank‐sum test, Kruskal‐Wallis test, and Spearman correlation test, when appropriate. All tests were two‐sided and *P* < 0.05 was considered as statistical significance. Unless otherwise indicated, quantitative data are presented as mean ± SD from three independent biological replicates.

## Author Contributions

W.Z.L. and Y.Q.X. conceived the study and supervised the project. G.Y.L., S.H.L., S.Q.H., and Y.F.J. carried out patient recruitment, data acquisition, and experimental analysis, and drafted the original manuscript. G.Y.L., W.Z.L., and Y.Q.X. acquired the funding. All authors contributed to data interpretation, critically revised the manuscript, and approved the final version for submission.

## Ethics Statement

This study represents an exploratory analysis derived from our previously published clinical trial, which was approved by the Chinese Ethics Committee of Registering Clinical Trials (registration number: ChiCTR2000032317).

## Conflicts of Interest

The authors declare no conflicts of interest.

## Supporting information




**Supporting File**: advs78450‐sup‐0001‐SuppMat.pdf.

## Data Availability

The raw and processed single‐cell RNA‐seq data generated in this study (trial registration: ChiCTR2000032317) have been deposited in Zenodo under record number 13736891. The previously published bulk RNA‐seq, single‐cell RNA‐seq, and spatial transcriptomics datasets from Sun Yat‐sen University Cancer Center used in this study are publicly available from the Gene Expression Omnibus (GEO; GSE162025 and GSE206245), the China National GeneBank DataBase (CNGBdb; CNP0000428), the Genome Sequence Archive for Human (GSA‐Human; HRA000087 and HRA003340), and the National Center for Biotechnology Information (NCBI) BioProject database (PRJNA633797 and PRJNA680328). All analytical codes are available on GitHub (https://github.com/Niubile001/EBV‐NPC‐Atlas) and will be updated to reflect the final version of the manuscript upon acceptance. Further data supporting the findings of this study are available in the Supporting Information or from the corresponding author upon reasonable request.
