# Supplementary material for: Integrated Multiomic Analysis Reveals an EBV‐Associated MIF–SPP1+ Macrophage Axis Linking Tumor Stemness and Immune Dysfunction in Nasopharyngeal Carcinoma
Source: Adv Sci (Weinh). 2026 Oct 31:e78450. Online ahead of print. doi: 10.1002/advs.78450 (PMC13723717; doi:10.1002/advs.78450)
Supplement: Supplementary file 1 [file ADVS-9999-e78450-s001.pdf]

Supplementary Materials

Supplementary Figures

Figure S1. Overview of the computational workflows used in this study. 1
Figure S2. Quality control of single-cell RNA sequencing data. 2
Figure S3. Benchmarking of batch correction and integration methods for the single-cell RNA-seq datasets. 3
Figure S4. Cluster-specific genes and canonical marker expression across single-cell clusters. 4
Figure S5. CNV-based identification of tumor and non-tumor epithelial compartments.. 5
Figure S6. Validation of EBV latency- and lytic-cycle-associated gene expression in EBV^+ epithelial cell models. 6
Figure S7. Identification and clinical evaluation of GEPs in malignant epithelial cells. 7
Figure S8. Myeloid sub-clustering and marker-based annotation reveal transcriptional heterogeneity in nasopharyngeal carcinoma. 8
Figure S9. Prognostic relevance of macrophage-related signatures in NPC. 9
Figure S10. Marker-based annotation of T/NK-cell subclusters. 10
Figure S11. Sensitivity analysis and parameter selection for spatial niche definition. 11
Figure S12. Spatial distribution of eight niches across EBV^+ NPC specimens. 12
Figure S13. Subsampled single-cell transcriptomic reference atlas used for Tangram deconvolution. 13
Figure S14. Tangram-inferred cell-type compositions of spatial transcriptomic spots across specimens. 14
Figure S15. Cell–cell communication analysis identifies EBV-associated remodeling of MIF and SPP1 signaling in EBV^+ NPC. 16
Figure S16. Association of MIF and the $SPP1^+$ macrophage signature with clinical outcome. 17
Figure S17. Representative Transwell images of primary monocyte migration following treatment with the indicated cell-derived CM, MIF098, or rhMIF. 18

Supplementary Tables

Table S1. Summary of NPC samples for scRNA-seq. 19
Table S2. Summary of NPC samples for spatial transcriptomics. 23
Table S3. Benchmarking of batch correction and integration methods for the single-cell RNA-seq datasets. 24
Table S4. Summary of NPC samples for bulk RNA-seq. 25
Table S5. Gene sets used in the study. 28
Table S6. Top-ranked genes of different gene expression programs identified by cNMF. 47

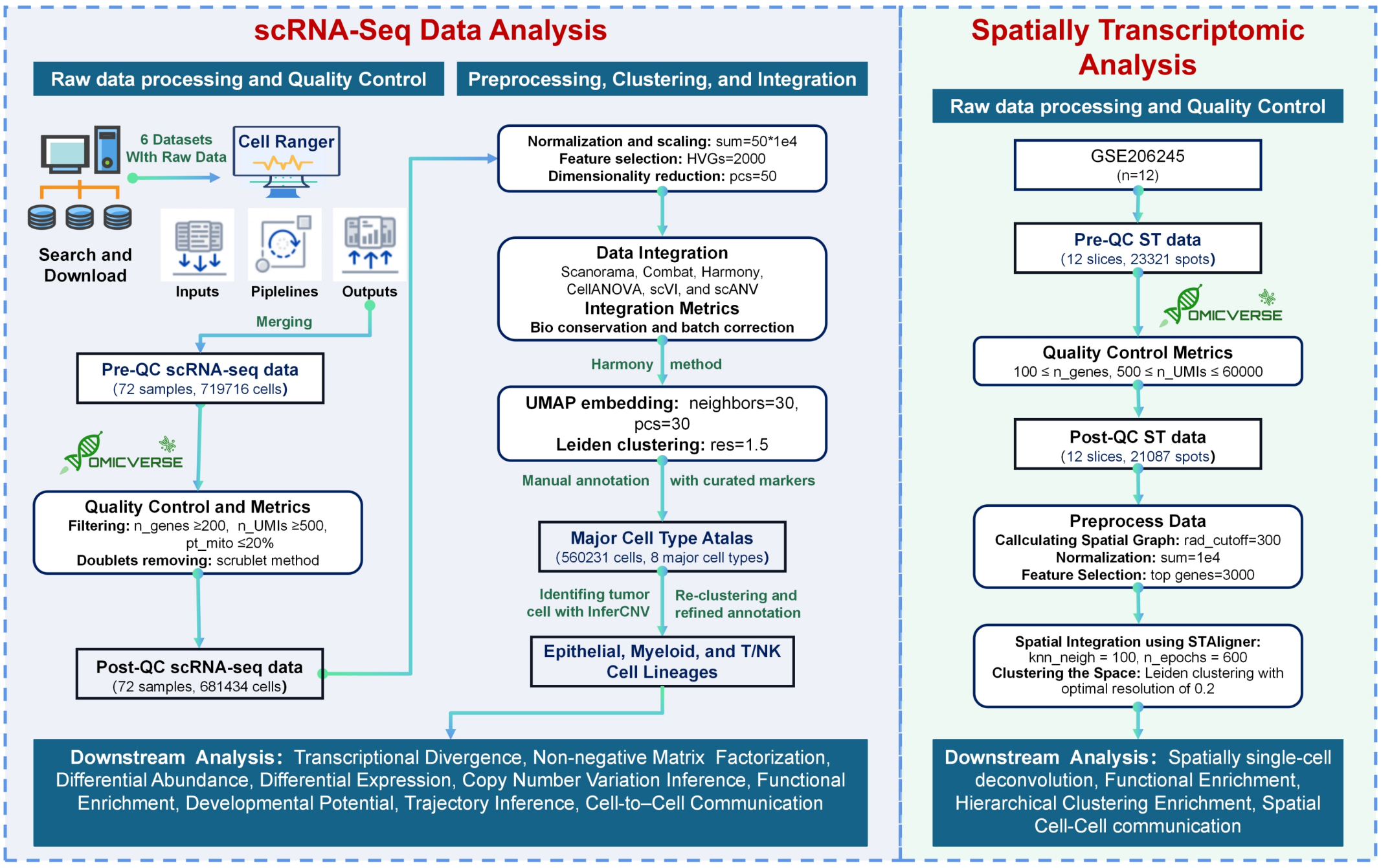

Figure S1. Overview of the computational workflows used in this study. Left, workflow for single-cell RNA-seq data processing and downstream analysis. Right, workflow for spatial transcriptomic data processing and downstream spatial analysis.

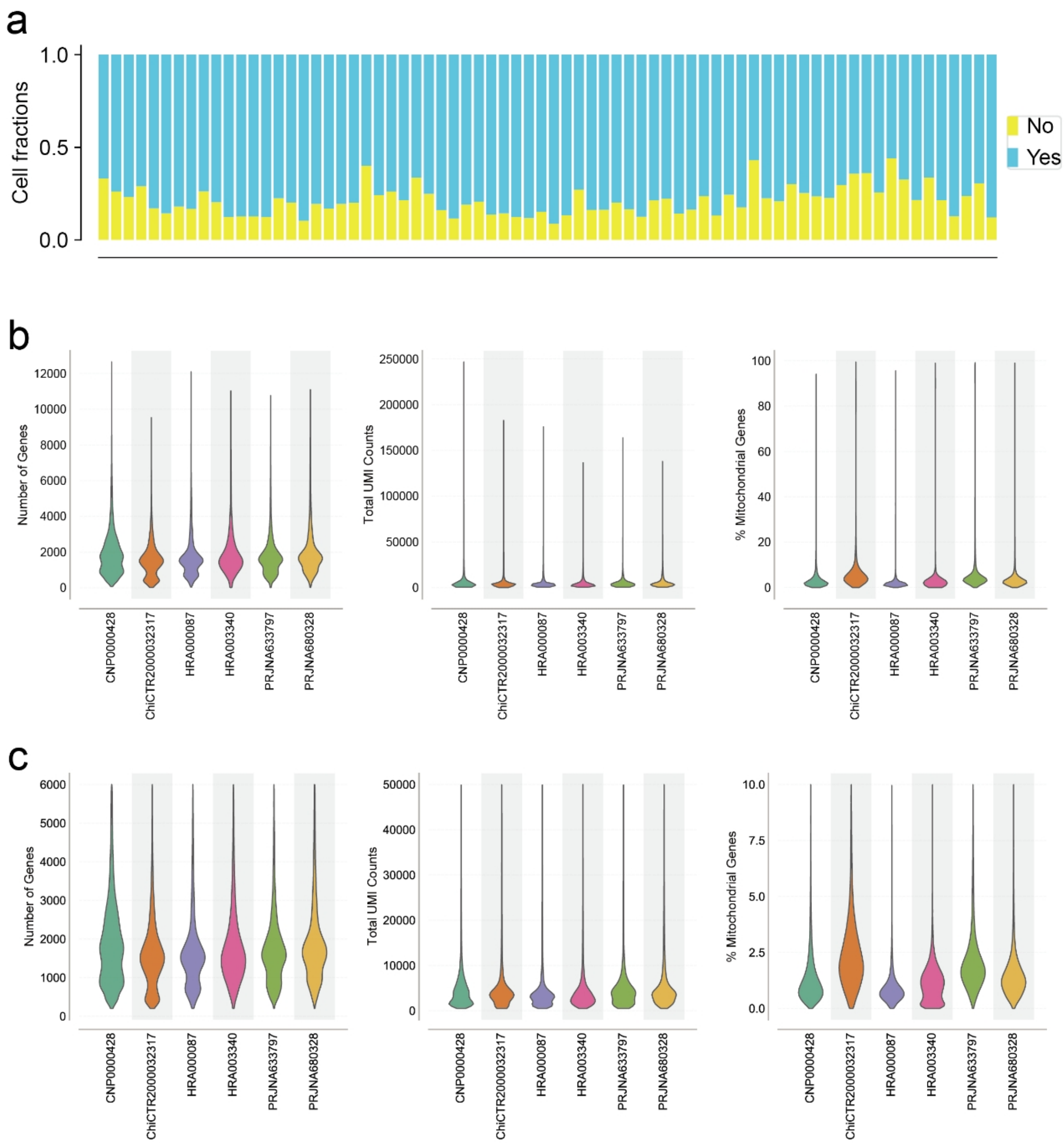

Figure S2. Quality control of single-cell RNA sequencing data. (a) Proportion of cells that passed versus failed quality control for each sample. (b) Distributions of per-cell quality metrics before filtering: number of detected genes per cell, total transcript counts per cell, and percentage of mitochondrial transcripts per cell. (c) The same metrics after quality filtering.

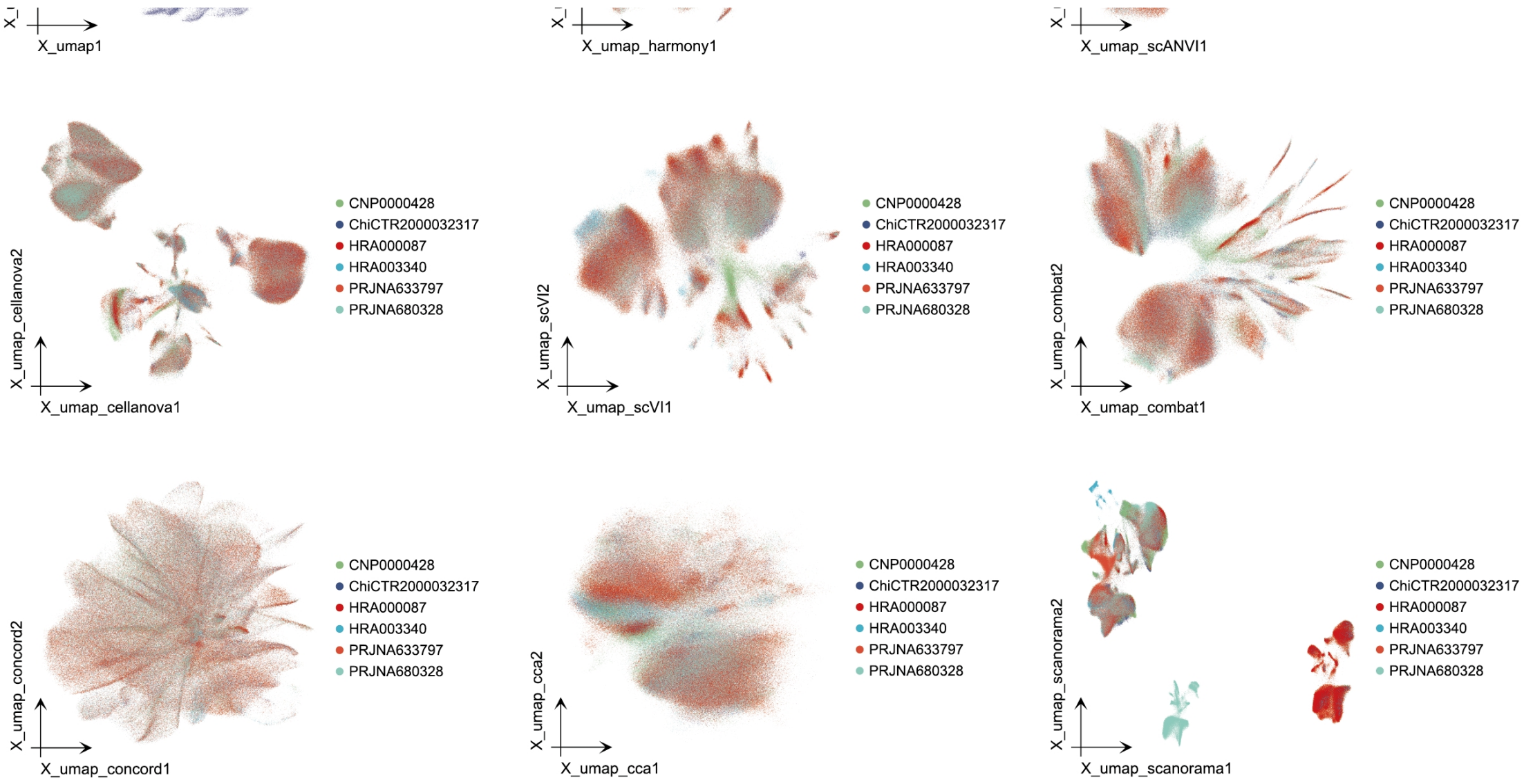

b

Method	Bio conservation					Batch correction					Aggregate score		
	Isolated labels	KMeans NMI	KMeans ARI	Silhouette label	cLISI	BRAS	iLISI	KBET	Graph connectivity	PCR comparison	Batch correction	Bio conservation	Total
X_harmony	0.65	0.69	0.51	0.65	1.00	0.87	0.08	0.31	0.92	0.65	0.57	0.70	0.65
X_cellanova	0.54	0.64	0.48	0.63	1.00	0.81	0.08	0.27	0.89	0.69	0.55	0.66	0.61
X_scANVI	0.62	0.70	0.51	0.66	1.00	0.74	0.06	0.21	0.97	0.43	0.48	0.70	0.61
X_combat	0.52	0.54	0.43	0.58	1.00	0.76	0.03	0.15	0.94	1.00	0.58	0.62	0.60
X_scVI	0.58	0.66	0.43	0.55	1.00	0.76	0.04	0.19	0.94	0.53	0.49	0.64	0.58
Unintegrated	0.57	0.56	0.40	0.60	1.00	0.56	0.01	0.17	0.95	0.00	0.34	0.62	0.51
X_concord	0.55	0.16	0.03	0.45	1.00	0.82	0.07	0.24	0.96	0.89	0.59	0.44	0.50
X_cca	0.45	0.21	0.15	0.48	0.90	0.76	0.04	0.21	0.38	0.99	0.48	0.44	0.45
X_scanorama	0.47	0.01	-0.00	0.47	0.81	0.71	0.04	0.08	0.20	0.00	0.21	0.35	0.29

c

Method	Bio conservation					Batch correction					Aggregate score		
	Isolated labels	KMeans NMI	KMeans ARI	Silhouette label	cLISI	BRAS	iLISI	KBET	Graph connectivity	PCR comparison	Batch correction	Bio conservation	Total
X_harmony	1.00	1.00	1.00	0.95	1.00	1.00	1.00	1.00	0.94	0.65	0.92	0.99	0.96
X_scANVI	0.84	1.00	1.00	1.00	1.00	0.59	0.67	0.56	1.00	0.44	0.65	0.97	0.84
X_cellanova	0.47	0.91	0.95	0.86	1.00	0.80	1.00	0.84	0.89	0.69	0.84	0.84	0.84

Figure S3. Benchmarking of batch correction and integration methods for the single-cell RNA-seq datasets. (a) UMAP embeddings of the unintegrated data and data integrated using eight methods: Harmony, cellANOVA, scANVI, ComBat, scVI, CONCORD, CCA, and Scanorama. Cells are colored according to their source dataset. (b) Quantitative comparison of the integration methods using individual biological conservation and batch-correction metrics, together with the corresponding aggregate batch-correction, biological conservation, and overall scores. Methods are ranked according to their overall integration score. UMAP, uniform manifold approximation and projection; NMI, normalized mutual information; ARI, adjusted Rand index; cLISI, cell-type local inverse Simpson's index; BRAS, batch removal adapted silhouette score; iLISI, integration local inverse Simpson's index; kBET, k-nearest-neighbor batch-effect test; PCR, principal component regression; CCA, canonical correlation analysis.

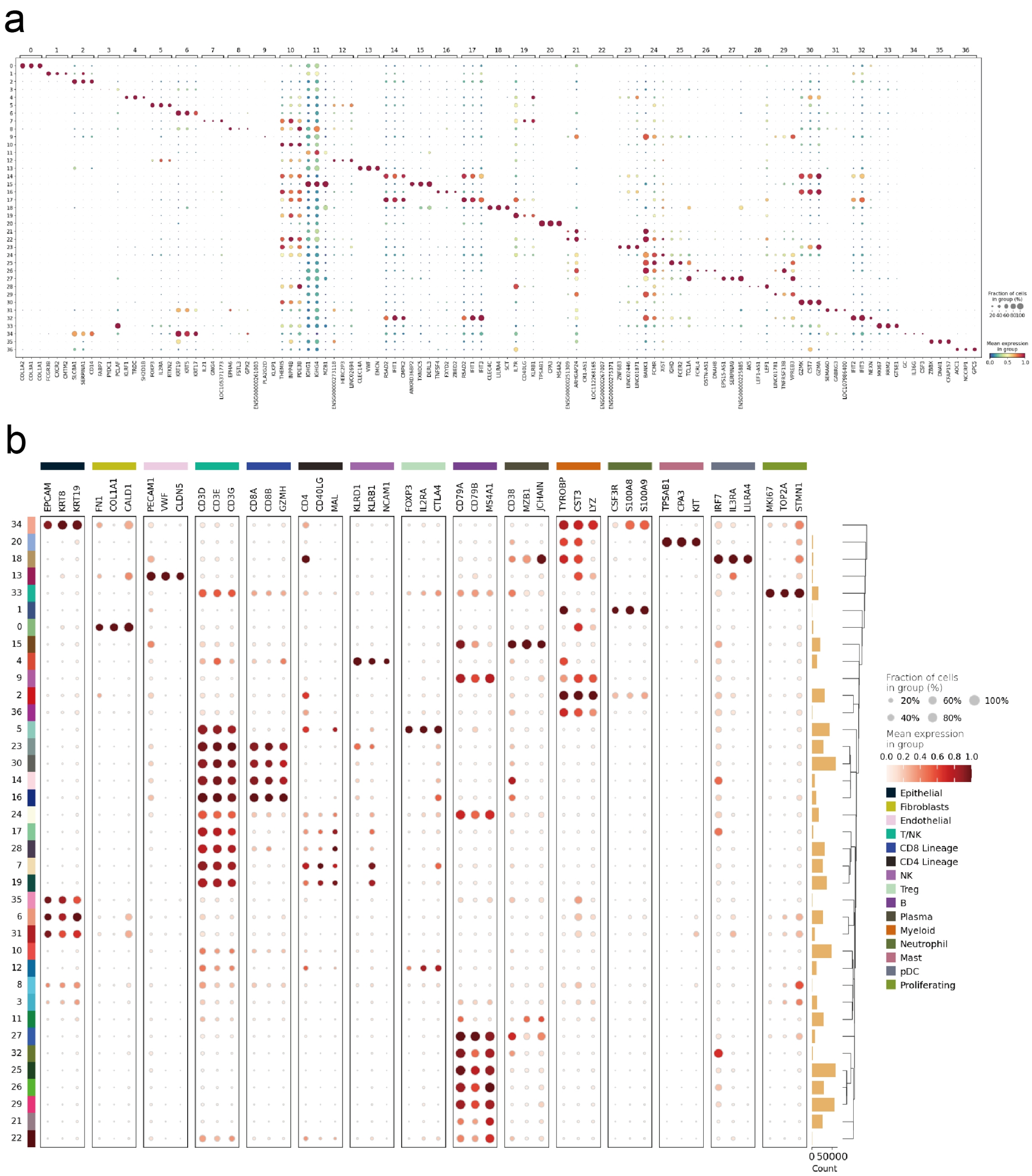

Figure S4. Cluster-specific genes and canonical marker expression across single-cell clusters. (a) Dot plot showing the top three cluster-enriched genes identified by one-versus-rest differential expression analysis for each cluster. Rows represent clusters, whereas columns represent genes grouped according to the cluster in which they were top-ranked. (b) Dot plot showing literature-curated canonical markers grouped by major cell lineage. Clusters are hierarchically ordered according to their marker-expression profiles, with the colored annotation bars indicating the assigned cell lineages. The dendrogram depicts the relationships among clusters, and the horizontal bars on the right indicate the number of cells in each cluster.

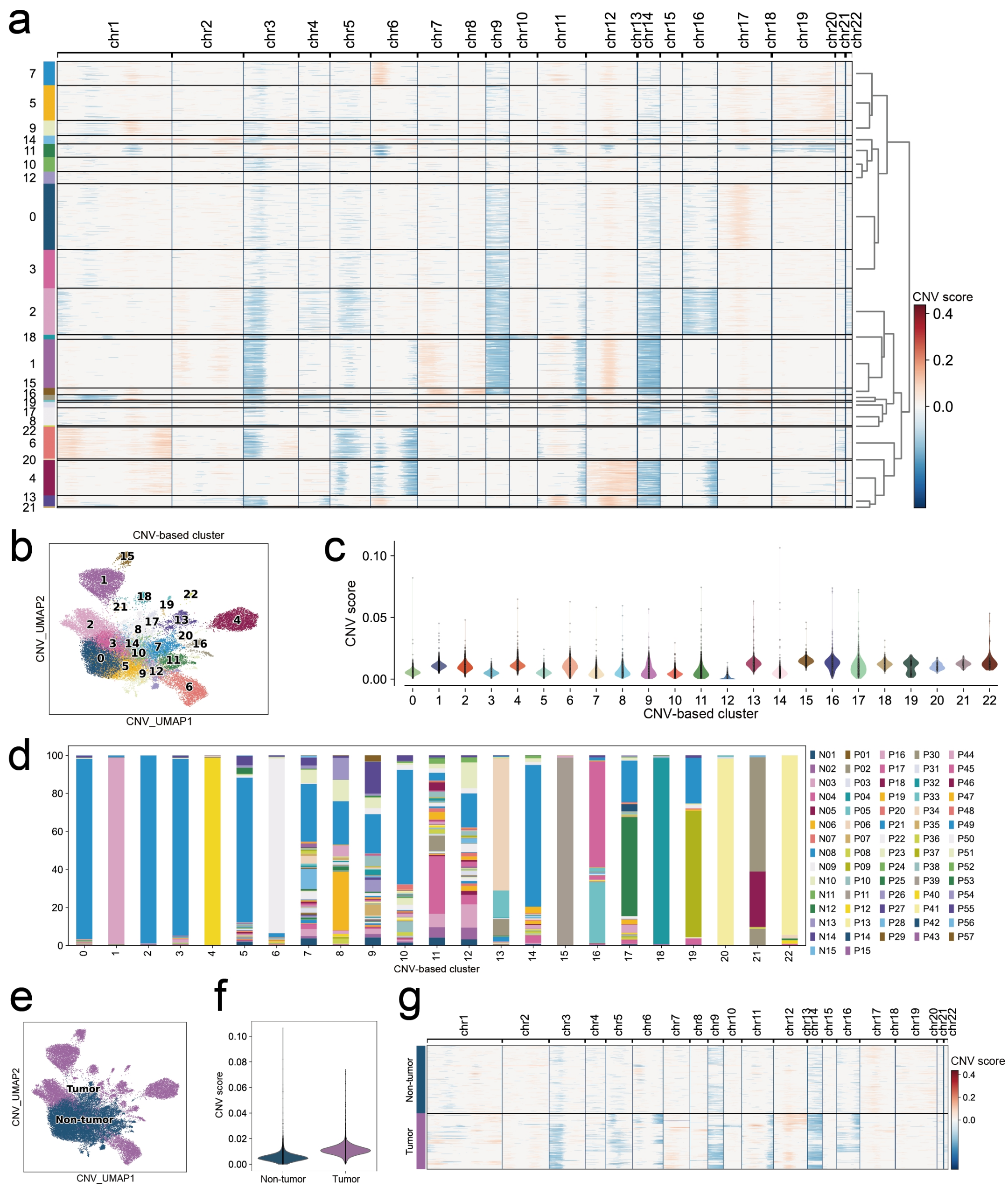

Figure S5. CNV-based identification of tumor and non-tumor epithelial compartments. (a) Heatmap of inferred chromosomal CNV profiles across CNV-based Leiden clusters. (b) UMAP embedding of epithelial cells in CNV space, colored by CNV-based cluster. (c) Violin plots showing the distribution of inferred CNV scores across clusters. (d) Sample composition of each CNV-based cluster, displayed as stacked bars representing the fraction of cells derived from each sample. (e) UMAP embedding showing the CNV-based classification of epithelial cells into tumor and non-tumor compartments. (f) Violin plots comparing CNV-score distributions between tumor and non-tumor epithelial cells. (g) Heatmap comparing inferred chromosomal CNV profiles between the tumor and non-tumor compartments. Red and blue indicate relative copy-number gains and losses, respectively. Abbreviations: CNV, copy-number variation; UMAP, uniform manifold approximation and projection.

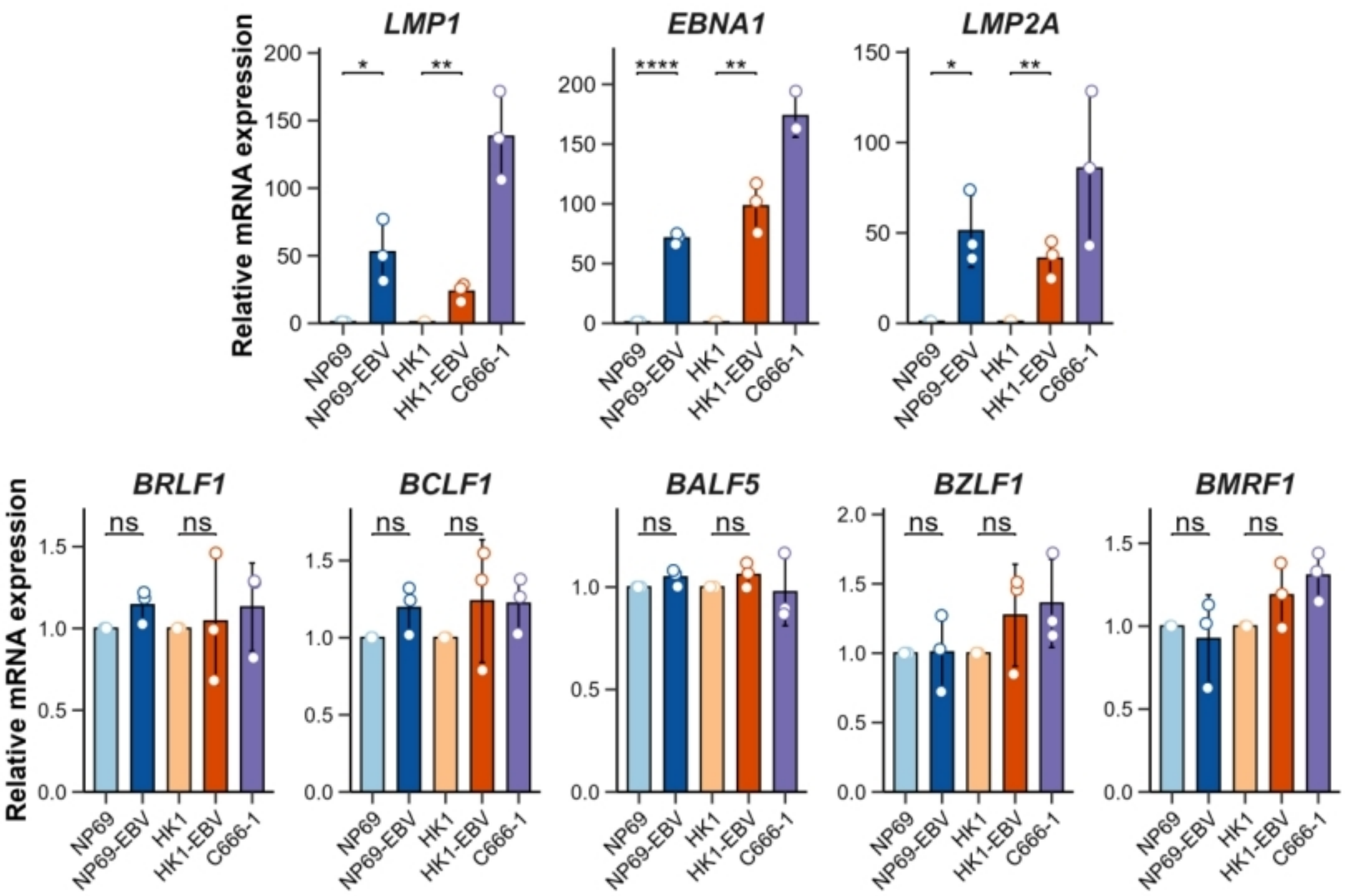

Figure S6. Validation of EBV latency- and lytic-cycle-associated gene expression in EBV⁺ epithelial cell models. qPCR analysis of EBV-associated transcripts in parental NP69 and HK1 cells, stable EBV⁺ NP69 and HK1 cells, and the EBV⁺ NPC cell line C666-1. Upper panels show latency-associated LMP1, EBNA1, and LMP2A; lower panels show representative immediate-early (BRLF1 and BZLF1), early (BALF5 and BMRF1), and late (BCLF1) lytic-cycle genes. Each dot represents one independent biological replicate, with three biological replicates performed per group ($n = 3$). Bars show mean ± SD. Statistical comparisons were performed using two-sided unpaired Student's t-test. ns, not significant; $^{*}P < 0.05$, $^{**}P < 0.01$, $^{****}P < 0.0001$.

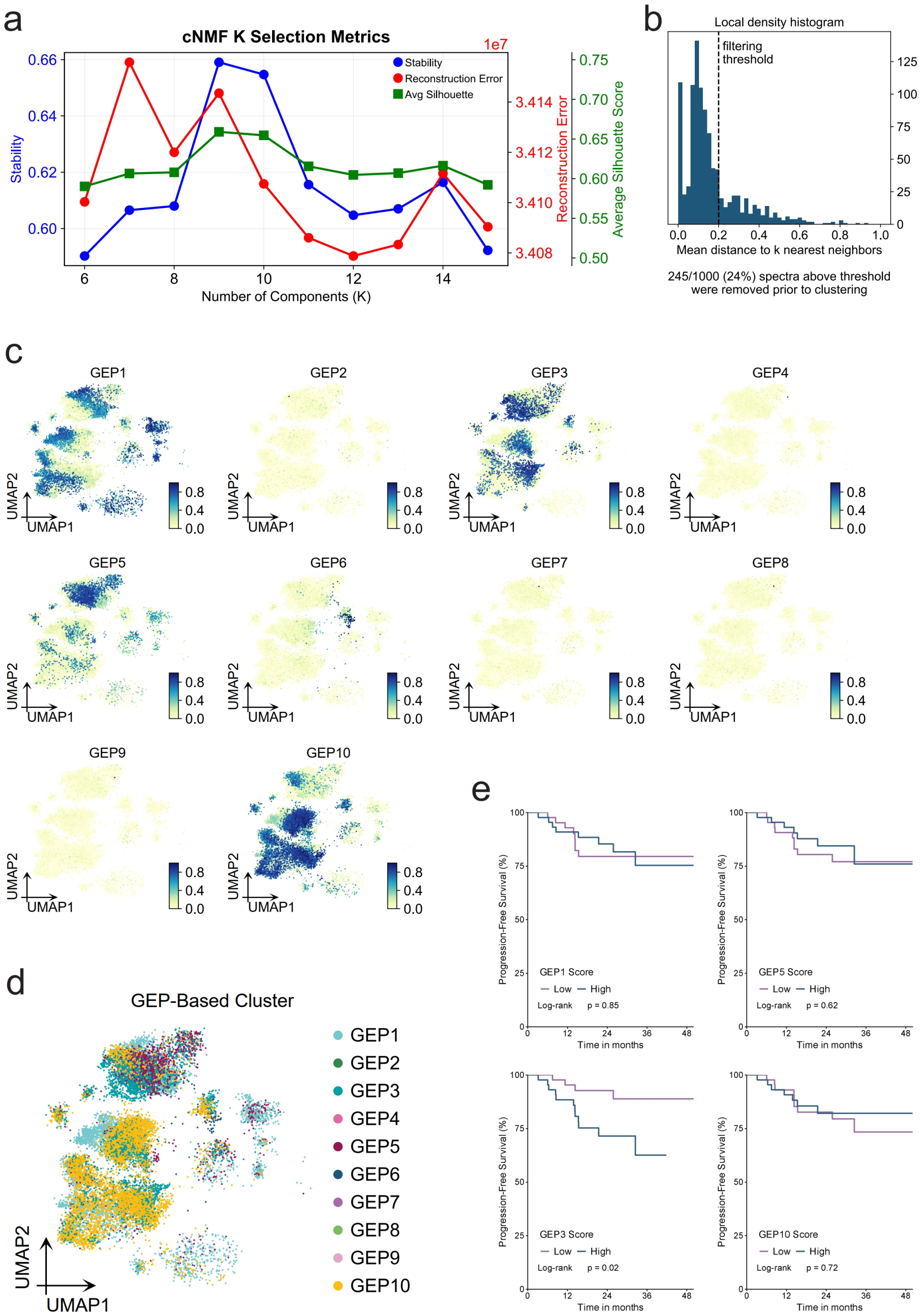

Figure S7. Identification and clinical evaluation of GEPs in malignant epithelial cells. (a) Component-selection metrics across different numbers of cNMF components. (b) Local-density distribution of the mean distance to the k nearest neighbors used for spectrum filtering. (c) UMAPs of malignant epithelial cells colored by the normalized usage score of each of the ten GEPs. (d) UMAP showing the dominant GEP assignment of each malignant epithelial cell. (e) Kaplan-Meier estimates of progression-free survival stratified by high versus low GEP1, GEP3, GEP5, and GEP10 scores. Abbreviations: cNMF, consensus non-negative matrix factorization; GEP, gene expression program; UMAP, uniform manifold approximation and projection.

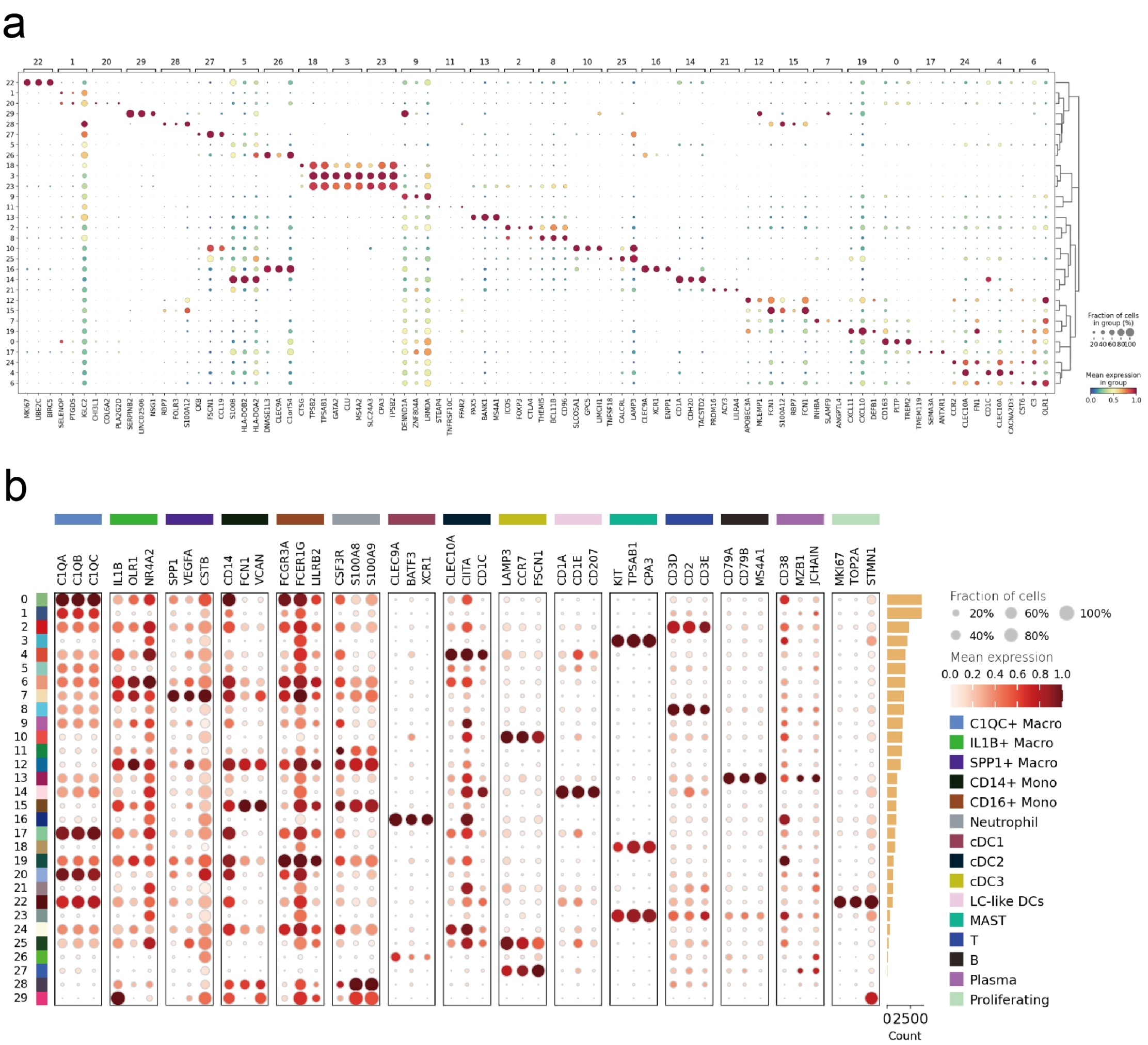

Figure S8. Myeloid sub-clustering and marker-based annotation reveal transcriptional heterogeneity in nasopharyngeal carcinoma. (a) Dot plot showing expression of top-ranked marker genes across myeloid clusters. (b) Dot plot displaying canonical myeloid and macrophage lineage markers used for subpopulation annotation.

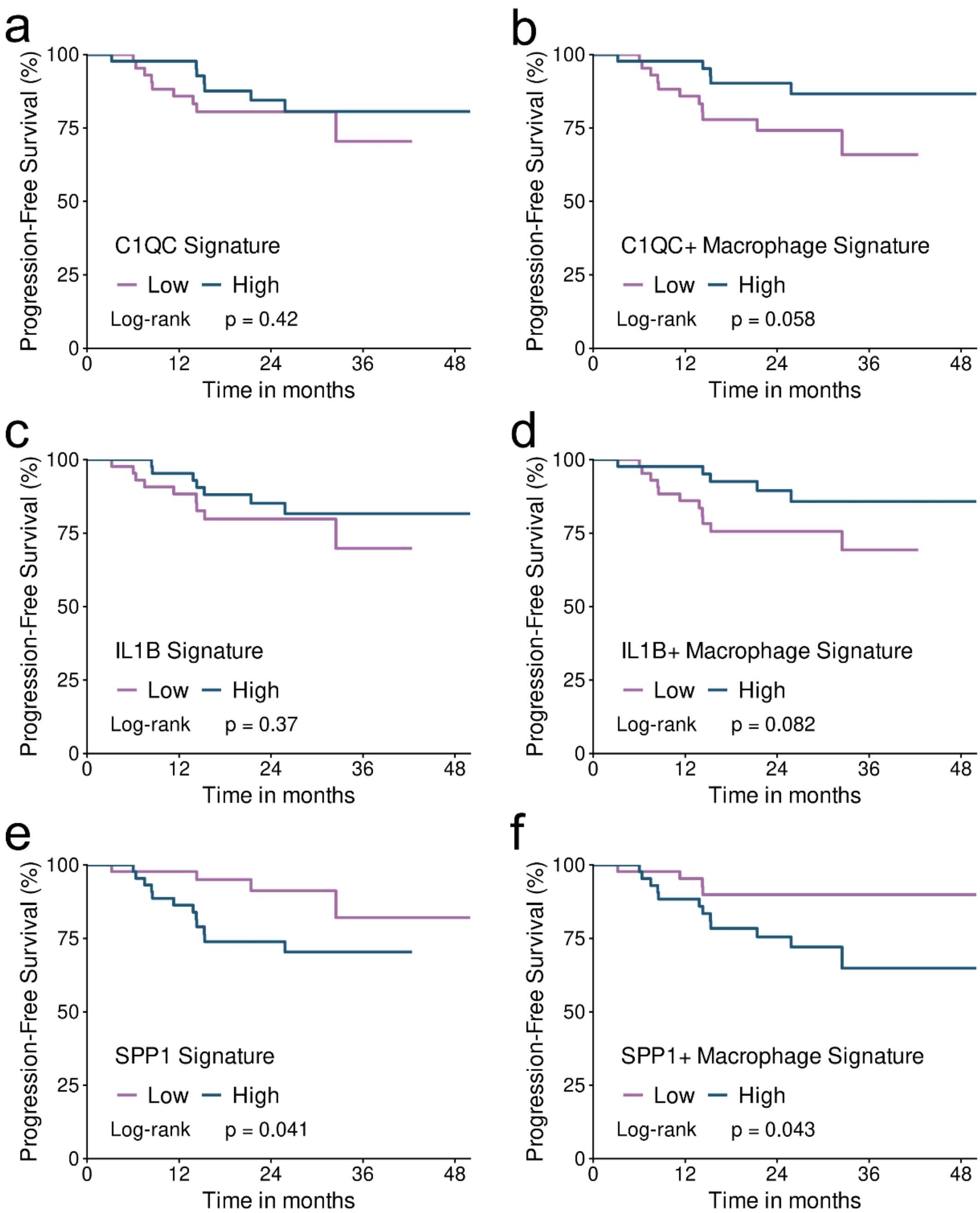

Figure S9. Prognostic relevance of macrophage-related signatures in NPC. Kaplan–Meier curves comparing progression-free survival between patients with high and low scores for the (a) C1QC signature, (b) C1QC^{+} macrophage signature, (c) IL1B signature, (d) IL1B^{+} macrophage signature, (e) SPP1 signature, and (f) SPP1^{+} macrophage signature in the bulk RNA-seq cohort. Patients were stratified into high- and low-score groups using the median score for each signature. Purple and blue lines indicate the low- and high-score groups, respectively. P values were calculated using two-sided log-rank tests. Abbreviations: NPC, nasopharyngeal carcinoma.

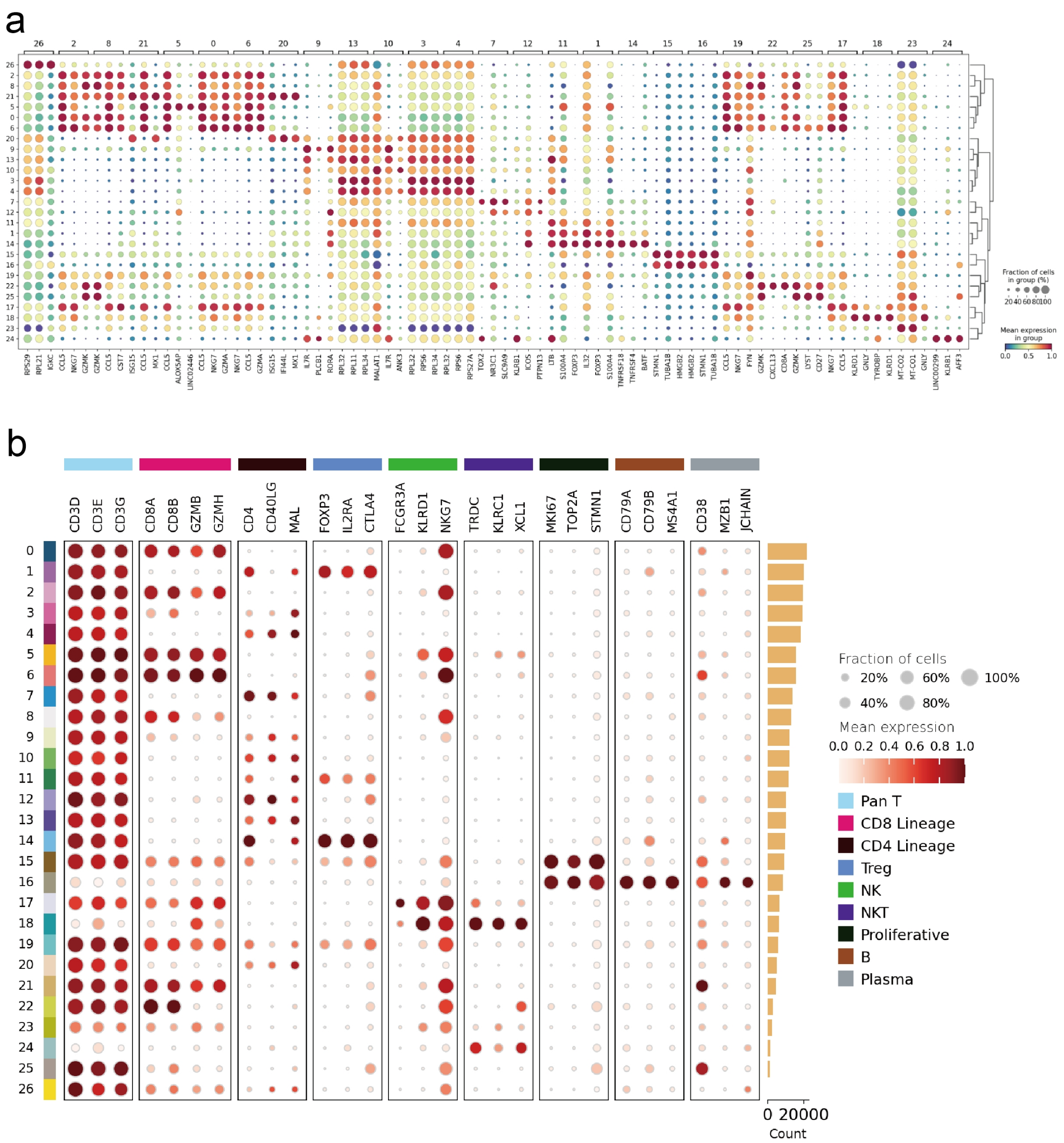

Figure S10. Marker-based annotation of T/NK-cell subclusters. (a) Dot plot of the top-ranked cluster-specific genes identified by one-versus-rest differential-expression analysis. (b) Dot plot of literature-curated canonical markers used to assign T/NK-cell subclusters to the indicated lineages. Rows denote clusters, columns denote genes, and the accompanying bars show the number of cells in each cluster. Dot size indicates the fraction of cells expressing each gene, and color indicates mean normalized expression among expressing cells, scaled from 0 to 1 for each gene. Abbreviations: NK, natural killer; NKT, natural killer T; T/NK, T and natural killer.

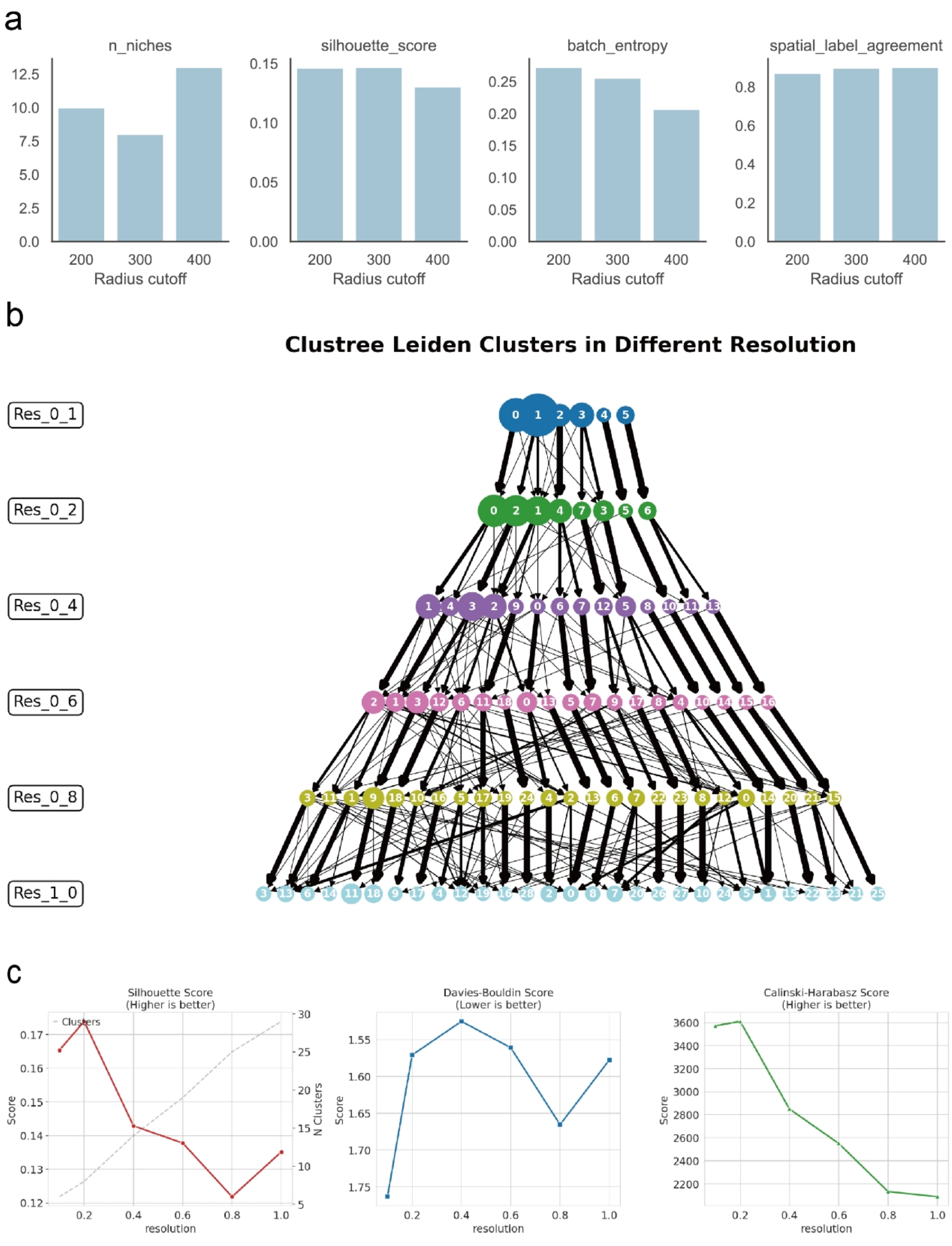

Figure S11. Sensitivity analysis and parameter selection for spatial niche definition. (a) Comparison of the number of inferred niches, silhouette score, batch entropy, and spatial-label agreement across spatial-radius cutoffs of 200, 300, and 400 μm. (b) Clustree visualization of relationships among Leiden clusters across resolutions from 0.1 to 1.0. Nodes represent clusters at each resolution, and connecting edges indicate shared spot membership between resolutions. (c) Silhouette, Davies–Bouldin, and Calinski–Harabasz scores across Leiden resolutions. Higher silhouette and Calinski–Harabasz scores and lower Davies–Bouldin scores indicate improved cluster separation.

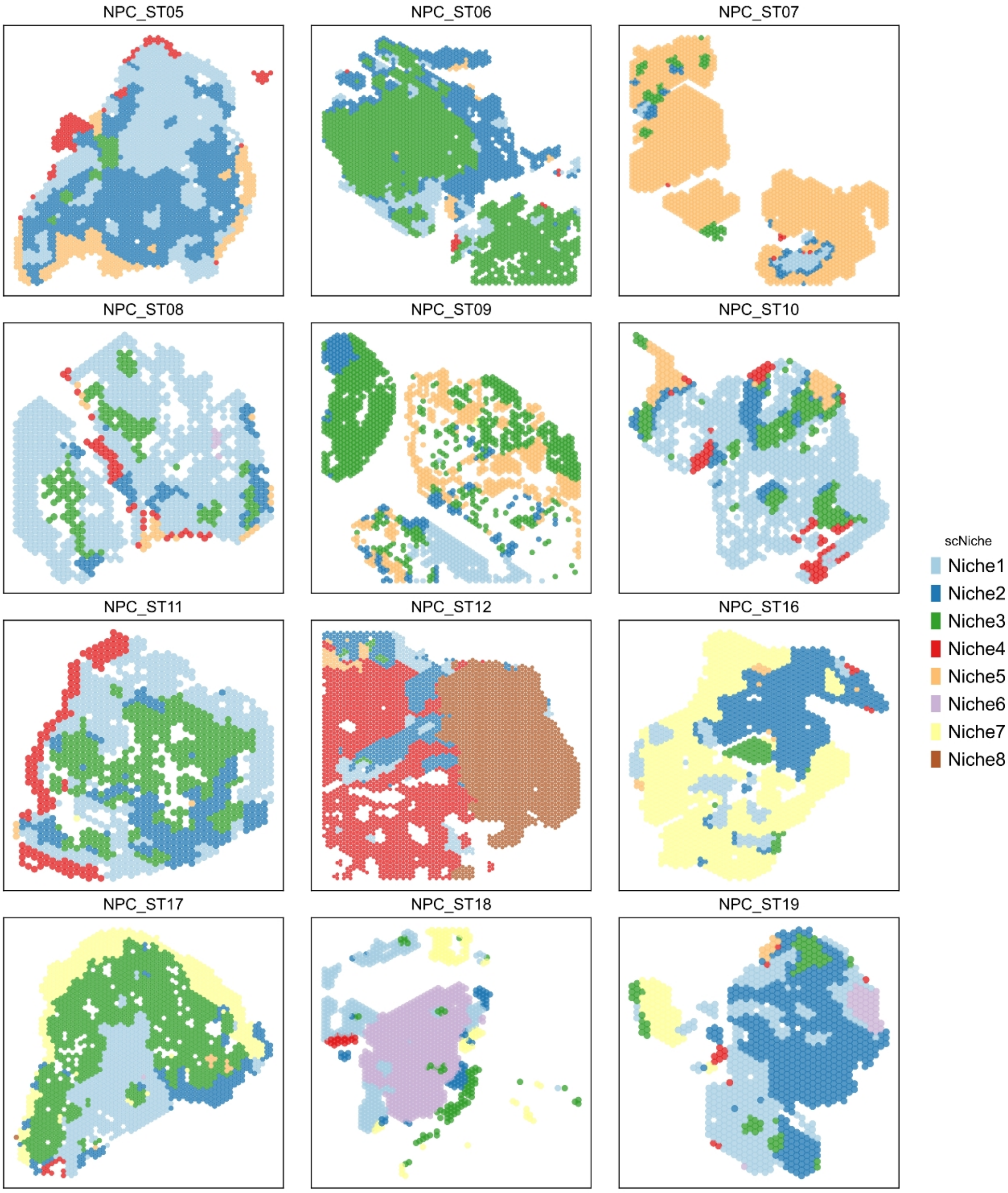

Figure S12. Spatial distribution of eight niches across EBV⁺ NPC specimens. Spatial maps showing the niche assignments of 21,087 spatial transcriptomic spots from 12 EBV⁺ NPC specimens following cross-sample integration and spatial clustering. Each spot is colored according to its assigned niche.

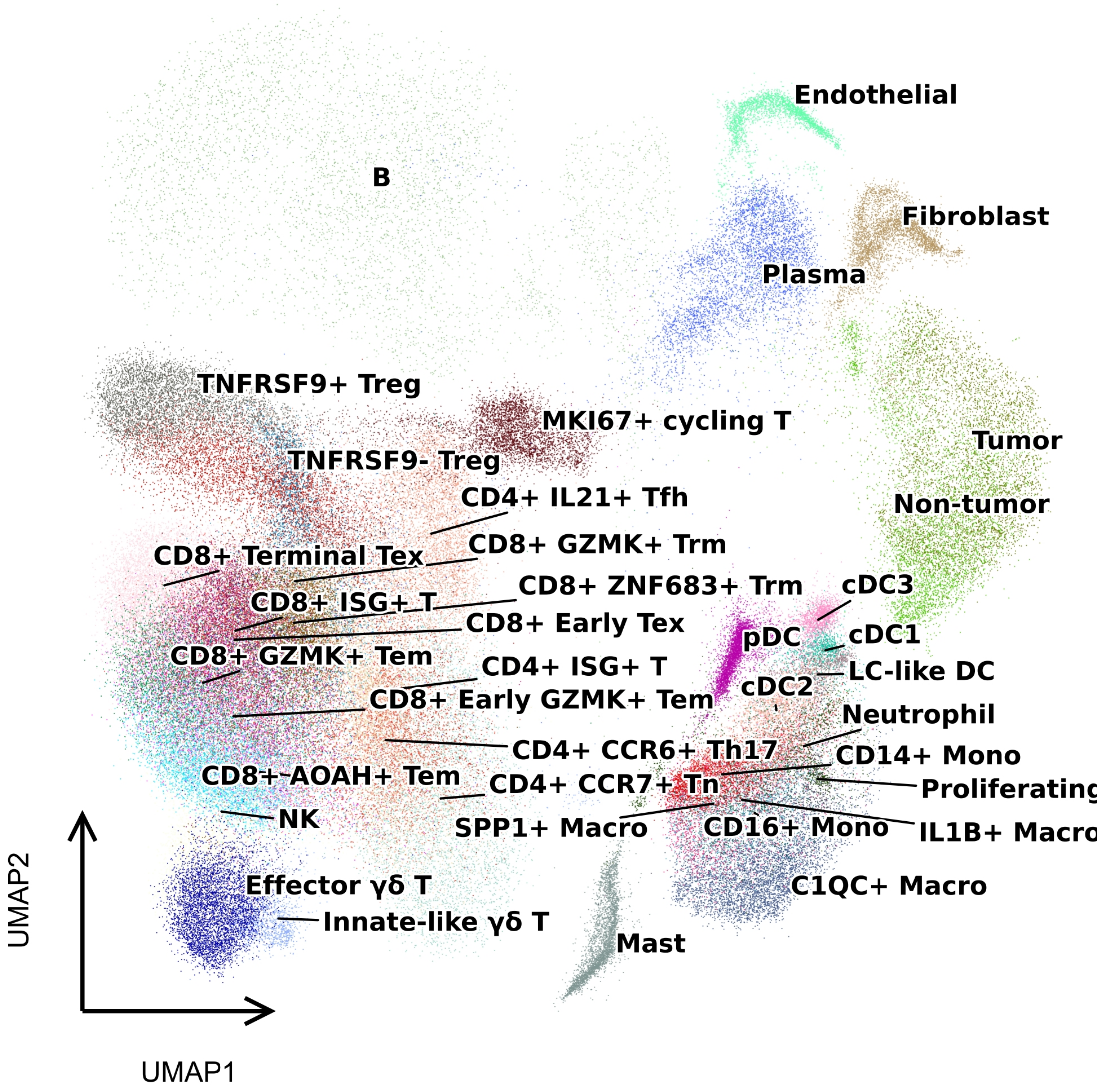

Figure S13. Subsampled single-cell transcriptomic reference atlas used for Tangram deconvolution. UMAP visualization of the single-cell transcriptomic reference atlas generated by subsampling up to 5,000 cells from each annotated cell subtype. For subtypes containing fewer than 5,000 cells, all available cells were retained. Each point represents one cell and is colored according to its annotated cell subtype. The subsampled cells and their annotations were used as the reference atlas for Tangram-based deconvolution of the spatial transcriptomic spots. Abbreviations: UMAP, uniform manifold approximation and projection.

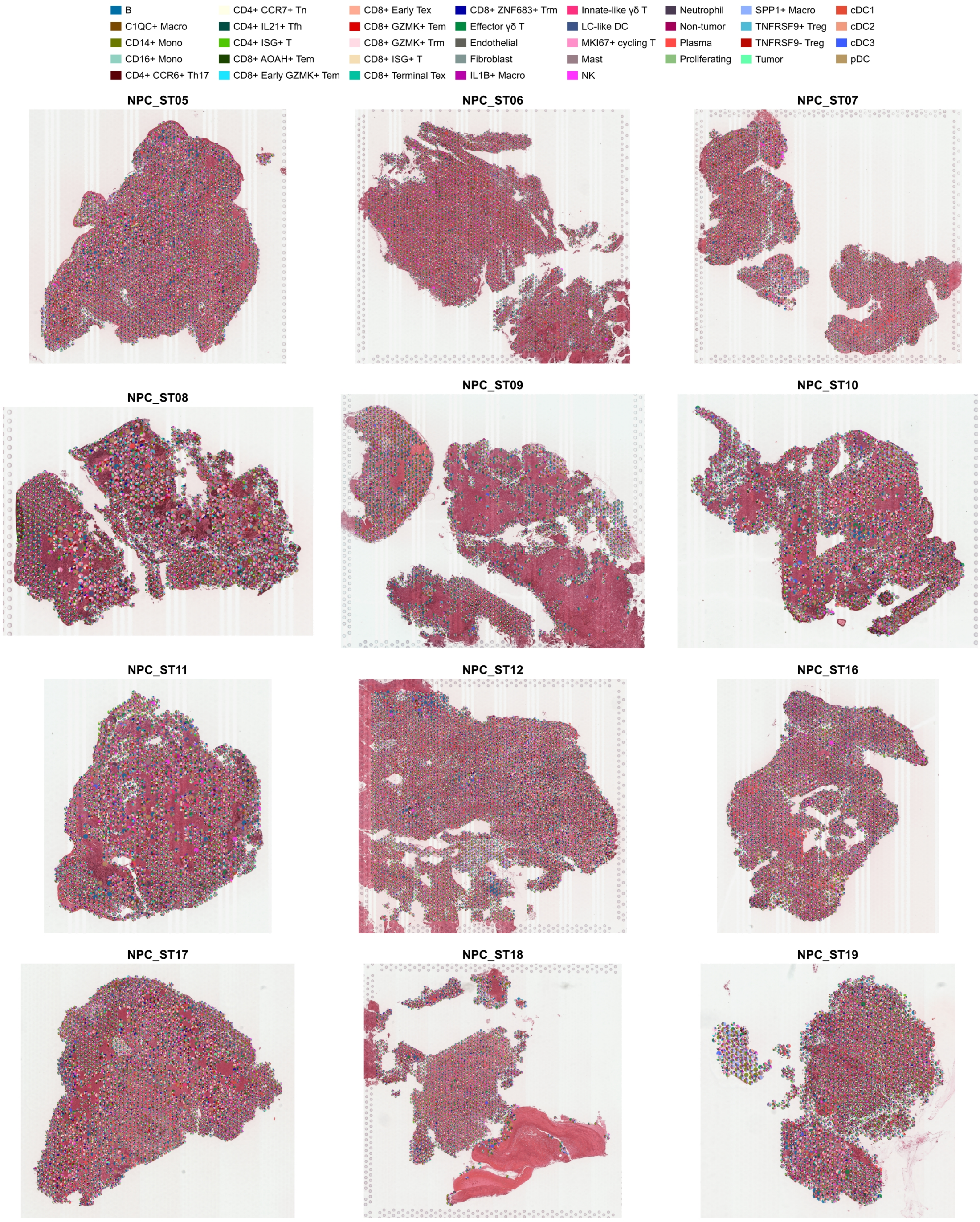

Figure S14. Tangram-inferred cell-type compositions of spatial transcriptomic spots across specimens. Spatial maps showing the cell-type proportions inferred by Tangram using the single-cell RNA-seq reference atlas. Each pie chart corresponds to one spatial transcriptomic spot, with the colored sectors indicating the estimated relative proportions of the annotated cell types.

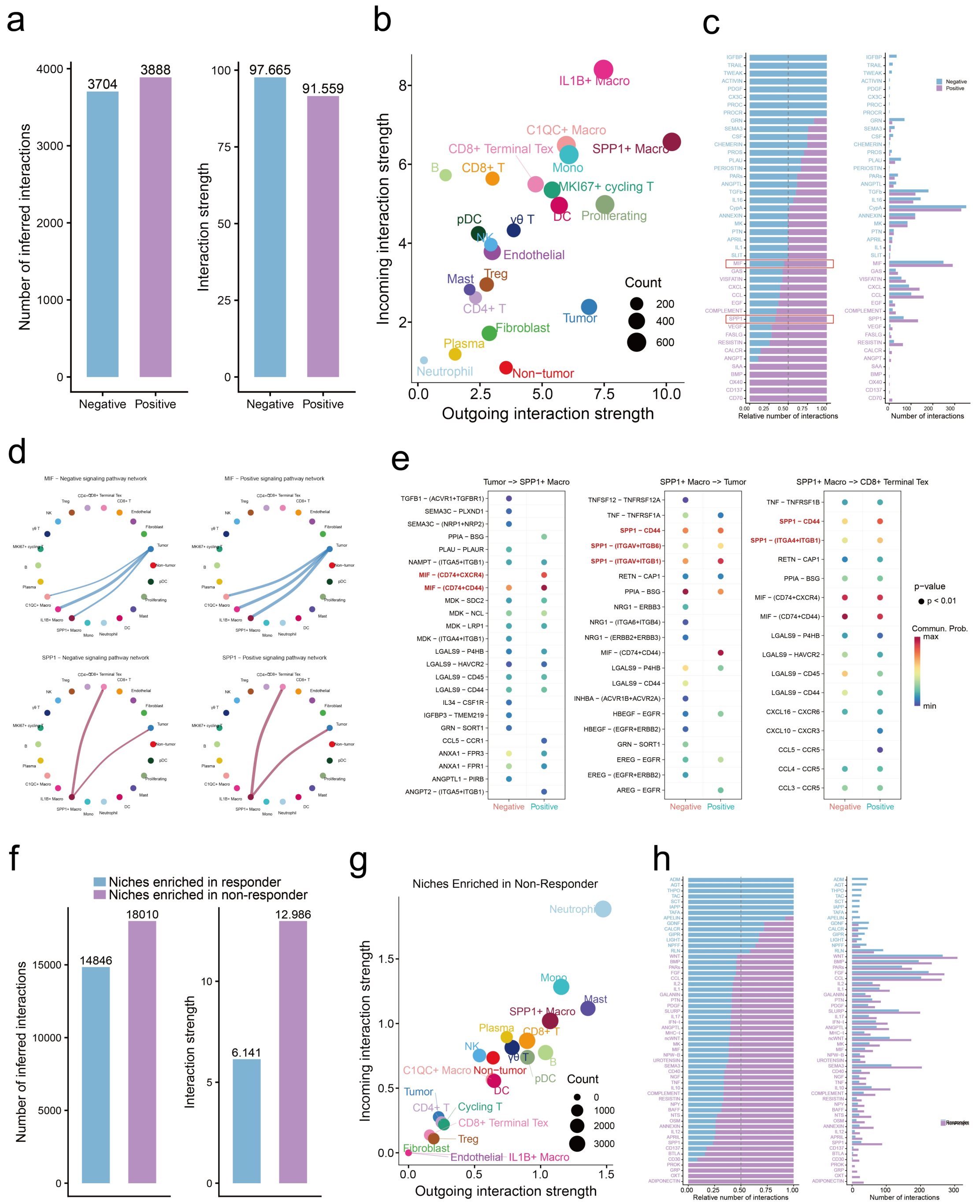

Figure S15. Cell–cell communication analysis identifies EBV-associated remodeling of MIF and SPP1 signaling in EBV^{+} NPC. (a) Total number and aggregate strength of cell interactions in EBV^{-} and EBV^{+} NPC. (b) Incoming and outgoing interaction strengths of the indicated cell populations in EBV^{+} NPC. (c) Relative and absolute numbers of interactions assigned to individual signaling pathways in EBV^{-} and EBV^{+} NPC. (d) Inferred MIF- and SPP1-signaling networks in EBV^{-} and EBV^{+} NPC. Edge width represents inferred communication strength. (e) Communication probabilities of selected ligand–receptor pairs for tumor-to-SPP1^{+} macrophage, SPP1^{+} macrophage-to-tumor, and SPP1^{+} macrophage-to-CD8^{+} terminally exhausted T-cell signaling. (f) Total number and aggregate strength of inferred interactions in spatial niches enriched in responders and non-responders. (g)

Incoming and outgoing interaction strengths of cell populations in non-responder-enriched niches. (h) Relative and absolute numbers of pathway-specific interactions in responder- and non-responder-enriched niches. Abbreviations: DC, dendritic cell; EBV, Epstein–Barr virus; Macro, macrophage; MIF, macrophage migration inhibitory factor; Mono, monocyte; NPC, nasopharyngeal carcinoma; SPP1, secreted phosphoprotein 1; Tex, exhausted T cell; Treg, regulatory T cell.

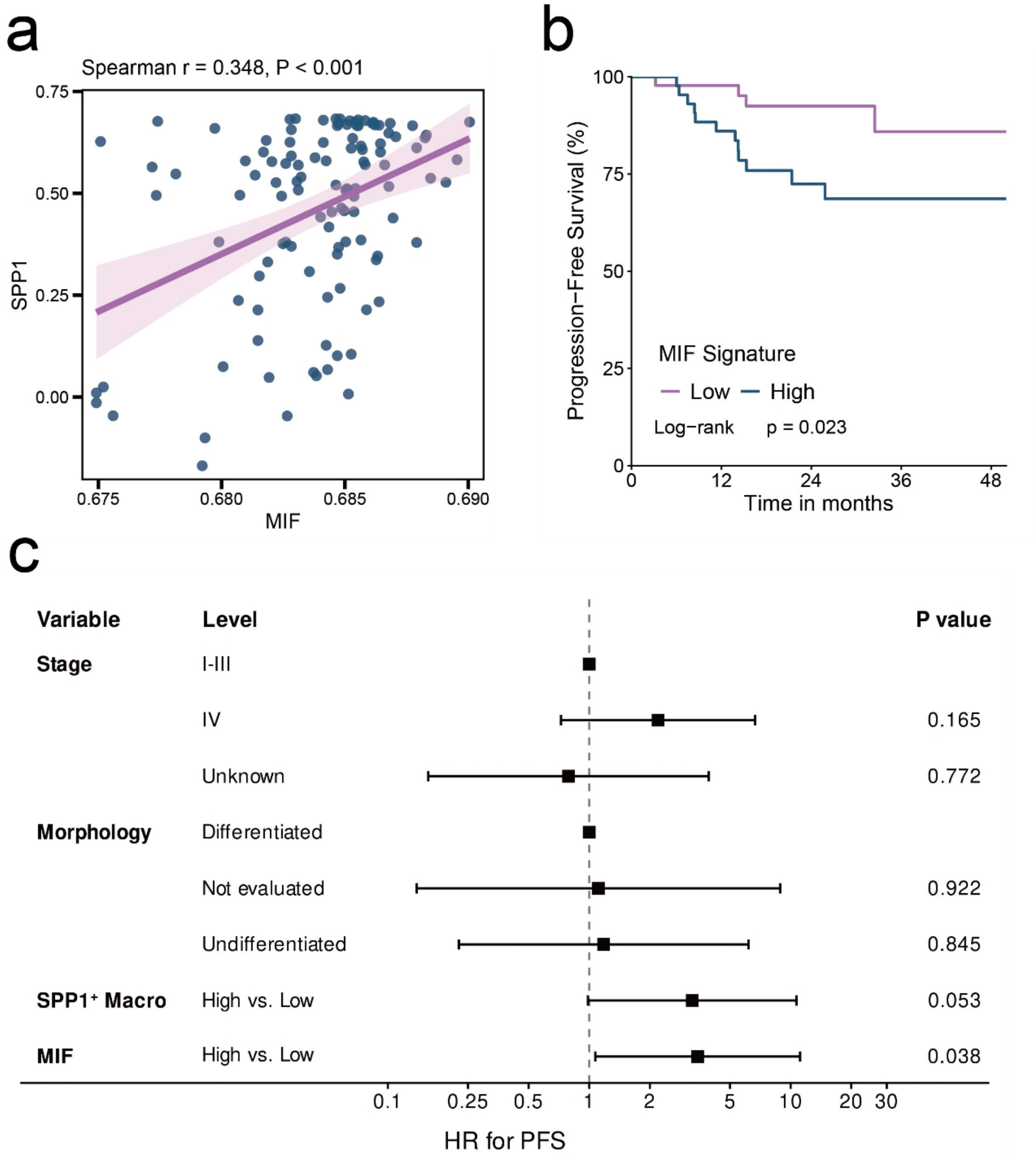

Figure S16. Association of MIF and the SPP1^{+} macrophage signature with clinical outcome. (a) Spearman correlation between MIF and SPP1 expression in the bulk RNA-seq cohort. Each point represents one patient sample; the shaded band indicates the 95% CI around the fitted line. (b) Kaplan–Meier analysis of PFS in patients stratified into high- and low-MIF-signature groups using median cutoff. The P value was calculated using the two-sided log-rank test. (c) Multivariable Cox proportional hazards analysis incorporating clinical stage, histological morphology, the SPP1^{+} macrophage signature, and the MIF signature. Squares indicate HRs, and horizontal lines indicate 95% CIs. Abbreviations: CI, confidence interval; HR, hazard ratio; MIF, macrophage migration inhibitory factor; PFS, progression-free survival; SPP1, secreted phosphoprotein 1.

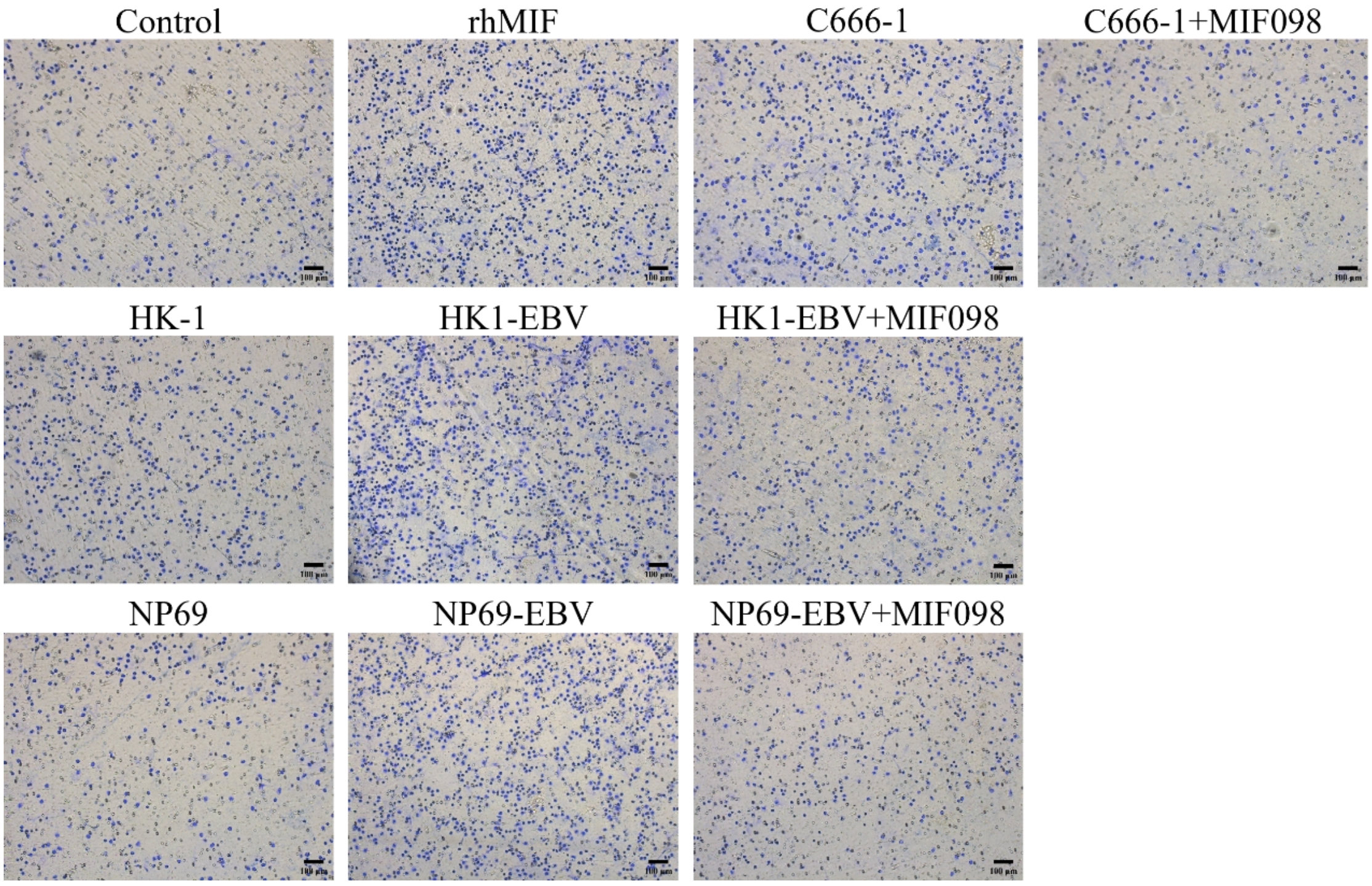

Figure S17. Representative Transwell images of primary monocyte migration following treatment with the indicated cell-derived CM, MIF098, or rhMIF. Scale bars, 100 µm.

Table S1. Summary of NPC samples for scRNA-seq.								
**New ID**	**Original ID**	**Dataset V2**	**Dataset V1**	**EBV status**	**Stage**	**Treatment**	**Platform**	**Library**
N01	N01	CNP0000428	GSE150430	Normal	Normal	Treatment-naive	10X Genomics V2	3′-Library
N02	N01_HRA003340	HRA003340	Not included	Normal	Normal	Treatment-naive	10X Genomics V3	3′-Library
N03	N02_HRA003340	HRA003340	Not included	Normal	Normal	Treatment-naive	10X Genomics V3	3′-Library
N04	N03_HRA003340	HRA003340	Not included	Normal	Normal	Treatment-naive	10X Genomics V3	3′-Library
N05	N52_10X	HRA000087	Not included	Normal	Normal	Treatment-naive	10X Genomics V2	3′-Library
N06	N53_10X	HRA000087	Not included	Normal	Normal	Treatment-naive	10X Genomics V2	3′-Library
N07	N54_10X	HRA000087	Not included	Normal	Normal	Treatment-naive	10X Genomics V2	3′-Library
N08	N55_10X	HRA000087	Not included	Normal	Normal	Treatment-naive	10X Genomics V2	3′-Library
N09	N57_10X	HRA000087	Not included	Normal	Normal	Treatment-naive	10X Genomics V2	3′-Library
N10	N61_10X	HRA000087	Not included	Normal	Normal	Treatment-naive	10X Genomics V2	3′-Library
N11	N68_10X	HRA000087	Not included	Normal	Normal	Treatment-naive	10X Genomics V2	3′-Library
N12	NLH_patient_11_rep1	PRJNA633797	GSE150825	Normal	Normal	Treatment-naive	10X Genomics V2	5′-Library
N13	NLH_patient_11_rep2	PRJNA633797	GSE150825	Normal	Normal	Treatment-naive	10X Genomics V2	5′-Library
N14	NLH_patient_12	PRJNA633797	GSE150825	Normal	Normal	Treatment-naive	10X Genomics V2	5′-Library
N15	NLH_patient_13	PRJNA633797	GSE150825	Normal	Normal	Treatment-naive	10X Genomics V2	5′-Library
P01	NPC_patient_1	PRJNA633797	GSE150825	Negative	III	Treatment-naive	10X Genomics V2	5′-Library
P02	NPC_patient_5	PRJNA633797	GSE150825	Negative	I-II	Treatment-naive	10X Genomics V2	5′-Library
P03	NPC_patient_7	PRJNA633797	GSE150825	Negative	III	Treatment-naive	10X Genomics V2	5′-Library

P04	NPC_patient_14	PRJNA633797	GSE150825	Negative	III	Treatment-naive	10X Genomics V2	5′-Library
P05	P03	CNP0000428	GSE150430	Negative	III	Treatment-naive	10X Genomics V2	3′-Library
P06	P10	CNP0000428	GSE150430	Negative	IV	Treatment-naive	10X Genomics V2	3′-Library
P07	YX156	ChiCTR2000032317	ChiCTR2000032317	Negative	IV	Treatment-naive	10X Genomics V3	3′-Library
P08	NPC33_10X	HRA000087	Not included	Positive	IV	Treatment-naive	10X Genomics V2	3′-Library
P09	NPC36_10X	HRA000087	Not included	Positive	IV	Treatment-naive	10X Genomics V2	3′-Library
P10	NPC41_10X	HRA000087	Not included	Positive	III	Treatment-naive	10X Genomics V2	3′-Library
P11	NPC46_10X	HRA000087	Not included	Positive	III	Treatment-naive	10X Genomics V2	3′-Library
P12	NPC47_10X	HRA000087	Not included	Positive	IV	Treatment-naive	10X Genomics V2	3′-Library
P13	NPC49_10X	HRA000087	Not included	Positive	III	Treatment-naive	10X Genomics V2	3′-Library
P14	NPC50_10X	HRA000087	Not included	Positive	I-II	Treatment-naive	10X Genomics V2	3′-Library
P15	NPC51_10X	HRA000087	Not included	Positive	IV	Treatment-naive	10X Genomics V2	3′-Library
P16	NPC58_10X	HRA000087	Not included	Positive	III	Treatment-naive	10X Genomics V2	3′-Library
P17	NPC59_10X	HRA000087	Not included	Positive	I-II	Treatment-naive	10X Genomics V2	3′-Library
P18	NPC60_10X	HRA000087	Not included	Positive	IV	Treatment-naive	10X Genomics V2	3′-Library
P19	NPC62_10X	HRA000087	Not included	Positive	I-II	Treatment-naive	10X Genomics V2	3′-Library
P20	NPC63_10X	HRA000087	Not included	Positive	III	Treatment-naive	10X Genomics V2	3′-Library
P21	NPC64_10X	HRA000087	Not included	Positive	I-II	Treatment-naive	10X Genomics V2	3′-Library
P22	NPC65_10X	HRA000087	Not included	Positive	IV	Treatment-naive	10X Genomics V2	3′-Library
P23	NPC66_10X	HRA000087	Not included	Positive	IV	Treatment-naive	10X Genomics V2	3′-Library

P24	NPC69_10X	HRA000087	Not included	Positive	III	Treatment-naive	10X Genomics V2	3′-Library
P25	NPC_SC_1802_Tumor_GEX	PRJNA680328	GSE162025	Positive	III	Treatment-naive	10X Genomics V1	5′-Library
P26	NPC_SC_1805_Tumor_GEX	PRJNA680328	GSE162025	Positive	IV	Treatment-naive	10X Genomics V1	5′-Library
P27	NPC_SC_1806_Tumor_GEX	PRJNA680328	GSE162025	Positive	IV	Treatment-naive	10X Genomics V1	5′-Library
P28	NPC_SC_1807_Tumor_GEX	PRJNA680328	GSE162025	Positive	III	Treatment-naive	10X Genomics V1	5′-Library
P29	NPC_SC_1808_Tumor_GEX	PRJNA680328	GSE162025	Positive	IV	Treatment-naive	10X Genomics V1	5′-Library
P30	NPC_SC_1810_Tumor_GEX	PRJNA680328	GSE162025	Positive	III	Treatment-naive	10X Genomics V1	5′-Library
P31	NPC_SC_1811_Tumor_GEX	PRJNA680328	GSE162025	Positive	III	Treatment-naive	10X Genomics V1	5′-Library
P32	NPC_SC_1813_Tumor_GEX	PRJNA680328	GSE162025	Positive	III	Treatment-naive	10X Genomics V1	5′-Library
P33	NPC_SC_1815_Tumor_GEX	PRJNA680328	GSE162025	Positive	I-II	Treatment-naive	10X Genomics V1	5′-Library
P34	NPC_SC_1816_Tumor_GEX	PRJNA680328	GSE162025	Positive	IV	Treatment-naive	10X Genomics V1	5′-Library
P35	NPC_patient_2	PRJNA633797	GSE150825	Positive	III	Treatment-naive	10X Genomics V2	5′-Library
P36	NPC_patient_3	PRJNA633797	GSE150825	Positive	III	Treatment-naive	10X Genomics V2	5′-Library
P37	NPC_patient_4	PRJNA633797	GSE150825	Positive	IV	Treatment-naive	10X Genomics V2	5′-Library
P38	NPC_patient_6	PRJNA633797	GSE150825	Positive	IV	Treatment-naive	10X Genomics V2	5′-Library
P39	NPC_patient_9	PRJNA633797	GSE150825	Positive	III	Treatment-naive	10X Genomics V2	5′-Library
P40	NPC_patient_10	PRJNA633797	GSE150825	Positive	III	Treatment-naive	10X Genomics V2	5′-Library
P41	P01	CNP0000428	GSE150430	Positive	III	Treatment-naive	10X Genomics V2	3′-Library
P42	P02	CNP0000428	GSE150430	Positive	IV	Treatment-naive	10X Genomics V2	3′-Library
P43	P04	CNP0000428	GSE150430	Positive	IV	Treatment-naive	10X Genomics V2	3′-Library

P44	P05	CNP0000428	GSE150430	Positive	III	Treatment-naive	10X Genomics V2	3′-Library
P45	P06	CNP0000428	GSE150430	Positive	III	Treatment-naive	10X Genomics V2	3′-Library
P46	P07	CNP0000428	GSE150430	Positive	III	Treatment-naive	10X Genomics V2	3′-Library
P47	P08	CNP0000428	GSE150430	Positive	I-II	Treatment-naive	10X Genomics V2	3′-Library
P48	P09	CNP0000428	GSE150430	Positive	IV	Treatment-naive	10X Genomics V2	3′-Library
P49	P11	CNP0000428	GSE150430	Positive	I-II	Treatment-naive	10X Genomics V2	3′-Library
P50	P12	CNP0000428	GSE150430	Positive	IV	Treatment-naive	10X Genomics V2	3′-Library
P51	P14	CNP0000428	GSE150430	Positive	IV	Treatment-naive	10X Genomics V2	3′-Library
P52	P15	CNP0000428	GSE150430	Positive	I-II	Treatment-naive	10X Genomics V2	3′-Library
P53	YX92	ChiCTR2000032317	ChiCTR2000032317	Positive	IV	Treatment-naive	10X Genomics V3	3′-Library
P54	YX93	ChiCTR2000032317	ChiCTR2000032317	Positive	IV	Treatment-naive	10X Genomics V3	3′-Library
P55	YX122	ChiCTR2000032317	ChiCTR2000032317	Positive	IV	Treatment-naive	10X Genomics V3	3′-Library
P56	YX124	ChiCTR2000032317	ChiCTR2000032317	Positive	IV	Treatment-naive	10X Genomics V3	3′-Library
P57	YX155	ChiCTR2000032317	ChiCTR2000032317	Positive	IV	Treatment-naive	10X Genomics V3	3′-Library

Abbreviations: EBV, Epstein–Barr virus; mIC, multiplex immunofluorescence; NPC, nasopharyngeal carcinoma; scRNA-seq, single-cell RNA sequencing.

Table S2. Summary of NPC samples for spatial transcriptomics.

Sample	Tissue	TNM stage*	Number of Spots per Tissue	Median Genes per Spot	Mean Reads per Spot	Response	Platform	Disease status at sampling	Therapeutic strategy	Chemotherapy regimens
ST5	NPC	IVa	2021	5100	114480	CR	Visium	treatment-naïve	toripalimab plus chemotherapy	Capecitabine
ST6	NPC	IVb	2614	1859	59441	CR	Visium	treatment-naïve	toripalimab plus chemotherapy	Paclitaxel + Cisplatin + 5-Fluorouracil
ST7	NPC	IVa	1939	5826	91934	PD	Visium	treatment-naïve	toripalimab plus chemotherapy	Gemcitabine + Anlotinib
ST8	NPC	IVb	1209	2854	151387	CR	Visium	treatment-naïve	toripalimab plus chemotherapy	Paclitaxel + Cisplatin + 5-Fluorouracil
ST9	NPC	III	2602	1118	74173	PD	Visium	treatment-naïve	toripalimab plus chemotherapy	Tegafur, Gimeracil and Oteracil Potassium
ST10	NPC	IVa	1309	2625	91293	PR	Visium	treatment-naïve	toripalimab plus chemotherapy	Gemcitabine + Cisplatin
ST11	NPC	IVb	1272	2500	90144	CR	Visium	treatment-naïve	toripalimab plus chemotherapy	Gemcitabine + Cisplatin
ST12	NPC	IVa	3669	6947	76081	CR	Visium	treatment-naïve	toripalimab plus chemotherapy	Paclitaxel + Tegafur, Gimeracil and Oteracil Potassium
ST16	NPC	IVb	1556	8502	104781	PD	Visium	treatment-naïve	toripalimab plus chemotherapy	Paclitaxel + Carboplatin
ST17	NPC	IVb	2067	2171	82985	SD	Visium	treatment-naïve	toripalimab plus chemotherapy	Tegafur, Gimeracil and Oteracil Potassium
ST18	NPC	III	1977	1696	87660	PR	Visium	treatment-naïve	toripalimab plus chemotherapy	Docetaxel
ST19	NPC	IVb	1086	7288	129897	PD	Visium	treatment-naïve	toripalimab plus chemotherapy	Gemcitabine

*: TNM stage of NPC were determined according to the 8th edition of the International Union against Cancer (UICC) and American Joint Committee on Cancer (AJCC) staging system.
Abbreviations: CR, Complete response; PD, Progressive disease; PR, Partial response; SD, Stable disease.

Table S3. Summary of NPC samples for mIC.			
**Patient ID**	**EBV status**	**Stage**	**Treatment**
P01_mIC	Positive	IVA	Treatment-naïve
P02_mIC	Negative	IVA	Treatment-naïve

Abbreviations: EBV, Epstein–Barr virus; mIC, multiplex immunofluorescence; NPC, nasopharyngeal carcinoma.

Table S4. Summary of NPC samples for bulk RNA-seq.				
**SampleID**	**Time in months**	**PFS status**	**Stage**	**Morphology**
NPC0001PT00001T00001	N/A	N/A	III	N/A
NPC0001PT00004T00004	21.1	0	IV	N/A
NPC0001PT00005T00005	55.23	0	III	N/A
NPC0001PT00012T00012	36	0	Unknown	differentiated
NPC0001PT00014T00014	13.93	0	IV	mixed (round & spindle)
NPC0001PT00020T00020	39.5	0	III	undifferentiated, spindle
NPC0001PT00027T00027	16.77	0	IV	undifferentiated, round
NPC0001PT00029T00029	39.3	0	Unknown	differentiated
NPC0001PT00044T00044	42.4	0	III	undifferentiated, spindle
NPC0001PT00046T00046	37.1	0	III	mixed (round & spindle)
NPC0001PT00048T00048	6.34	1	III	undifferentiated, round
NPC0001PT00054T00054	17.23	0	III	undifferentiated, spindle
NPC0001PT00055T00055	32.46	1	IV	undifferentiated, spindle
NPC0001PT00056T00056	13.83	1	IV	undifferentiated, round
NPC0001PT00061T00061	40.07	0	IV	differentiated
NPC0001PT00065T00065	30.97	0	IV	undifferentiated, round
NPC0001PT00069T00069	25.82	1	IV	differentiated
NPC0001PT00070T00070	33.33	0	Unknown	differentiated
NPC0001PT00071T00071	5	0	III	mixed (round & spindle)
NPC0001PT00075T00075	29.07	0	III	mixed (round & spindle)
NPC0001PT00078T00078	21.39	1	III	mixed (round & spindle)
NPC0001PT00079T00079	8.51	1	III	undifferentiated, spindle
NPC0001PT00096T00096	N/A	N/A	Unknown	mixed (round & spindle)
NPC0001PT00097T00097	N/A	N/A	Unknown	undifferentiated, round
NPC0001PT00099T00099	39.37	0	III	undifferentiated, round
NPC0001PT00100T00100	30.97	0	Unknown	differentiated
NPC0001PT00144T00144	N/A	N/A	Unknown	undifferentiated, spindle
NPC0001PT00151T00151	15.28	1	Unknown	undifferentiated, round
NPC0001PT00152T00152	37.9	0	Unknown	mixed (round & spindle)
NPC0001PT00173T00173	41.8	0	III	mixed (round & spindle)
NPC0001PT00177T00177	8.41	1	III	mixed (round & spindle)
NPC0001PT00178T00178	N/A	N/A	IV	undifferentiated, round
NPC0001PT00192T00192	N/A	N/A	Unknown	undifferentiated, round
NPC0001PT00196T00196	34.5	0	III	mixed (round & spindle)
NPC0001PT00204T00204	11.3	1	IV	undifferentiated, round
NPC0001PT00207T00207	37.73	0	III	undifferentiated, round
NPC0001PT00209T00209	51.33	0	III	mixed (round & spindle)
NPC0001PT00221T00221	7.49	1	III	mixed (round & spindle)
NPC0001PT00226T00226	29.27	0	III	mixed (round & spindle)
NPC0001PT00229T00229	N/A	N/A	III	undifferentiated, round

NPC0001PT00233T00233	16	0	Unknown	undifferentiated, spindle
NPC0001PT00234T00234	23.13	0	Unknown	mixed (round & spindle)
NPC0001PT00235T00235	23.07	0	Unknown	undifferentiated, round
NPC0001PT00236T00236	N/A	N/A	Unknown	mixed (round & spindle)
NPC0001PT00237T00237	36.9	0	Unknown	undifferentiated, round
NPC0001PT00240T00240	36.6	0	Unknown	mixed (round & spindle)
NPC0001PT00243T00243	N/A	N/A	Unknown	mixed (round & spindle)
NPC0001PT00258T00258	35.67	0	III	mixed (round & spindle)
NPC0001PT00260T00260	14.29	1	IV	mixed (round & spindle)
NPC0001PT00263T00263	36.43	0	III	mixed (round & spindle)
NPC0001PT00264T00264	30.2	0	Unknown	undifferentiated, round
NPC0001PT00278T00278	37.13	0	IV	N/A
NPC0001PT00286T00286	20.13	0	III	mixed (round & spindle)
NPC0001PT00289T00289	N/A	N/A	Unknown	mixed (round & spindle)
NPC0001PT00290T00290	N/A	N/A	Unknown	mixed (round & spindle)
NPC0001PT00293T00293	N/A	N/A	Unknown	undifferentiated, spindle
NPC0001PT00294T00294	32.17	0	III	mixed (round & spindle)
NPC0001PT00296T00296	20.73	0	III	undifferentiated, spindle
NPC0001PT00298T00298	29.77	0	III	undifferentiated, round
NPC0001PT00300T00300	26.8	0	III	undifferentiated, round
NPC0001PT00302T00302	N/A	N/A	Unknown	undifferentiated, round
NPC0001PT00305T00305	N/A	N/A	Unknown	undifferentiated, round
NPC0001PT00306T00306	N/A	N/A	Unknown	mixed (round & spindle)
NPC0001PT00312T00312	6.01	1	III	undifferentiated, round
NPC0001PT00320T00320	N/A	N/A	Unknown	undifferentiated, spindle
NPC0001PT00322T00322	N/A	N/A	Unknown	mixed (round & spindle)
NPC0001PT00329T00329	N/A	N/A	Unknown	mixed (round & spindle)
NPC0001PT00331T00331	N/A	N/A	Unknown	undifferentiated, round
NPC0001PT00332T00332	26.6	0	Unknown	mixed (round & spindle)
NPC0001PT00336T00336	19.33	0	III	undifferentiated, round
NPC0001PT00337T00337	N/A	N/A	Unknown	N/A
NPC0001PT00338T00338	N/A	N/A	IV	undifferentiated, round
NPC0001PT00341T00341	14.23	1	IV	differentiated
NPC0001PT00344T00344	25.07	0	Unknown	undifferentiated, round
NPC0001PT00346T00346	17.57	0	IV	differentiated
NPC0001PT00349T00349	29.43	0	II	differentiated
NPC0001PT00354T00354	17.5	0	Unknown	undifferentiated, round
NPC0001PT00355T00355	32.83	0	III	mixed (round & spindle)
NPC0001PT00357T00357	24.93	0	III	undifferentiated, round
NPC0001PT00369T00369	28	0	III	mixed (round & spindle)
NPC0001PT00370T00370	26.9	0	IV	mixed (round & spindle)
NPC0001PT00383T00383	7.1	0	Unknown	undifferentiated, round
NPC0001PT00388T00388	12.1	0	III	undifferentiated, round

NPC0001PT00389T00389	19.77	0	I	mixed (round & spindle)
NPC0001PT00391T00391	26.77	0	Unknown	undifferentiated, round
NPC0001PT00392T00392	19.63	0	III	mixed (round & spindle)
NPC0001PT00396T00396	15.34	1	Unknown	undifferentiated, round
NPC0001PT00406T00406	30.9	0	I	undifferentiated, round
NPC0001PT00407T00407	26.07	0	I	undifferentiated, round
NPC0001PT00414T00414	12.43	0	Unknown	mixed (round & spindle)
NPC0001PT00421T00421	N/A	N/A	IV	undifferentiated, spindle
NPC0001PT00422T00422	28.77	0	III	mixed (round & spindle)
NPC0001PT00426T00426	26.8	0	IV	mixed (round & spindle)
NPC0001PT00431T00431	N/A	N/A	Unknown	undifferentiated, spindle
NPC0001PT00437T00437	22.13	0	IV	undifferentiated, spindle
NPC0001PT00438T00438	14.63	0	III	N/A
NPC0001PT00439T00439	13.4	0	Unknown	undifferentiated, round
NPC0001PT00443T00443	21.53	0	III	undifferentiated, round
NPC0001PT00445T00445	12.87	0	IV	mixed (round & spindle)
NPC0001PT00450T00450	9.27	0	III	undifferentiated, round
NPC0001PT00452T00452	12.9	0	IV	mixed (round & spindle)
NPC0002PT00013T00013	14.29	1	IV	N/A
NPC0002PT00015T00015	N/A	N/A	Unknown	Not evaluated
NPC0002PT00016T00016	N/A	N/A	Unknown	mixed (round & spindle)
NPC0002PT00018T00018	28.57	0	I	N/A
NPC0002PT00019T00019	33.67	0	IV	undifferentiated, round
NPC0002PT00034T00034	21.27	0	III	mixed (round & spindle)
NPC0002PT00036T00036	22.77	0	IV	mixed (round & spindle)
NPC0002PT00037T00037	18.4	0	III	differentiated
NPC0002PT00038T00038	27.27	0	II	N/A
NPC0002PT00039T00039	12.3	0	I	N/A
NPC0002PT00040T00040	22.9	0	IV	N/A
NPC0002PT00041T00041	3.19	1	III	N/A

Table S5. Gene sets used in the study.	
**Gene set**	**Genes**
**Curated Stemness Geneset**	
**Stemness**	DNMT3B, PFAS, XRCC5, HAUS6, TET1, IGF2BP1, PLAA, TEX10, MSH6, DLGAP5, SKIV2L2, SOHLH2, RRAS2, PAICS, CPSF3, LIN28B, IPO5, BMPR1A, ZNF788, ASCC3, FANCB, HMGA2, TRIM24, ORC1, HDAC2, HESX1, INHBE, MIS18A, DCUN1D5, MRPL3, CENPH, MYCN, HAUS1, GDF3, TBCE, RIOK2, BCKDHB, RAD1, NREP, ADH5, PLRG1, ROR1, RAB3B, DIAPH3, GNL2, FGF2, NMNAT2, KIF20A, CENPI, DDX1, XXYLT1, GPR176, BBS9, C14orf166, BOD1, CDC123, SNRPD3, FAM118B, DPH3, EIF2B3, RPF2, APLP1, DACT1, PDHB, C14orf119, DTD1, SAMM50, CCL26, MED20, UTP6, RARS2, ARMCX2, RARS, MTHFD2, DHX15, HTR7, MTHFD1L, ARMC9, XPOT, IARS, HDX, ACTRT3, ERCC2, TBC1D16, GARS, KIF7, UBE2K, SLC25A3, ICMT, UGGT2, ATP11C, SLC24A1, EIF2AK4, GPX8, ALX1, OSTC, TRPC4, HAS2, FZD2, TRNT1, MMADHC, SNX8, CDH6, HAT1, SEC11A, DIMT1, TM2D2, FST, GBE1
**Myeloid Immunity Gene Sets**	
**Anti-inflammatory**	TIGIT, IDO1, LGALS3, PDCD1, FOXP3, ENTPD1, CD274, CSF2, CTLA4, CXCL12, CXCL5, IL8, MIF, PTGS2, VEGFA
**Pro inflammatory**	IL1A, IL1B, TNF, IFNG, TBX21, CCL3, CCL4, PRF1, GZMA, GZMB, GZMK, GZMH, CD8A, FASLG, CCL2, CCL20, IL2, IL6, IL12A, IL17A, IL23A, PTGS2, TLR4, TNF
**M1 Macrophage Polarization**	IL12A, IL23A, IL12B, TNF, IL6, CD86, HLA-DRA, IL1B, MARCO, NOS2, IL12RB1, FCGR1A, CD80, IL23A, CXCL9, CXCL10, CXCL11, CD86, IL1A, IL1B, IL6, TNF, HLA-DRB1, CCL5, IRF5, IRF1, CD40, IDO1, KYNU, CCR7, PTPRC, CD68, CSF1R, HLA-DRA, LY75, CD14
**M2 Macrophage Polarization**	CD163, MRC1, MSR1, MARCO, IL10, IL1RN, IL1R2, TGFB1, TGFB2, TGFB3, VEGFA, VEGFB, VEGFC, ADM, SLC2A3, LDHA, HK2, MMP9, MMP14, CTSB, CTSD, LGMN, TIMP1, LGALS3, MERTK, AXL, TREM2, SPP1, VCAN, CD44, GPNMB, FOLR2, LYVE1, STAB1, VSIG4, LILRB4, CD274, PDCD1LG2, CD276, VTCN1
**Type I Interferon response**	IRF1, IFIH1, IFITM3, DDX58, IFI44L, IFI6, IFITM2, NAMPT, OASL, RTP4, TREX1, C15orf48, ADAR, FAM46C, SLC11A1, LY6E, MCOLN2, APOBEC3G, IL15, ISG15, MX1, TLR3
**Type II Interferon Response**	IFNG, CXCL9, CXCL10, STAT1, HLA-DRA, HLA-DRB1, CD274, CDKN1A, MYC, SMAD7, IRF1
**Hypoxia**	ALDH2, ALDOA, ADM, BCL2, BCL2L1, FOS, JUN, SRC, CREBBP, CCR6, CEBPB, ENO1, FAS, FASLG, FKBP4, GAPDH, SLC2A1, SLC2A3, CSF2, IFNG, IFNB1, IL13, IL1R1, IL2RA, IL4, IL5, IL6R, IL8, IL10RA, IL15RA, ITGA6, ITK, JUND, HLA-DRA, MAP3K5, MAP2K1, MAP2K2, MIF, NFATC1, NFKB2, NFKBIE, TP53, TGM6, TNF, TNFRSF1B, TRADD, TNFSF10, TRAP1, VEGFA
**Angiogenesis**	ANGPT1, CAV1, CDH5, CNKSR1, CSF2, CSF2RA, CTNNB1, CYBB, DUSP1, DUSP2, DUSP6, EGF, ERG, FGF10, FGF18, FGF2, FGF7, FGFR1, FLT1, FN1, FYN, HBEGF, HRAS, IL2, IL3, IL3RA, IL5, IL5RA, JAK3, KIT, KITLG, LAT, MAP2K1, MAP2K2, MAPK11, MAPK12, MAPK13, MAPK14, MARCKSL1, NCF2, NFIL3, PDGFA, PDGFB, PDGFRA, PRKCA, PROK2, PSMB8, PSMB9, PSME2, RAF1, RASAL1, RASGRF2, RICTOR, SPHK1, SPTBN1, TEK, VAV1, VAV2, CXCL8, SPP1, MIF, VEGFA, VEGFC, VWA5A
**MSigDB Hallmark Gene Sets**	
**Tnfa Signaling Via Nfkb**	JUNB, CXCL2, ATF3, NFKBIA, TNFAIP3, PTGS2, CXCL1, IER3, CD83, CCL20, CXCL3, MAFF, NFKB2, TNFAIP2, HBEGF, KLF6,

	BIRC3, PLAUR, ZFP36, ICAM1, JUN, EGR3, IL1B, BCL2A1, PPP1R15A, ZC3H12A, SOD2, NR4A2, IL1A, RELB, TRAF1, BTG2, DUSP1, MAP3K8, ETS2, F3, SDC4, EGR1, IL6, TNF, KDM6B, NFKB1, LIF, PTX3, FOSL1, NR4A1, JAG1, CCL4, GCH1, CCL2, RCAN1, DUSP2, EHD1, IER2, REL, CFLAR, RIPK2, NFKBIE, NR4A3, PHLDA1, IER5, TNFSF9, GEM, GADD45A, CXCL10, PLK2, BHLHE40, EGR2, SOCS3, SLC2A6, PTGER4, DUSP5, SERPINB2, NFIL3, SERPINE1, TRIB1, TIPARP, RELA, BIRC2, CXCL6, LITAF, TNFAIP6, CD44, INHBA, PLAU, MYC, TNFRSF9, SGK1, TNIP1, NAMPT, FOSL2, PNRC1, ID2, CD69, IL7R, EFNA1, PHLDA2, PFKFB3, CCL5, YRDC, IFNGR2, SQSTM1, BTG3, GADD45B, KYNU, G0S2, BTG1, MCL1, VEGFA, MAP2K3, CDKN1A, CCN1, TANK, IFIT2, IL18, TUBB2A, IRF1, FOS, OLR1, RHOB, AREG, NINJ1, ZBTB10, PLPP3, KLF4, CXCL11, SAT1, CSF1, GPR183, PMEPA1, PTPRE, TLR2, ACKR3, KLF10, MARCKS, LAMB3, CEBPB, TRIP10, F2RL1, KLF9, LDLR, TGIF1, RNF19B, DRAM1, B4GALT1, DNAJB4, CSF2, PDE4B, SNN, PLEK, STAT5A, DENND5A, CCND1, DDX58, SPHK1, CD80, TNFAIP8, CCNL1, FUT4, CCRL2, SPSB1, TSC22D1, B4GALT5, SIK1, CLCF1, NFE2L2, FOSB, PER1, NFAT5, ATP2B1, IL12B, IL6ST, SLC16A6, ABCA1, HES1, BCL6, IRS2, SLC2A3, CEBPD, IL23A, SMAD3, TAP1, MSC, IFIH1, IL15RA, TNIP2, BCL3, PANX1, FJX1, EDN1, EIF1, BMP2, DUSP4, PDLIM5, ICOSLG, GFPT2, KLF2, TNC, SERPINB8, MXD1
**Hypoxia**	PGK1, PDK1, GBE1, PFKL, ALDOA, ENO2, PGM1, NDRG1, HK2, ALDOC, GPI, MXI1, SLC2A1, P4HA1, ADM, P4HA2, ENO1, PFKP, AK4, FAM162A, PFKFB3, VEGFA, BNIP3L, TPI1, ERO1A, KDM3A, CCNG2, LDHA, GYS1, GAPDH, BHLHE40, ANGPTL4, JUN, SERPINE1, LOX, GCK, PPFIA4, MAFF, DDIT4, SLC2A3, IGFBP3, NFIL3, FOS, RBPJ, HK1, CITED2, ISG20, GALK1, WSB1, PYGM, STC1, ZNF292, BTG1, PLIN2, CSRP2, VLDLR, JMJD6, EXT1, F3, PDK3, ANKZF1, UGP2, ALDOB, STC2, ERRFI1, ENO3, PNRC1, HMOX1, PGF, GAPDHS, CHST2, TMEM45A, BCAN, ATF3, CAV1, AMPD3, GPC3, NDST1, IRS2, SAP30, GAA, SDC4, STBD1, IER3, PKLR, IGFBP1, PLAUR, CAVIN3, CCN5, LARGE1, NOCT, S100A4, RRAGD, ZFP36, EGFR, EDN2, IDS, CDKN1A, RORA, DUSP1, MIF, PPP1R3C, DPYSL4, KDELR3, DTNA, ADORA2B, HS3ST1, CAVIN1, NR3C1, KLF6, GPC4, CCN1, TNFAIP3, CA12, HEXA, BGN, PPP1R15A, PGM2, PIM1, PRDX5, NAGK, CDKN1B, BRS3, TKTL1, MT1E, ATP7A, MT2A, SDC3, TIPARP, PKP1, ANXA2, PGAM2, DDIT3, PRKCA, SLC37A4, CXCR4, EFNA3, CP, KLF7, CCN2, CHST3, TPD52, LXN, B4GALNT2, PPARGC1A, BCL2, GCNT2, HAS1, KLHL24, SCARB1, SLC25A1, SDC2, CASP6, VHL, FOXO3, PDGFB, B3GALT6, SLC2A5, SRPX, EFNA1, GLRX, ACKR3, PAM, TGFBI, DCN, SIAH2, PLAC8, FBP1, TPST2, PHKG1, MYH9, CDKN1C, GRHPR, PCK1, INHA, HSPA5, NDST2, NEDD4L, TPBG, XPNPEP1, IL6, SLC6A6, MAP3K1, LDHC, AKAP12, TES, KIF5A, LALBA, COL5A1, GPC1, HDLBP, ILVBL, NCAN, TGM2, ETS1, HOXB9, SELENBP1, FOSL2, SULT2B1, TGFB3
**Cholesterol Homeostasis**	FDPS, CYP51A1, IDI1, FDFT1, DHCR7, SQLE, HMGCS1, NSDHL, LSS, MVD, LDLR, TM7SF2, ALDOC, EBP, SCD, PMVK, MVK, LPL, SC5D, FADS2, HMGCR, HSD17B7, ANXA13, SREBF2, PCYT2, ACSS2, ATF3, ADH4, ETHE1, ECH1, CBS, GUSB, FASN, LGALS3, ATF5, ANXA5, TP53INP1, CHKA, GSTM2, ACAT2, AVPR1A, PLSCR1, CLU, ERRFI1, TRIB3, CXCL16, TNFRSF12A, ACTG1, JAG1, LGMN, FBXO6, GPX8, PNRC1, ANTXR2, MAL2, CD9, PPARG, GLDC, STX5, STARD4, CTNNB1, TMEM97, NIBAN1, PDK3, PLAUR, SEMA3B, GNAI1, ABCA2, ATXN2, NFIL3, ALCAM, FABP5, S100A11, CPEB2
**Mitotic Spindle**	ARHGEF2, CLASP1, KIF11, KIF23, ALS2, ARF6, MYO9B, MYH9,

	TUBGCP3, CKAP5, RACGAP1, PREX1, ARHGEF3, NUMA1, SPTBN1, KIF2C, KIF5B, TTK, APC, CEP250, PAFAH1B1, CDC42EP2, KIF3B, CCDC88A, KIF15, BIRC5, CNTROB, NF1, TSC1, CDC27, CNTRL, TUBGCP5, KIF4A, MAPRE1, FGD6, BRCA2, PLK1, RASA1, NEK2, WASF1, TOP2A, TRIO, ARHGEF11, KATNB1, PRC1, ANLN, CDK5RAP2, SMC3, TUBGCP2, ESPL1, MAP1S, ARHGAP27, DOCK4, SAC3D1, INCENP, ARHGAP5, GSN, NET1, LRPPRC, RABGAP1, FLNB, ARAP3, FGD4, ABR, CENPJ, NCK1, CENPF, KIF20B, WASF2, MYH10, CENPE, CYTH2, MID1, KLC1, MARK4, ARL8A, CEP57, UXT, ARHGAP4, CDC42EP4, SASS6, LATS1, OPHN1, WASL, MAP3K11, SOS1, ABL1, TIAM1, KIF1B, ITSN1, SHROOM1, NCK2, DLGAP5, BCR, RANBP9, EPB41L2, SEPTIN9, KATNA1, TUBD1, TPX2, KIF3C, SHROOM2, TUBGCP6, SPTAN1, PALLD, ARFIP2, KNTC1, HOOK3, SUN2, MID1IP1, DLG1, FLNA, ARHGAP10, CDC42BPA, ARHGEF12, MARCKS, NIN, PCM1, RALBP1, EZR, SSH2, CDK1, RAB3GAP1, FARP1, SYNPO, RHOF, ECT2, EPB41, RAPGEF6, KIF22, PCGF5, STAU1, ARHGDIA, AURKA, FBXO5, NUSAP1, PXN, BCAR1, CD2AP, AKAP13, HDAC6, CEP131, LLGL1, ATG4B, PCNT, ALMS1, KPTN, DST, KIFAP3, PPP4R2, ARHGAP29, RASAL2, CCNB2, PIF1, RASA2, NEDD9, SMC1A, ACTN4, PKD2, CLIP2, CDC42, NDC80, TLK1, TAOK2, TBCD, CAPZB, DOCK2, CTTN, SMC4, ARFGEF1, TUBA4A, LMNB1, ABI1, ROCK1, CEP192, BUB1, GEMIN4, PLEKHG2, RICTOR, BCL2L11, DYNC1H1, FSCN1, ARHGEF7, DYNLL2, MYO1E, BIN1, NOTCH2, RFC1, PDLIM5, RHOT2, SORBS2, CDC42EP1, VCL, CLIP1, STK38L, YWHAE, RAPGEF5, CEP72, CSNK1D
**Wnt Beta Catenin Signaling**	MYC, CTNNB1, JAG2, NOTCH1, DLL1, AXIN2, PSEN2, FZD1, NOTCH4, LEF1, AXIN1, NKD1, WNT5B, CUL1, JAG1, MAML1, KAT2A, GNAI1, WNT6, PTCH1, NCOR2, DKK4, HDAC2, DKK1, TCF7, WNT1, NUMB, ADAM17, DVL2, PPARD, NCSTN, HDAC5, CCND2, FRAT1, CSNK1E, RBPJ, FZD8, TP53, SKP2, HEY2, HEY1, HDAC11
**Tgf Beta Signaling**	TGFBR1, SMAD7, TGFB1, SMURF2, SMURF1, BMPR2, SKIL, SKI, ACVR1, PMEPA1, NCOR2, SERPINE1, JUNB, SMAD1, SMAD6, PPP1R15A, TGIF1, FURIN, SMAD3, FKBP1A, MAP3K7, BMPR1A, CTNNB1, HIPK2, KLF10, BMP2, ENG, APC, PPM1A, XIAP, CDH1, ID1, LEFTY2, CDKN1C, TRIM33, RAB31, TJP1, SLC20A1, CDK9, ID3, NOG, ARID4B, IFNGR2, ID2, PPP1CA, SPTBN1, WWTR1, BCAR3, THBS1, FNTA, HDAC1, UBE2D3, LTBP2, RHOA
**Il6 Jak Stat3 Signaling**	IL4R, IL6ST, STAT1, IL1R1, CSF2RB, SOCS3, STAT3, OSMR, IL2RG, IFNGR1, TYK2, IL13RA1, TLR2, IFNGR2, IL10RB, IL6, IL1R2, IL3RA, IFNAR1, TNFRSF1A, MYD88, ACVR1B, CSF3R, ITGB3, REG1A, CXCL1, A2M, CSF2RA, IL15RA, IRF9, PDGFC, HAX1, BAK1, EBI3, INHBE, CRLF2, TNFRSF1B, CD14, PTPN1, PTPN2, IL1B, CSF1, IL18R1, TNF, PF4, CXCL13, LTBR, FAS, IL17RA, CXCL10, IL9R, STAM2, TNFRSF12A, STAT2, HMOX1, LEPR, CBL, CD9, CXCL3, TGFB1, MAP3K8, ITGA4, CD38, JUN, SOCS1, ACVRL1, PIM1, TNFRSF21, PIK3R5, GRB2, IRF1, DNTT, CSF2, IL2RA, PTPN11, IL12RB1, CCR1, CNTFR, PLA2G2A, CXCL9, CD44, IL7, CXCL11, CCL7, LTB, IL17RB, CD36
**Dna Repair**	POLR2H, POLR2A, POLR2G, POLR2E, POLR2J, POLR2F, POLR2C, POLR2K, GTF2H3, POLR2D, ERCC3, DDB2, POLR1C, XPC, PCNA, POLR2I, SUPT4H1, POLD3, POLR3GL, POLR3C, GTF2B, POLR1D, NCBP2, NELFE, GTF2F1, ERCC5, LIG1, ERCC1, ERCC4, POLD4, NELFB, RFC2, ELL, TAF10, RRM2B, SUPT5H, RPA3, SNAPC5, SSRP1, RFC3, RPA2, ELOA, TAF12, NELFCD, TAF13, TAF6, TAF9, GTF2A2, VPS37D, NME1, RNMT, ERCC2, POLE4, VPS37B, NT5C3A, SNAPC4, AAAS, POLR1H, RFC4, ITPA, POM121, BRF2, RFC5,

	SAC3D1, CLP1, NME4, PRIM1, VPS28, TSG101, USP11, TAF1C, TARBP2, POLH, CETN2, POLD1, CANT1, PDE4B, DGCR8, RAD51, SURF1, PNP, ADA, NME3, GTF3C5, NT5C, AK1, GTF2H1, HCLS1, APRT, ERCC8, IMPDH2, POLB, SDCBP, SF3A3, DAD1, UPF3B, GUK1, TP53, ADRM1, SEC61A1, POLA2, FEN1, ZNF707, NUDT9, PDE6G, TYMS, BCAP31, DDB1, NFX1, RAD52, ADCY6, ARL6IP1, DGUOK, POLL, SMAD5, MPG, DUT, POLA1, AGO4, RALA, ZWINT, BCAM, TK2, CSTF3, GTF2H5, HPRT1, BOLA2, GPX4, MPC2, CDA, ALYREF, MRPL40, NPR2, REV3L, EDF1, GSDME, TMED2, STX3, RAE1, UMPS, EIF1B, AK3, NUDT21, RBX1, SRSF6, GMPR2, DCTN4, COX17, CMPK2, CCNO
**G2M Checkpoint**	AURKA, CCNA2, TOP2A, CCNB2, CENPA, BIRC5, CDC20, PLK1, TTK, PRC1, NDC80, KIF11, NUSAP1, CKS2, KIF2C, MKI67, AURKB, TPX2, SMC4, BUB1, CENPF, RACGAP1, CENPE, KIF23, UBE2C, MCM6, MCM3, PTTG1, CDK1, KIF4A, ESPL1, MAD2L1, NEK2, KIF22, HMMR, KPNA2, CDKN3, CDC25A, H2AX, CDC25B, PLK4, CDC6, CCNF, MCM5, LMNB1, E2F3, KIF15, CHEK1, UBE2S, NSD2, HMGB3, DBF4, TACC3, MCM2, CDKN2C, CDKN1B, FANCC, NASP, STAG1, GINS2, FBXO5, POLQ, EZH2, RAD21, STMN1, SUV39H1, PRIM2, E2F1, CHAF1A, NOLC1, GSPT1, BUB3, SMC1A, ILF3, CDC7, INCENP, CKS1B, EXO1, H2AZ1, TFDP1, CCND1, KPNB1, JPT1, LBR, HUS1, KIF20B, TOP1, PDS5B, SRSF1, STIL, ABL1, DTYMK, CDC27, BARD1, ATF5, CDC45, ODC1, XPO1, SFPQ, TMPO, PML, BRCA2, CTCF, KNL1, KMT5A, SLC38A1, TRA2B, MYBL2, TROAP, TENT4A, CUL3, MAPK14, H2BC12, MYC, AMD1, CBX1, CHMP1A, DKC1, YTHDC1, CCNT1, TGFB1, ATRX, LIG3, NUP50, SLC7A5, RBL1, NUMA1, RAD54L, EFNA5, PRPF4B, UCK2, ARID4A, CUL1, UPF1, DR1, MNAT1, SMC2, RBM14, RPA2, SQLE, ORC6, CDK4, POLE, RASAL2, HOXC10, RPS6KA5, CUL4A, SLC7A1, FOXN3, HMGA1, SS18, TRAIP, PRMT5, CUL5, DDX39A, MARCKS, PBK, ORC5, SAP30, KATNA1, HNRNPD, POLA2, HIRA, HIF1A, SYNCRIP, TLE3, NCL, RAD23B, E2F2, EGF, HMGN2, SRSF10, SNRPD1, CASP8AP2, SMARCC1, SLC12A2, NOTCH2, TNPO2, SMAD3, MAP3K20, HSPA8, G3BP1, PTTG3P, DMD, MEIS1, HNRNPU, SRSF2, MT2A, NUP98, EWSR1, KIF5B, MTF2, E2F4, BCL3, PURA, MEIS2, PAFAH1B1, WRN, H2AZ2, ODF2
**Apoptosis**	CASP3, CASP9, DFFA, CASP7, CFLAR, BIRC3, PMAIP1, CASP8, JUN, BCL2L11, MCL1, IL1B, SPTAN1, DIABLO, BAX, BIK, IL1A, BID, CDKN1A, GADD45A, DDIT3, CDKN1B, TNF, GSN, TNFSF10, CASP6, SQSTM1, FASLG, EGR3, CD44, FAS, IL18, IGFBP6, PRF1, DAP, CCND1, BTG3, F2R, SATB1, BNIP3L, CASP4, TNFRSF12A, CREBBP, RHOB, GPX3, PDGFRB, TSPO, CCND2, XIAP, TIMP1, CTNNB1, IRF1, HSPB1, ADD1, TIMP2, BTG2, TIMP3, LEF1, CASP1, GPX1, BCL10, IGF2R, CDC25B, AIFM3, CD38, PPP3R1, HGF, CLU, ATF3, LGALS3, LUM, LMNA, GADD45B, CDK2, IFNB1, RETSAT, SMAD7, SOD1, PTK2, ENO2, HMOX1, IER3, BCL2L10, CD2, GCH1, MMP2, VDAC2, TAP1, PLAT, IFNGR1, APP, BRCA1, ROCK1, PSEN1, DCN, PSEN2, SOD2, BMF, EREG, KRT18, TGFB2, RELA, WEE1, RARA, CD14, CD69, PEA15, DNAJC3, CASP2, CTH, PLCB2, BMP2, HMGB2, PLPPR4, H1-0, TGFBR3, EBP, TXNIP, ANKH, RHOT2, CYLD, GSTM1, GSR, BGN, BCL2L1, GNA15, MGMT, PPT1, F2, IL6, SC5D, IFITM3, RNASEL, EMP1, CAV1, DNM1L, ANXA1, TOP2A, ISG20, SLC20A1, MADD, PPP2R5B, BCAP31, ERBB3, NEDD9, SAT1, PDCD4, BCL2L2, FEZ1, ERBB2, DNAJA1, DAP3, DPYD, NEFH, PAK1, FDXR, GPX4, ETF1, CCNA1, GUCY2D, AVPR1A
**Notch Signaling**	JAG1, NOTCH3, NOTCH2, APH1A, HES1, CCND1, FZD1, PSEN2, FZD7, DTX1, DLL1, FZD5, MAML2, NOTCH1, PSENEN, WNT5A, CUL1, WNT2, DTX4, SAP30, PPARD, KAT2A, HEYL, SKP1, RBX1,

	TCF7L2, ARRB1, LFNG, PRKCA, DTX2, ST3GAL6, FBXW11
**Adipogenesis**	FABP4, ADIPOQ, PPARG, LIPE, DGAT1, LPL, CPT2, CD36, GPAM, ADIPOR2, ACAA2, ETFB, ACOX1, ACADM, HADH, IDH1, SORBS1, ACADS, UCK1, SCP2, DECR1, CDKN2C, TALDO1, TST, MCCC1, PGM1, REEP5, BCL2L13, SLC25A10, ME1, PHYH, PIM3, YWHAG, NDUFAB1, GPD2, ADIG, ECHS1, QDPR, CS, ECH1, SLC25A1, ACADL, TOB1, GRPEL1, CRAT, GBE1, CAVIN2, SCARB1, PEMT, CHCHD10, AK2, APOE, UQCR10, TANK, ANGPTL4, ACO2, FAH, ACLY, IFNGR1, SLC5A6, JAGN1, EPHX2, IDH3G, GPX3, ELMOD3, ORM1, RETSAT, ESRRA, HIBCH, SUCLG1, STAT5A, ITGA7, MRAP, PLIN2, CYC1, ALDH2, RNF11, ALDOA, SULT1A1, DDT, SDHB, CD151, SLC27A1, BCKDHA, C3, LEP, ADCY6, ELOVL6, LTC4S, SPARCL1, RMDN3, MTCH2, SOWAHC, SLC1A5, CMPK1, REEP6, NDUFA5, FZD4, DRAM2, MGST3, ATP1B3, RETN, STOM, ESYT1, GHITM, DNAJC15, GADD45A, VEGFB, PFKL, COQ3, NABP1, CYP4B1, PPM1B, ARAF, CAVIN1, COL4A1, IMMT, DHRS7, COL15A1, NMT1, COQ5, LAMA4, AGPAT3, BAZ2A, IDH3A, LIFR, PREB, PTGER3, GPHN, PFKFB3, GPX4, SSPN, SQOR, MTARC2, DLD, ITIH5, CD302, ATL2, GPAT4, LPCAT3, TKT, UQCRC1, CAT, OMD, DLAT, MRPL15, RIOK3, RTN3, CHUK, G3BP2, SDHC, SAMM50, ARL4A, SNCG, PDCD4, COQ9, APLP2, SOD1, PTCD3, PHLDB1, ENPP2, HSPB8, AIFM1, CCNG2, PPP1R15B, MDH2, ABCA1, COX7B, MYLK, COX8A, DHRS7B, MIGA2, MGLL, ITSN1, DHCR7, RREB1, CMBL, UBC, ATP5PO, PRDX3, DBT, NDUFS3, NKIRAS1, RAB34, CIDEA, UQCRQ, PEX14, BCL6, COX6A1, DNAJB9, MAP4K3, ANGPT1, UBQLN1, NDUFB7, SLC19A1, ABCB8, SLC66A3, POR, UCP2, UQCR11
**Estrogen Response Early**	GREB1, CA12, SLC9A3R1, MYB, ANXA9, IGFBP4, SYBU, NPY1R, PDZK1, NRIP1, MLPH, HSPB8, EGR3, KRT19, LRIG1, KDM4B, PGR, RHOBTB3, TPD52L1, ELOVL2, RET, TPBG, TFF1, MAPT, SCNN1A, ABAT, FLNB, XBP1, CELSR2, RAB31, MYBL1, MREG, FAM102A, MSMB, STC2, RETREG1, SIAH2, ZNF185, SLC19A2, SLC1A4, FHL2, BCL2, PMAIP1, AREG, OVOL2, TSKU, ADCY9, RASGRP1, MUC1, KAZN, SLC27A2, FKBP4, CXCL12, TMPRSS3, RARA, IL17RB, CBFA2T3, TFF3, UGCG, CCND1, SLC22A5, WFS1, PTGES, WWC1, CCN5, MYC, ITPK1, TMEM164, ARL3, MED13L, SEMA3B, KRT18, SLC16A1, TJP3, SLC26A2, FCMR, SULT2B1, SNX24, TFAP2C, TTC39A, GJA1, PRSS23, OLFM1, RAPGEFL1, ASB13, TIPARP, ABCA3, FRK, DHRS2, AQP3, KCNK15, TGIF2, FOXC1, ELF3, REEP1, PEX11A, PODXL, KLF4, BAG1, CELSR1, PLAAT3, SLC7A5, MPPED2, TIAM1, CLDN7, MYOF, RBBP8, OLFML3, GFRA1, FARP1, SVIL, TGM2, DEPTOR, CYP26B1, PAPSS2, SLC1A1, DLC1, JAK2, AFF1, KLK10, P2RY2, BLVRB, CISH, GLA, ADD3, PDLIM3, MINDY1, FOS, KRT8, SLC37A1, B4GALT1, CALCR, ESRP2, IGF1R, NBL1, SFN, OPN3, ABHD2, AR, SLC39A6, SYT12, CD44, MED24, BCL11B, CANT1, KRT13, KRT15, TOB1, SLC7A2, LAD1, TUBB2B, TBC1D30, SEC14L2, ENDOD1, HR, SCARB1, NCOR2, RHOD, INPP5F, PPIF, DHRS3, FDFT1, GAB2, UNC119, KLF10, HES1, FKBP5, SLC2A1, AMFR, NADSYN1, INHBB, BHLHE40, CALB2, FASN, CHPT1, MYBBP1A, ELOVL5, DYNLT3, ABLIM1, SOX3, SLC24A3, RAB17, MAST4, KCNK5, ELF1, RPS6KA2, ISG20L2, IL6ST, SYNGR1, SH3BP5, ALDH3B1, THSD4, CLIC3, NXT1, NAV2, RRP12, ADCY1, DHCR7, MICB, AKAP1
**Estrogen Response Late**	TFF1, SLC9A3R1, TPD52L1, PRSS23, CA12, PDZK1, ANXA9, CELSR2, TJP3, PGR, RET, MYB, TPBG, EGR3, ARL3, OLFM1, NPY1R, SCNN1A, XBP1, AREG, IL17RB, NRIP1, ASS1, TFF3, FKBP4, SLC27A2, SEMA3B, GPER1, LLGL2, AGR2, KRT19, CCN5, BLVRB, FLNB, PDCD4, CALCR, IGFBP4, DNAJC12, TIAM1, TSPAN13,

	CXCL12, RAB31, PKP3, CYP26B1, FKBP5, SIAH2, ISG20, TMPRSS3, SERPINA3, WFS1, MAPT, PDLIM3, RBBP8, GJB3, PRLR, SLC1A4, FOS, PLAAT3, SLC7A5, SERPINA5, IMPA2, DHCR7, MYOF, CDH1, EMP2, OVOL2, DLG5, SOX3, CHPT1, KLK10, ELOVL5, RAPGEFL1, JAK2, SLC26A2, SLC22A5, ITPK1, PCP4, PAPSS2, NAB2, FAM102A, BCL2, LSR, CACNA2D2, CA2, ASCL1, ACOX2, CISH, GLA, PTGES, PERP, OPN3, KRT13, HSPB8, UGDH, CLIC3, KLK11, PLAC1, ABHD2, SCARB1, DCXR, CCND1, SFN, ABCA3, SULT2B1, CCNA1, STIL, MICB, ZFP36, CAV1, NBL1, CD44, HR, HOMER2, BTG3, GAL, ETFB, BAG1, FRK, SLC16A1, AFF1, TFAP2C, IGSF1, HPRT1, CDC6, FARP1, AMFR, DHRS2, NXT1, S100A9, SLC29A1, SLC24A3, FOXC1, KIF20A, TOB1, FDFT1, DNAJC1, TPSAB1, GFUS, FGFR3, SGK1, ID2, GALE, BATF, MAPK13, FABP5, MEST, JAK1, CYP4F11, KCNK5, CPE, XRCC3, CXCL14, SCUBE2, CDC20, IL6ST, GINS2, TRIM29, UNC13B, LAMC2, LARGE1, SLC2A8, PLXNB1, PRKAR2B, RPS6KA2, HSPA4L, TFPI2, SERPINA1, TNNC1, HMGCS2, ALDH3A2, CD9, IDH2, SORD, MDK, ALDH3B1, PTGER3, RABEP1, KLF4, PPIF, SNX10, METTL3, PLK4, COX6C, ST14, NCOR2, MOCS2, NMU, TH, RNASEH2A, CHST8, TST, TOP2A, CKB, LTF, DUSP2, PTPN6, ATP2B4, ST6GALNAC2, ADD3, DYNLT3
**Androgen Response**	KLK3, KLK2, ACSL3, PIAS1, CAMKK2, NKX3-1, TMPRSS2, APPBP2, CENPN, BMPR1B, MAF, FADS1, ZBTB10, HMGCR, SPCS3, INSIG1, NGLY1, UBE2J1, ELK4, ABCC4, ELOVL5, ALDH1A3, AZGP1, ABHD2, SAT1, DBI, DHCR24, SORD, STK39, TPD52, IDI1, B2M, MAP7, DNAJB9, FKBP5, HERC3, PGM3, HOMER2, ELL2, UAP1, SEC24D, LMAN1, PMEPA1, INPP4B, RRP12, SELENOP, NDRG1, KRT19, ITGAV, B4GALT1, MAK, SPDEF, GSR, KRT8, LIFR, PLPP1, STEAP4, GUCY1A1, SRP19, CCND3, IQGAP2, RAB4A, SLC26A2, SMS, ZMIZ1, SGK1, MERTK, HPGD, ANKH, GNAI3, SRF, SLC38A2, HMGCS1, ARID5B, CDC14B, TNFAIP8, XRCC5, GPD1L, ACTN1, RPS6KA3, TSC22D1, PDLIM5, TMEM50A, ADAMTS1, NCOA4, AKAP12, SCD, PTK2B, CDK6, ADRM1, H1-0, HSD17B14, VAPA, CCND1, XRCC6, MYL12A, UBE2I, PTPN21, AKT1, PA2G4
**Myogenesis**	ACTA1, TNNI2, MYL1, TNNC1, TNNC2, MYH3, MYLPF, TNNT3, TNNT2, CASQ2, ACTC1, MYOM1, MYL4, MYBPH, MYH7, MYH8, ACTN2, TNNI1, CRYAB, SGCG, HRC, TNNT1, DES, MYOZ1, RYR1, CSRP3, ADAM12, ATP2A1, CKM, SVIL, MYOM2, MYL6B, TPM2, MYL2, CKMT2, BIN1, MYH1, ENO3, FLII, FXYD1, TPM3, DMD, IGFBP3, CHRNB1, SPEG, FHL1, ACTN3, TCAP, MYLK, MYL3, PYGM, LDB3, COX6A2, FABP3, MYL7, ITGB5, CHRNA1, SSPN, COL3A1, KCNH1, GJA5, MYF6, MYH2, MAPK12, PGAM2, MYOG, MYH4, AEBP1, HBEGF, MEF2C, NOS1, CNN3, IGFBP7, CACNA1H, GSN, CACNG1, PPFIA4, MB, SPHK1, SCHIP1, MEF2D, SMTN, CDKN1A, GAA, TPD52L1, HSPB2, SGCA, BDKRB2, COX7A1, COL4A2, PLXNB2, CTF1, COL15A1, KCNH2, AGRN, MYO1C, SIRT2, SGCD, SORBS1, VIPR1, FGF2, FKBP1B, TEAD4, CASQ1, KLF5, PDLIM7, AK1, TAGLN, RIT1, MEF2A, ANKRD2, AKT2, LAMA2, DENND2B, IFRD1, NCAM1, SYNGR2, PICK1, COL6A3, CRAT, DMPK, MYH11, MYH9, IGF1, CKB, FST, GPX3, PVALB, PTP4A3, ITGB1, HSPB8, ACHE, CHRNG, PKIA, NAV2, HDAC5, ATP6AP1, CLU, ACSL1, COL1A1, MRAS, PDE4DIP, AGL, ERBB3, GABARAPL2, CAV3, BHLHE40, PPP1R3C, LARGE1, LPIN1, CDH13, NQO1, PRNP, PSEN2, DAPK2, LSP1, SLC6A8, REEP1, EIF4A2, SH2B1, MYBPC3, ITGA7, SPTAN1, SORBS3, PC, SCD, GNAO1, STC2, CFD, RB1, BAG1, ABLIM1, PTGIS, MAPRE3, OCEL1, SH3BGR, NOTCH1, TSC2, TGFB1, FDPS, CAMK2B, EPHB3, GADD45B, APP, APLNR, ITGB4, SOD3, FOXO4, EFS, PFKM, COL6A2, DTNA, KIFC3, SPARC, SPDEF, SLN, WWTR1, CD36, ADCY9, APOD

**Protein Secretion**	ARCN1, TMED10, COPB2, RAB14, ATP7A, COPB1, LAMP2, EGFR, IGF2R, COPE, PPT1, AP3S1, BET1, CLCN3, AP2S1, CLTC, AP2M1, RER1, KIF1B, ARF1, OCRL, ICA1, MON2, ARFGEF2, TMED2, NAPG, TMX1, PAM, SCAMP1, SH3GL2, RAB2A, COG2, VAMP3, ERGIC3, DOP1A, VAMP4, VPS4B, NAPA, GNAS, STX7, SEC22B, VPS45, STX16, YKT6, SNX2, ARFGEF1, CLTA, CLN5, M6PR, SGMS1, USO1, TSG101, DNM1L, LMAN1, YIPF6, ARFIP1, ANP32E, TSPAN8, DST, SCRN1, SEC31A, VAMP7, SCAMP3, AP2B1, RAB22A, AP3B1, TPD52, ATP6V1H, GBF1, KRT18, CAV2, ATP1A1, BNIP3, ZW10, GALC, ADAM10, RAB5A, GOLGA4, SEC24D, AP1G1, MAPK1, GOSR2, RPS6KA3, STAM, ABCA1, SNAP23, SOD1, GLA, ATP6V1B1, STX12, CD63, TOM1L1, SSPN, ARFGAP3, RAB9A, CTSC
**Interferon Alpha Response**	MX1, ISG15, OAS1, IFIT3, IFI44, IFI35, IRF7, RSAD2, IFI44L, IFITM1, IFI27, IRF9, OASL, EIF2AK2, IFIT2, CXCL10, TAP1, SP110, DDX60, UBE2L6, USP18, PSMB8, IFIH1, BST2, LGALS3BP, ADAR, ISG20, GBP2, IRF1, PLSCR1, PSMB9, HERC6, SAMD9, CMPK2, IFITM3, RTP4, STAT2, SAMD9L, LY6E, IFITM2, HELZ2, CXCL11, TRIM21, PARP14, TRIM26, PARP12, NMI, RNF31, HLA-C, CASP1, TRIM14, TDRD7, DHX58, PARP9, PNPT1, TRIM25, PSME1, WARS1, EPSTI1, UBA7, PSME2, B2M, TRIM5, C1S, LAP3, LAMP3, GBP4, NCOA7, TMEM140, CD74, GMPR, PSMA3, PROCR, IL7, IFI30, IRF2, CSF1, IL15, CNP, TENT5A, IL4R, CMTR1, CD47, LPAR6, MOV10, CASP8, TXNIP, SLC25A28, SELL, TRAFD1, BATF2, RIPK2, CCRL2, NUB1, OGFR, MVB12A, ELF1
**Interferon Gamma Response**	STAT1, ISG15, IFIT1, MX1, IFIT3, IFI35, IRF7, IFIT2, OAS2, TAP1, EIF2AK2, RSAD2, MX2, IRF1, OAS3, TNFSF10, IRF9, CXCL10, IFI44, BST2, XAF1, SP110, OASL, PSMB8, IFI44L, IFITM3, DDX60, LGALS3BP, GBP4, IRF8, PSMB9, PML, IFIH1, UBE2L6, IFI27, ADAR, LY6E, STAT2, CXCL9, IL10RA, PLA2G4A, TRIM21, USP18, PTGS2, EPSTI1, C1S, DDX58, IL15, NLRC5, NMI, IDO1, PSMB10, CXCL11, ITGB7, SAMHD1, HERC6, CMPK2, SAMD9L, RTP4, PTPN2, PARP14, TNFAIP2, IFITM2, PLSCR1, SOCS1, CASP1, ICAM1, WARS1, PSME1, ISG20, IRF2, TRIM14, FCGR1A, MARCHF1, SOCS3, JAK2, HLA-DMA, PARP12, TNFAIP6, TRIM26, VCAM1, CD274, CIITA, NAMPT, SELP, GPR18, FPR1, HELZ2, PSME2, SERPING1, CCL5, RNF31, SOD2, TRIM25, LAP3, PSMA3, RNF213, PELI1, CFB, CD86, TXNIP, HLA-DQA1, GCH1, PNP, CCL7, PTPN6, SPPL2A, IL4R, PNPT1, DHX58, BTG1, CASP8, IFI30, CCL2, FGL2, CASP7, SECTM1, IL15RA, CD40, TRAFD1, HLA-DRB1, GBP6, LCP2, HLA-G, MT2A, RIPK1, KLRK1, UPP1, PSMB2, TDRD7, HIF1A, EIF4E3, VAMP8, PFKP, CD38, ZBP1, BANK1, TOR1B, RBCK1, PDE4B, MVP, IL7, BPGM, CMTR1, AUTS2, B2M, RIPK2, CD69, MYD88, PSMA2, PIM1, NOD1, CFH, TAPBP, SLC25A28, PTPN1, TNFAIP3, SSPN, NUP93, MTHFD2, CDKN1A, IRF4, NFKB1, BATF2, HLA-B, LATS2, IRF5, SLAMF7, ISOC1, P2RY14, STAT3, NCOA3, HLA-A, IL6, GZMA, IFNAR2, CD74, RAPGEF6, CASP4, FAS, OGFR, ARL4A, SRI, LYSMD2, CSF2RB, ST3GAL5, C1R, CASP3, CMKLR1, NFKBIA, METTL7B, ST8SIA4, XCL1, IL2RB, VAMP5, IL18BP, ZNFX1, ARID5B, APOL6, STAT4
**Apical Junction**	ACTN1, CLDN7, ACTN3, CLDN19, DLG1, TJP1, COL17A1, NECTIN1, CLDN9, CDH3, CTNND1, CDH1, LIMA1, VCL, SHROOM2, NECTIN3, NECTIN2, CLDN14, PTPRC, THY1, VASP, ITGB1, ZYX, ACTN4, CLDN4, CADM3, ITGA3, NF2, B4GALT1, NEXN, CDH6, ADAM15, PTK2, HADH, LAMB3, CLDN11, CDH4, IRS1, SORBS3, CLDN8, EVL, CLDN15, THBS3, NECTIN4, JAM3, CDH11, ITGB4, RASA1, AKT2, TMEM8B, LAMA3, RSU1, ICAM1, CLDN18, GTF2F1, TSC1, LAMC2, NRAP, CTNNA1, CD99, FSCN1, COL16A1, GNAI2, TGFBI, PALS1, JUP, ACTN2, INSIG1, LAYN, PFN1, CNN2, TAOK2, AMIGO2, MDK,

	INPPL1, CLDN6, ARPC2, SRC, PBX2, MAPK14, MAP4K2, PTEN, CALB2, CNTN1, TSPAN4, VCAN, CDH8, ATP1A3, EPB41L2, MYL9, SPEG, ADRA1B, SYK, CADM2, ALOX15B, CX3CL1, MYH10, DMP1, CDSN, MAPK13, MYL12B, RAC2, FBN1, DSC1, CD34, ICAM4, MSN, MAPK11, ACTC1, TRAF1, CRB3, ITGA2, ITGA10, BAIAP2, ARHGEF6, ACTG1, MVD, PDZD3, ADAM9, PCDH1, SLIT2, KRT31, SGCE, GNAI1, PIK3CB, NLGN2, YWHAH, PARVA, CRAT, ADAMTS5, AMH, EGFR, ICAM2, CD274, CDH15, SHC1, MPZL2, WNK4, NFASC, PIK3R3, SDC3, SIRPA, GRB7, FLNC, PARD6G, COL9A1, NRXN2, MAP3K20, SLC30A3, RHOF, BMP1, MMP2, ICAM5, VWF, MYH9, CD209, VCAM1, DHX16, ACTG2, NEGR1, NF1, ADAM23, PLCG1, KCNH2, CERCAM, NRTN, IKBKG, DSC3, ACTB, TUBG1, PPP2R2C, HRAS, MMP9, TNFRSF11B, PKD1, CDK8, FYB1, AMIGO1, CD276, GAMT, NLGN3, SKAP2, RRAS, CAP1, EXOC4, ACTA1, TIAL1, LDLRAP1, VAV2, AKT3, SYMPK, STX4, PECAM1, MADCAM1, ITGA9, CD86, TRO, CLDN5, MPZL1, WASL
**Apical Surface**	B4GALT1, RHCG, MAL, LYPD3, PKHD1, ATP6V0A4, CRYBG1, SHROOM2, SRPX, MDGA1, TMEM8B, THY1, PCSK9, EPHB4, DCBLD2, GHRL, LYN, GAS1, FLOT2, PLAUR, AKAP7, ATP8B1, EFNA5, SLC34A3, APP, GSTM3, HSPB1, SLC2A4, IL2RB, RTN4RL1, NCOA6, SULF2, ADAM10, BRCA1, GATA3, AFAP1L2, IL2RG, CD160, ADIPOR2, SLC22A12, NTNG1, SCUBE1, CX3CL1, CROCC
**Hedgehog Signaling**	SHH, PTCH1, NRCAM, NRP1, SCG2, AMOT, UNC5C, ADGRG1, HEY1, GLI1, THY1, SLIT1, CDK6, HEY2, NRP2, TLE3, TLE1, L1CAM, PLG, NKX6-1, NF1, RASA1, ETS2, RTN1, CRMP1, MYH9, VEGFA, CELSR1, CNTFR, ACHE, PML, CDK5R1, VLDLR, OPHN1, LDB1, DPYSL2
**Complement**	C2, C1S, CFB, C1R, SERPINE1, MMP14, SERPING1, CTSL, F5, MMP13, F7, CTSS, LGMN, PLG, C1QA, CASP1, GZMA, ADAM9, CALM3, C1QC, TIMP1, DPP4, KLK1, KLKB1, CD59, CR2, MMP15, LAP3, SPOCK2, F10, CTSB, SERPINA1, CTSO, CD40LG, CBLB, PDP1, C4BPB, PLEK, GP9, PLAUR, C3, F2, CASP4, STX4, CTSC, USP15, CR1, DUSP6, SERPINB2, GPD2, CFH, FN1, CD36, CA2, PSMB9, APOBEC3G, FCN1, GZMK, PDGFB, CLU, CASP10, LRP1, CTSD, S100A9, WAS, BRPF3, PLAT, CDA, MT3, CASP7, PRSS36, PFN1, GZMB, RNF4, ZEB1, CASP5, IRF1, CPQ, CDK5R1, ATOX1, PIK3CA, TMPRSS6, CPM, RCE1, CALM1, DOCK9, KYNU, RASGRP1, USP14, LCP2, GP1BA, KIF2A, GNB4, LCK, OLR1, PREP, MSRB1, LTA4H, ZFPM2, LYN, ACTN2, SIRT6, APOC1, PRKCD, ITGAM, DGKH, LTF, MAFF, KCNIP2, PCLO, DOCK10, SH2B3, RABIF, SRC, HPCAL4, CD46, PRDM4, GNAI3, C9, PPP2CB, IRF2, FYN, JAK2, PLA2G4A, PRCP, USP8, RHOG, L3MBTL4, LAMP2, PIM1, CXCL1, F3, GNAI2, CASP9, XPNPEP1, PLSCR1, IRF7, CD55, HSPA5, GNB2, DYRK2, PLA2G7, S100A12, GRB2, PHEX, GNGT2, DOCK4, MMP12, KCNIP3, FDX1, TIMP2, MMP8, FCER1G, RBSN, ANXA5, CTSV, GCA, EHD1, PRSS3, COL4A2, CSRP1, PIK3R5, SERPINC1, ANG, APOBEC3F, GATA3, DUSP5, CASP3, USP16, CP, PSEN1, LIPA, PCSK9, DGKG, GNG2, ME1, GMFB, SCG3, PPP4C, CCL5, CTSH, F8, APOA4, IL6, AKAP10, ERAP2, VCPIP1, HSPA1A, RAF1, NOTCH4, ADRA2B, CEBPB, HNF4A, LGALS3, TNFAIP3, CDH13, ITIH1, TFPI2, PIK3CG, S100A13
**Unfolded Protein Response**	ATF4, HERPUD1, PARN, EXOSC4, HSP90B1, XBP1, EXOSC9, HSPA5, ATF3, EXOSC1, EXOSC5, ASNS, ARFGAP1, WFS1, SRPRB, DNAJB9, TSPYL2, NFYA, CALR, KHSRP, DNAJC3, EXOSC2, NOP14, ATF6, HYOU1, RRP9, NOP56, CXXC1, IGFBP1, CNOT4, DCP2, PDIA6, ATP6V0D1, PREB, SEC31A, GOSR2, SERP1, NFYB, NHP2, DCTN1, SLC7A5, SSR1, EDEM1, EDC4, EIF2AK3, MTHFD2, SRPRA, NOLC1, ERN1, FKBP14, ZBTB17, WIPI1, YIF1A, CCL2, DKC1, EIF4EBP1,

	EIF4A1, EIF2S1, TATDN2, XPOT, LSM4, CHAC1, STC2, PDIA5, NABP1, LSM1, SDAD1, EIF4A2, EIF4E, IARS1, DDX10, GEMIN4, TARS1, ALDH18A1, DDIT4, HSPA9, SEC11A, EIF4A3, POP4, ERO1A, CEBPG, BAG3, KDELR3, CEBPB, CNOT2, SHC1, SPCS3, H2AX, SLC1A4, RPS14, NPM1, CNOT6, CKS1B, PSAT1, EIF4G1, YWHAZ, SLC30A5, MTREX, KIF5B, DCP1A, IFIT1, IMP3, SPCS1, DNAJA4, VEGFA, TTC37, TUBB2A, FUS, EXOSC10, EEF2, EXOC2, PAIP1, BANF1
**Pi3K Akt Mtor Signaling**	MAPK8, PIK3R3, GRB2, NFKBIB, MAP2K6, MAPK9, AKT1, MAPK1, PLCG1, TRIB3, GSK3B, MAP2K3, CDKN1A, RAC1, RIPK1, AKT1S1, ACTR2, PRKAR2A, YWHAB, HRAS, PDK1, PIKFYVE, TBK1, ACTR3, E2F1, MYD88, ITPR2, SQSTM1, RPS6KA1, PTPN11, MAPKAP1, PLCB1, RAF1, CAMK4, RPTOR, CFL1, CDK4, TRAF2, GNGT1, UBE2N, ADCY2, CDKN1B, VAV3, FGF6, ECSIT, RALB, ARF1, MKNK1, CDK1, PTEN, ARHGDIA, GRK2, FGF17, DDIT3, IRAK4, TIAM1, CDK2, SFN, PRKCB, GNA14, EIF4E, CLTC, TSC2, FGF22, PPP1CA, DUSP3, HSP90B1, IL4, STAT2, SLA, EGFR, PLA2G12A, MAPK10, CALR, THEM4, RIT1, MKNK2, PPP2R1B, CAB39L, ARPC3, PITX2, NCK1, IL2RG, PFN1, FASLG, NOD1, DAPP1, UBE2D3, CAB39, AP2M1, MAP3K7, PRKAG1, CSNK2B, PRKAA2, ATF1, SLC2A1, PIN1, TNFRSF1A, LCK, RPS6KA3, NGF, CXCR4, ACACA, SMAD2, PAK4
**Mtorc1 Signaling**	FADS1, DDIT4, CALR, HK2, PGK1, SLC7A5, CTSC, ACSL3, SLC1A5, M6PR, TFRC, DDIT3, TMEM97, IFRD1, PLOD2, TUBA4A, PSAT1, CORO1A, LDHA, MTHFD2, FADS2, VLDLR, WARS1, SCD, P4HA1, ACTR2, IDH1, SLC2A1, GBE1, SERPINH1, NUPR1, PSMG1, PSPH, NAMPT, CDKN1A, BHLHE40, HSPA9, HSPA5, EGLN3, LGMN, PNP, XBP1, SLA, DDX39A, HSPE1, ACLY, SLC7A11, SSR1, GLA, SQSTM1, PDK1, PSMC2, PRDX1, SERP1, TRIB3, NFIL3, HMGCS1, GOT1, TPI1, ELOVL6, ASNS, PSMD14, PSMA4, PPA1, HPRT1, AURKA, HMGCR, GAPDH, DHFR, DHCR7, IMMT, UCHL5, YKT6, INSIG1, SQLE, IGFBP5, IFI30, CYP51A1, FGL2, ENO1, IDI1, CYB5B, SHMT2, TXNRD1, G6PD, SLC9A3R1, RAB1A, EBP, PNO1, PIK3R3, ACTR3, LDLR, SLC2A3, UBE2D3, ELOVL5, CACYBP, EDEM1, ATP6V1D, TES, TM7SF2, PSMA3, ITGB2, AK4, SLC1A4, TOMM40, SLC6A6, PPIA, ADD3, ME1, CCNF, SLC37A4, ALDOA, BTG2, UFM1, CCNG1, STC1, NMT1, PSMC6, FDXR, RRM2, DHCR24, PSMC4, CTH, PSME3, CFP, POLR3G, ACACA, QDPR, MCM2, PSMD12, SC5D, CANX, RPN1, HSPA4, NIBAN1, TBK1, SEC11A, BCAT1, PSMB5, PSMD13, PGM1, PLK1, GLRX, COPS5, ETF1, GSK3B, NUP205, SORD, PHGDH, GMPS, RRP9, EEF1E1, LTA4H, SDF2L1, FKBP2, RDH11, CXCR4, MLLT11, GCLC, TCEA1, MAP2K3, HSPD1, SYTL2, MCM4, PPP1R15A, USO1, NFKBIB, UNG, GTF2H1, RPA1, HSP90B1, ERO1A, GSR, PITPNB, EPRS1, SRD5A1, TUBG1, MTHFD2L, ADIPOR2, NUFIP1, CDC25A, PDAP1, STARD4, BUB1, ARPC5L, GPI, EIF2S2, CD9, ATP2A2, GGA2, HMBS, RIT1, SKAP2, STIP1, DAPP1, ABCF2, NFYC, ATP5MC1, PFKL, CCT6A
**E2F Targets**	AURKA, BRCA2, CCP110, CENPE, CKS2, DCLRE1B, DNMT1, DONSON, EED, GINS1, GINS4, H2AZ1, LIG1, MAD2L1, MCM2, MCM4, MCM5, MCM7, MELK, MMS22L, NAA38, NASP, NUDT21, NUP205, ORC6, PCNA, PLK4, POLE, PRIM2, RAD51AP1, RFC2, RPA2, RPA3, SUV39H1, TMPO, UBE2T, WDR90, CDK1, MCM3, TOP2A, MCM6, BIRC5, CCNB2, RRM2, HMGB2, BUB1B, RFC3, EZH2, CHEK1, SMC4, MKI67, CDC20, PLK1, KIF2C, DLGAP5, AURKB, CDC25A, TRIP13, H2AX, HMMR, E2F8, BRCA1, MYBL2, POLD1, RACGAP1, CKS1B, KPNA2, MSH2, CDKN3, ATAD2, RPA1, STMN1, TIPIN, TK1, CDCA8, ESPL1, NCAPD2, RANBP1, MRE11, KIF4A, LMNB1, KIF22, UNG, SMC1A, CCNE1, CDCA3, ASF1B,

	POLA2, TIMELESS, HELLS, UBE2S, PRKDC, RAN, USP1, SPAG5, POLD3, DUT, TACC3, KIF18B, CDC25B, SRSF1, GINS3, NOLC1, SLBP, CHEK2, SPC25, BARD1, DCTPP1, SMC3, RNASEH2A, DEK, CENPM, RAD51C, CBX5, RFC1, POLD2, DSCC1, ILF3, DEPDC1, DCK, CDKN2C, MYC, TCF19, RAD1, LBR, NBN, PTTG1, UBR7, POLE4, TUBG1, CTCF, CNOT9, TUBB, SMC6, ZW10, PA2G4, SSRP1, NAP1L1, ANP32E, HMGB3, IPO7, RAD21, CDK4, CDKN1A, BRMS1L, CTPS1, RAD50, TRA2B, CSE1L, PAICS, STAG1, LUC7L3, PPM1D, NME1, SRSF2, XPO1, HNRNPD, PMS2, ASF1A, EXOSC8, MLH1, NUP107, ORC2, TP53, TFRC, HMGA1, PSIP1, DDX39A, SNRPB, CDKN1B, MTHFD2, WEE1, PRDX4, PHF5A, TBRG4, SHMT1, PRPS1, DIAPH3, NUP153, PSMC3IP, XRCC6, PNN, HUS1, RBBP7, PDS5B, NOP56, MXD3, PPP1R8, GSPT1, CDKN2A, AK2, CIT, ING3, JPT1, POP7, SYNCRIP, EIF2S1, LYAR, PAN2, SPC24
**Myc Targets V1**	PCNA, PSMD8, PSMD7, SET, SNRPA1, RAN, SRSF2, G3BP1, STARD7, NPM1, BUB3, EIF3D, XPO1, FBL, EIF4A1, CANX, NAP1L1, CBX3, CCT3, C1QBP, U2AF1, UBE2L3, SSBP1, SRSF1, TCP1, MCM2, EIF3B, PSMD14, SNRPA, PWP1, APEX1, TXNL4A, HNRNPR, PSMB2, HPRT1, MCM6, NME1, SNRPD1, EEF1B2, HSPD1, CAD, RPL18, PGK1, DDX18, RPS2, LDHA, RUVBL2, RNPS1, EIF2S1, RANBP1, MCM5, IARS1, UBE2E1, MYC, AP3S1, RFC4, DUT, PSMA4, RPS3, SNRPG, PHB2, SSB, EIF4H, SRSF3, CCT4, TFDP1, EIF2S2, CDK4, SNRPD3, PSMD1, RACK1, GOT2, PABPC4, CCT5, PRPS2, ACP1, PRPF31, CCT2, KARS1, DEK, EIF4G2, XPOT, SRM, UBA2, KPNB1, PSMA7, TRA2B, COX5A, PA2G4, PCBP1, HNRNPA1, CCNA2, PPIA, EIF3J, ORC2, PSMA2, SYNCRIP, HDAC2, LSM2, VBP1, PSMA6, CNBP, CDK2, TARDBP, NOLC1, GSPT1, HNRNPA2B1, ETF1, RPL6, TOMM70, PTGES3, PSMA1, PSMC6, CTPS1, HSPE1, FAM120A, PPM1G, HNRNPD, SERBP1, CSTF2, PRDX4, EIF4E, ODC1, USP1, RPL22, HNRNPA3, NCBP1, RPLP0, ABCE1, EIF1AX, YWHAE, MCM7, YWHAQ, VDAC1, SMARCC1, SNRPD2, HNRNPU, HDDC2, PSMD3, RRM1, SF3A1, LSM7, ERH, RPL14, PSMB3, IFRD1, NCBP2, GLO1, NDUFAB1, CUL1, NHP2, CLNS1A, TRIM28, RSL1D1, HNRNPC, COPS5, XRCC6, SLC25A3, MRPL9, POLE3, POLD2, H2AZ1, AIMP2, NOP56, PRDX3, MRPS18B, EPRS1, KPNA2, HSP90AB1, RPL34, SRPK1, MAD2L1, DHX15, MCM4, RPS5, CCT7, HDGF, RPS6, SNRPB2, PSMC4, CDC20, TUFM, RRP9, CDC45, TYMS, ILF2, VDAC3, IMPDH2, SF3B3, NOP16, SRSF7, GNL3, EXOSC7, MRPL23, RAD23B, RPS10, PHB, DDX21, CYC1, PABPC1
**Myc Targets V2**	SLC19A1, MRTO4, TMEM97, RRP9, PES1, TFB2M, EXOSC5, IPO4, NDUFAF4, NOC4L, MYC, SRM, PA2G4, GNL3, NOLC1, WDR43, RABEPK, NOP16, TBRG4, DDX18, NIP7, WDR74, BYSL, HSPD1, PLK4, NOP2, PPAN, NOP56, RCL1, NPM1, AIMP2, RRP12, PPRC1, TCOF1, MCM5, HK2, CBX3, PLK1, PHB, MCM4, CDK4, DUSP2, MYBBP1A, UTP20, PRMT3, FARSA, MAP3K6, LAS1L, PUS1, HSPE1, SLC29A2, DCTPP1, SUPV3L1, SORD, IMP4, GRWD1, UNG, MPHOSPH10
**Epithelial Mesenchymal Transition**	COL3A1, COL5A2, COL5A1, FBN1, COL1A1, FN1, COL6A3, SERPINE1, COL1A2, COL4A1, COL4A2, VCAN, IGFBP3, TGFBI, SPARC, LUM, LAMC1, LOX, LAMC2, CCN2, TAGLN, COL7A1, LOXL2, COL6A2, ITGAV, THBS2, COL16A1, NNMT, TPM1, CDH2, MMP2, COL11A1, THBS1, FAP, BGN, SERPINH1, FSTL1, POSTN, THY1, SPP1, TNC, TFPI2, NID2, ITGB5, MMP3, VIM, LOXL1, FBLN5, COL12A1, ELN, CDH11, COMP, SPOCK1, BMP1, IL32, LAMA3, TIMP1, QSOX1, TIMP3, VCAM1, CCN1, EDIL3, CALD1, MAGEE1, FBLN1, SGCB, ECM1, LAMA2, FSTL3, TPM2, INHBA, DAB2, EMP3, BASP1, ITGA5, MGP, VEGFA, CXCL1, WNT5A, SDC1, PLOD2, PCOLCE, GREM1, ITGB1, COL5A3, RHOB, HTRA1, FGF2, SNTB1,

	GADD45A, MEST, LRRC15, TNFRSF11B, CD59, ACTA2, EFEMP2, MATN2, PCOLCE2, SERPINE2, GPC1, ABI3BP, FUCA1, SLIT3, LAMA1, PMEPA1, COL8A2, FBN2, IGFBP2, PFN2, SDC4, CD44, GADD45B, CXCL8, GLIPR1, ANPEP, P3H1, VEGFC, MMP14, SGCD, PLOD1, MATN3, MYL9, SLC6A8, CALU, PRRX1, TNFRSF12A, FMOD, ID2, GEM, PLAUR, MYLK, TGFB1, SFRP1, PLOD3, IL6, APLP1, FBLN2, MSX1, PTX3, FZD8, JUN, FERMT2, DKK1, SNAI2, DST, TPM4, DCN, GJA1, PMP22, IGFBP4, COPA, LRP1, ITGA2, FLNA, MFAP5, PTHLH, TGFBR3, SFRP4, LGALS1, RGS4, CDH6, SAT1, NT5E, DPYSL3, PPIB, TGM2, SGCG, ITGB3, PDLIM4, CTHRC1, ECM2, CRLF1, AREG, IL15, MCM7, GAS1, PRSS2, CADM1, OXTR, SCG2, CXCL6, MMP1, TNFAIP3, CAPG, CAP2, MXRA5, FOXC2, NTM, ENO2, FAS, BDNF, ADAM12, PVR, CXCL12, PDGFRB, SLIT2, NOTCH2, COLGALT1, GPX7, WIPF1
**Inflammatory Response**	CXCL10, CCL2, CCL5, FPR1, CCL20, IL1A, CXCL8, CCL7, CCL22, CXCL11, CCR7, EDN1, CD40, CXCL9, IL6, IL1B, TLR2, IL1R1, CD69, ICAM1, CCRL2, AQP9, EREG, C3AR1, GNA15, CMKLR1, PTGER4, LIF, IL15, NAMPT, OPRK1, ITGB8, PTAFR, ADM, PLAUR, NFKB1, INHBA, OSM, TNFSF10, TNFSF15, IFNGR2, ADGRE1, IL12B, CSF1, CXCL6, TNFRSF9, LYN, ACVR2A, LDLR, BDKRB1, HRH1, F3, BST2, PTGIR, CD55, CALCRL, CSF3, GPR132, IL4R, NLRP3, IL15RA, ADORA2B, GCH1, OLR1, PTGER2, CSF3R, MYC, RELA, TNFAIP6, IL7R, IL18, GABBR1, CD82, TNFSF9, NMUR1, IL2RB, TLR1, LPAR1, IRAK2, RIPK2, MMP14, P2RX7, SLC11A2, SELL, P2RY2, ABCA1, FFAR2, PROK2, GNAI3, TACR1, SLC7A1, CDKN1A, CYBB, TIMP1, HBEGF, SCARF1, EBI3, NFKBIA, SRI, SLC7A2, CCL17, TLR3, APLNR, OSMR, IL10RA, PSEN1, GPR183, ATP2B1, TNFRSF1B, BEST1, GPC3, SCN1B, ACVR1B, HPN, SEMA4D, KLF6, CD48, CXCR6, SLC1A2, GP1BA, TAPBP, RGS16, SLAMF1, LCK, HIF1A, AHR, NMI, RHOG, TPBG, NPFFR2, IFNAR1, ICOSLG, RASGRP1, IFITM1, KCNJ2, LY6E, IL18R1, IL10, KCNA3, HAS2, DCBLD2, LAMP3, VIP, CD70, RGS1, SLC31A1, ADRM1, KCNMB2, SERPINE1, MXD1, AXL, MEFV, PVR, CCL24, PDE4B, LCP2, PDPN, IRF7, MET, ATP2A2, SLC31A2, FZD5, ITGA5, SGMS2, MARCO, CD14, EIF2AK2, ROS1, ATP2C1, NDP, BTG2, MSR1, PTPRE, RNF144B, PCDH7, SPHK1, IL18RAP, RTP4, RAF1, CHST2, ITGB3, KIF1B, SELE, NOD2, C5AR1, EMP3, CLEC5A, TACR3, SLC4A4, MEP1A, SELENOS, LTA, PIK3R5, STAB1, IRF1, ICAM4, P2RX4, ABI1, CX3CL1, SLC28A2
**Xenobiotic Metabolism**	CYP1A1, FAH, DCXR, CYP1A2, GSTA3, CYP2J2, CYP27A1, ADH1C, UGDH, ASL, NQO1, ALAS1, GSTM4, CYP2C18, PTS, CYB5A, ACOX2, EPHX1, LCAT, SLC35D1, GSS, FMO3, IDH1, AKR1C3, SLC22A1, GSTO1, ARG1, CDO1, FMO1, ALDH9A1, POR, NDRG2, UPB1, ECH1, PTGES, PDK4, AHCY, COMT, AOX1, CFB, DDAH2, HGFAC, F11, CYP26A1, MCCC2, SERPINA6, GNMT, BLVRB, HACL1, MAOA, KYNU, DHPS, SERPINE1, SLC6A12, GCH1, DHRS7, PAPSS2, ALDH2, HSD11B1, SLC35B1, KARS1, CA2, LPIN2, CAT, CYP2S1, RAP1GAP, FBP1, RBP4, GCKR, CDA, MTHFD1, ADH7, DHRS1, VNN1, CNDP2, SLC6A6, FETUB, CROT, GAD1, AKR1C2, TKFC, IGF1, SSR3, GABARAPL1, TMBIM6, CD36, CES1, SHMT2, TMEM176B, GSTT2, PLG, HMOX1, SPINT2, ALDH3A1, GSR, ABHD6, XDH, PPARD, ELOVL5, CYP4F2, RETSAT, CYFIP2, PINK1, GCLC, ESR1, PMM1, TDO2, MT2A, ATP2A2, DDT, FABP1, ACP2, ABCD2, G6PC1, DDC, ACOX3, SAR1B, FBLN1, ADH5, CRP, ABCC2, ETS2, NPC1, CASP6, BPHL, IL1R1, ENTPD5, TNFRSF1A, IGFBP4, PYCR1, CSAD, VTN, ID2, ETFDH, HRG, TYR, UPP1, BCAR1, CBR1, APOE, SLC12A4, TMEM97, SLC46A3, PTGDS, MPP2, PTGR1, AQP9, EPHA2, PSMB10, FAS, ACOX1, GART, HPRT1, PGD, ABCC3, SMOX, TTPA, HES6, GCNT2, MAN1A1, IGFBP1, ITIH4, PROS1, TPST1, LEAP2, NFS1, PGRMC1, CYP17A1, ARPP19, SERTAD1, ARG2, HNF4A,

	REG1A, CCL25, PEMT, ACO2, LONP1, NMT1, PTGES3, F10, TAT, MARCHF6, JUP, ACP1, MBL2, TGFB2, ENPEP, HSD17B2, ANGPTL3, PC, PDLIM5, CYP2E1, BCAT1, ATOH8, SLC1A5, IRF8, NINJ1, AP4B1, ACSM1, ITIH1
**Fatty Acid Metabolism**	ACAA1, ACAA2, ACADL, ACADM, ACOT8, ACOX1, ACSL1, ALDH3A2, MIX23, CPT2, CYP4A11, DECR1, ECH1, ECI1, ELOVL5, FABP1, FABP2, HADH, HIBCH, HMGCL, HSD17B11, IDH1, ME1, MGLL, MLYCD, PCBD1, RETSAT, S100A10, SUCLG1, VNN1, EHHADH, ALDH9A1, HADHB, ECHS1, ACADS, CA2, HSD17B10, ALDH1A1, ACADVL, HSD17B4, CA4, ADH1C, ADH7, PTS, MAOA, HAO2, HSD17B7, MCEE, ACAT2, AUH, HPGD, FH, HMGCS2, ALAD, GPD1, ACO2, CBR1, GRHPR, ACOT2, G0S2, MDH2, HSP90AA1, BCKDHB, UROS, YWHAH, LDHA, CRYZ, RDH16, INMT, UGDH, GSTZ1, IDH3B, MDH1, CRAT, ETFDH, CD36, ECI2, SDHD, ACSL5, HSDL2, HMGCS1, SDHC, CD1D, GCDH, GPD2, ALDH3A1, SLC22A5, PDHB, TDO2, FASN, NBN, PSME1, PPARA, NCAPH2, BPHL, ODC1, CA6, DLD, HCCS, EPHX1, DLST, FMO1, AOC3, UROD, CPT1A, KMT5A, UBE2L6, MIF, SUCLG2, CPOX, SMS, CBR3, NTHL1, CIDEA, IDI1, AADAT, REEP6, HSPH1, APEX1, NSDHL, ADIPOR2, ACSL4, ACSS1, ENO3, IDH3G, LGALS1, METAP1, ALDOA, ACSM3, LTC4S, D2HGDH, ADSL, SUCLA2, SDHA, XIST, OSTC, GLUL, CYP4A22, GABARAPL1, AQP7, CYP1A1, PRDX6, ERP29, H2AZ1, GAPDHS, DHCR24, GAD2, PTPRG, IL4I1, TP53INP2, PDHA1, RAP1GDS1, CEL, BLVRA, SERINC1, BMPR1B, RDH11, ENO2
**Oxidative Phosphorylation**	NDUFS3, UQCRB, NDUFS2, SDHA, UQCRC1, NDUFA9, NDUFS4, NDUFS1, NDUFA2, NDUFS8, SDHB, NNT, ATP5PO, ATP5MC3, NDUFS7, ATP5F1A, NDUFV1, COX5B, UQCRH, NDUFA1, ATP5F1C, ATP5F1B, COX7B, SDHD, CYCS, NDUFA6, NDUFAB1, COX8A, ACO2, ATP5MC1, CYC1, NDUFB6, ATP5F1E, COX5A, UQCRC2, COX6A1, ATP5F1D, COX6C, ATP5PF, NDUFB3, IDH3B, OGDH, NDUFB8, SURF1, COX6B1, NDUFB5, NDUFA4, NDUFB1, COX4I1, COX7C, UQCRFS1, SDHC, ATP6V1F, COX7A2, SUCLG1, NDUFS6, NDUFA7, FH, NDUFV2, OXA1L, NDUFC1, UQCR11, NDUFA5, CS, ATP6V1G1, ATP5PB, HCCS, HADHB, ATP5PD, PDHA1, NDUFA8, DLD, OPA1, SLC25A11, ATP5ME, PDHB, ATP5MF, NDUFB7, IDH2, MTX2, VDAC3, MDH1, ATP5MC2, IMMT, MDH2, SLC25A3, ATP6V1D, VDAC2, ACADM, COX7A2L, TIMM17A, ATP6V1E1, NDUFA3, SLC25A6, IDH3G, ACADVL, ETFA, TIMM9, IDH3A, TIMM8B, ATP6AP1, TIMM13, UQCRQ, ABCB7, VDAC1, ATP5MG, PHB2, DECR1, SUCLA2, GOT2, DLAT, ATP6V1H, NDUFB2, FDX1, HADHA, ATP6V1C1, MAOB, NDUFB4, UQCR10, ETFDH, GPX4, PDHX, MFN2, AIFM1, ACAA2, ETFB, COX11, ECHS1, PMPCA, ATP6V0B, SLC25A5, DLST, COX15, CYB5A, ALAS1, SLC25A4, CPT1A, SLC25A20, MTRR, COX17, CYB5R3, TOMM22, ACAT1, MRPS11, ATP6V0C, PDK4, TIMM10, LDHA, ECI1, MRPL11, FXN, MRPS12, COX10, RHOT1, ACAA1, ACADSB, LDHB, MRPS30, ATP1B1, BDH2, SLC25A12, TIMM50, MRPL34, ISCA1, MRPL35, IDH1, HSPA9, MRPL15, MRPS15, TOMM70, TCIRG1, ISCU, POLR2F, NQO2, NDUFC2, MRPS22, POR, ATP6V0E1, PHYH, MPC1, GPI, AFG3L2, HSD17B10, CASP7, PRDX3, MGST3, HTRA2, BCKDHA, LRPPRC, RETSAT, ECH1, RHOT2, BAX, MTRF1, GLUD1, SUPV3L1, GRPEL1, PDP1, ALDH6A1, OAT
**Glycolysis**	PGK1, ALDOA, ENO1, TPI1, PFKP, ERO1A, ALDOB, VEGFA, MXI1, PKM, HK2, LDHA, EXT1, SLC25A10, GUSB, PFKFB1, PGAM1, PYGB, AK4, P4HA1, PMM2, FAM162A, SDC1, EGLN3, PC, B4GALT7, FBP2, IGFBP3, CHPF, B3GAT3, CHST12, HS2ST1, MPI, GNPDA1, AKR1A1, PPFIA4, B3GAT1, CHPF2, G6PD, MDH2, CHST6, PGLS, PGAM2, CHST1, GPC1, GFUS, ALG1, GFPT1, PRPS1, GOT1, MDH1,

	SLC35A3, GALK1, EGFR, ANGPTL4, CITED2, PLOD2, QSOX1, ME2, SPAG4, P4HA2, GAPDHS, ENO2, GOT2, EXT2, SLC25A13, HMMR, PDK3, CXCR4, GPC4, ECD, GNE, B4GALT2, FUT8, MIOX, VCAN, GPC3, B3GALT6, HSPA5, ME1, ADORA2B, UGP2, MIF, NANP, ZNF292, STC2, TPST1, PGM2, GYS1, TKTL1, TGFA, CHST2, PHKA2, STMN1, GALE, MET, LCT, IRS2, POLR3K, B4GALT1, EFNA3, LHX9, KDELR3, TALDO1, DPYSL4, VLDLR, CD44, AGL, SOX9, DDIT4, IDUA, CASP6, GLCE, COPB2, DSC2, HS6ST2, CDK1, PLOD1, SDC2, GMPPB, PAXIP1, NSDHL, RARS1, SLC16A3, GLRX, SRD5A3, SDC3, HDLBP, COL5A1, CLDN9, TFF3, STC1, KIF20A, GYS2, SLC37A4, LHPP, SDHC, NASP, AURKA, B3GNT3, ISG20, LDHC, ARPP19, CENPA, HOMER1, BIK, CYB5A, HAX1, COG2, IL13RA1, NOL3, CLDN3, AGRN, CLN6, TXN, PAM, CAPN5, PKP2, ABCB6, DCN, GMPPA, BPNT1, ANG, GPR87, GAL3ST1, ALDH7A1, NT5E, IDH1, PYGL, NDUFV3, NDST3, PPP2CB, PSMC4, TPBG, TGFBI, GALK2, CTH, KIF2A, CACNA1H, ANKZF1, SAP30, RBCK1, ELF3, RPE, B4GALT4, DEPDC1, RRAGD, IER3, ALDH9A1, DLD, MERTK, GCLC, FKBP4, SOD1, MED24, AK3, XYLT2, ARTN, PPIA, CHST4
**Reactive Oxygen Species Pathway**	GSR, PRDX2, TXNRD1, SOD1, GCLC, CAT, GPX4, MPO, PRDX1, PRDX6, GCLM, TXN, SOD2, PRDX4, SELENOS, PDLIM1, TXNRD2, LAMTOR5, G6PD, MSRA, MBP, SRXN1, NQO1, OXSR1, GLRX2, HMOX2, FES, PFKP, NDUFB4, GPX3, SCAF4, CDKN2D, MGST1, ABCC1, FTL, ATOX1, STK25, EGLN2, ERCC2, SBNO2, JUNB, PTPA, LSP1, NDUFA6, HHEX, GLRX, NDUFS2, PRNP, IPCEF1
**P53 Pathway**	CDKN1A, BTG2, MDM2, CCNG1, FAS, TOB1, GADD45A, PHLDA3, TAP1, CDKN2B, SFN, ZMAT3, DDB2, EI24, PERP, DDIT4, ATF3, BAX, SESN1, FDXR, PIDD1, SAT1, CDKN2A, AEN, PPM1D, FOXO3, BTG1, TXNIP, SLC19A2, IER3, TP53, NOTCH1, RRAD, DCXR, NINJ1, FOS, S100A10, FBXW7, PLK3, XPC, AK1, TRIAP1, BAK1, CCND2, APAF1, PDGFA, NDRG1, RALGDS, SERPINB5, DGKA, CYFIP2, KLF4, IP6K2, ADA, TNFSF9, KLK8, PRKAB1, STOM, SOCS1, PLK2, CASP1, RCHY1, PCNA, RPS27L, RB1, PPP1R15A, POLH, RAB40C, ERCC5, CDH13, DDIT3, TGFB1, FUCA1, H2AW, TP63, HSPA4L, EPHX1, ABHD4, H2AJ, TRAF4, CTSF, IER5, TAX1BP3, TSC22D1, RGS16, HRAS, PVT1, ZFP36L1, SDC1, WRAP73, RAP2B, COQ8A, PLXNB2, HEXIM1, PROCR, SPHK1, NOL8, DEF6, SP1, JAG2, CCP110, DRAM1, RPL18, CEBPA, KIF13B, MXD1, TRAFD1, OSGIN1, ANKRA2, HBEGF, RAD51C, ITGB4, TRIB3, INHBB, VAMP8, VWA5A, ABAT, CTSD, PTPRE, HINT1, ALOX15B, VDR, ELP1, WWP1, NUPR1, TCN2, CDKN2AIP, POM121, ZNF365, EPHA2, JUN, MAPKAPK3, SLC7A11, SERTAD3, IRAK1, ABCC5, UPP1, CD81, LIF, RPL36, FAM162A, RNF19B, CDK5R1, STEAP3, PITPNC1, SLC35D1, TPRKB, RRP8, IL1A, PMM1, TPD52L1, IFI30, GM2A, RPS12, EPS8L2, TM4SF1, BLCAP, MKNK2, CD82, RACK1, ST14, TCHH, HDAC3, CCNK, RHBDF2, NUDT15, TGFA, ISCU, LDHB, TNNI1, FGF13, RXRA, APP, F2R, CLCA2, CGRRF1, CSRNP2, ACVR1B, BMP2, GPX2, KRT17, TM7SF3, ZBTB16, PTPN14, TSPYL2, SLC3A2, S100A4, GLS2, CCND3, PRMT2, NHLH2, BAIAP2, RETSAT, IRAG2, H1-2, DNTTIP2, MXD4, SEC61A1, HMOX1, RAD9A
**Uv Response Up**	NXF1, SLC6A8, BAK1, CDC5L, HSPA2, POLR2H, ALAS1, TUBA4A, C4BPB, CCNE1, EPHX1, GRPEL1, ICAM1, FEN1, ARRB2, JUNB, CTSV, DGAT1, ATF3, CASP3, RASGRP1, TMBIM6, FOS, TAP1, E2F5, RAB27A, CCND3, SLC25A4, SPR, CDKN2B, SHOX2, OLFM1, IGFBP2, LYN, CLTB, BCL2L11, LHX2, NFKBIA, MSX1, H2AX, NR4A1, GLS, CDC34, MMP14, STK25, PDAP1, ABCB1, BSG, SELENOW, IRF1, RXRB, TFRC, PPIF, CDKN1C, HLA-F, CREG1, APOM, BMP2, TGFBRAP1, NAT1, CYB5R1, RRAD, POLE3, IL6, YKT6, ACAA1, CLCN2, AGO2, SOD2, PPP1R2, RHOB, RET, PDLIM3,

	EPCAM, DNAJA1, SULT1A1, CHKA, CYP1A1, ATP6V1F, CDO1, ALDOA, NPTXR, EIF2S3, CNP, GRINA, BID, POLG2, IL6ST, HYAL2, FOSB, MGAT1, NKX2-5, PPAT, FURIN, MRPL23, HTR7, CXCL2, KLHDC3, PRPF3, DNAJB1, AP2S1, PSMC3, TST, SIGMAR1, GCH1, BTG2, CEBPG, TARS1, UROD, PARP2, BTG3, DLG4, CHRNA5, NPTX2, ATP6V1C1, AQP3, PLCL1, NUP58, TYRO3, HMOX1, RPN1, BTG1, FMO1, ASNS, DDX21, KCNH2, COL2A1, MAOA, TCHH, CYB5B, HSPA13, GGH, GAL, NTRK3, PPT1, FGF18, FKBP4, STIP1, EIF5, ENO2, GPX3, PRKACA, PRKCD, STARD3, MARK2, SLC6A12, SQSTM1, PTPRD, MAPK8IP2, CDK2, ONECUT1, RFC4, CA2, TACR3, AMD1, HNRNPU, WIZ, CCK
**Uv Response Dn**	TJP1, NFIB, TGFBR3, TFPI, MMP16, ABCC1, MAP2K5, AGGF1, PLCB4, MET, IRS1, PTPRM, AKT3, SIPA1L1, NR1D2, ATRN, ANXA2, ADD3, CDKN1B, ATXN1, CAP2, PRDM2, TOGARAM1, SDC2, SMAD3, SLC7A1, GCNT1, RUNX1, RND3, PLPP3, NR3C1, YTHDC1, PPARG, ADGRL2, DLG1, ATP2B4, NIPBL, PEX14, MYC, RASA2, FZD2, BMPR1A, LPAR1, PRKCA, MGMT, PRKCE, RXRA, LTBP1, LDLR, RBPMS, CACNA1A, RGS4, COL5A2, PTPN21, PRKAR2B, GJA1, BCKDHB, DMAC2L, SERPINE1, AMPH, MIOS, CITED2, COL1A2, INSIG1, FYN, WDR37, ZMIZ1, PMP22, CDON, PIK3R3, NOTCH2, SYNJ2, SMAD7, MAP1B, TENT4A, PTEN, DYRK1A, NEK7, SPOP, F3, ERBB2, GRK5, DUSP1, VLDLR, CAV1, COL11A1, CDC42BPA, CDK13, SFMBT1, ADORA2B, BHLHE40, COL3A1, KCNMA1, SYNE1, IGF1R, CELF2, MTA1, EFEMP1, LAMC1, PHF3, SCAF8, VAV2, PIAS3, ACVR2A, NRP1, MRPS31, CCN1, DAB2, MAGI2, ANXA4, COL1A1, SCHIP1, DDAH1, PDGFRB, ATRX, KALRN, TGFBR2, PTGFR, ATP2C1, ARHGEF9, NFKB1, MGLL, FBLN5, PDLIM5, MAPK14, SRI, INPP4B, IGFBP5, ITGB3, BDNF, ID1, SNAI2, SCN8A, ATP2B1, FHL2, KIT, APBB2, PIK3CD, DLC1, MT1E, DBP, HAS2, SLC22A18, ICA1
**Angiogenesis**	VCAN, POSTN, FSTL1, LRPAP1, STC1, LPL, VEGFA, PF4, THBD, FGFR1, TNFRSF21, CCND2, COL5A2, ITGAV, SERPINA5, KCNJ8, APP, JAG1, COL3A1, SPP1, NRP1, OLR1, PDGFA, PTK2, SLCO2A1, PGLYRP1, VAV2, S100A4, MSX1, VTN, TIMP1, APOH, PRG2, JAG2, LUM, CXCL6
**Heme Metabolism**	ALAS2, PPOX, FECH, HMBS, GYPB, ALAD, RHD, GYPA, SELENBP1, BLVRB, GYPE, GYPC, EPB42, SLC4A1, RHCE, RHAG, TAL1, MARCHF8, SPTB, UROD, HBQ1, DCAF11, ERMAP, EIF2AK1, CPOX, MPP1, GLRX5, TSPAN5, OSBP2, ANK1, NFE2, AHSP, SPTA1, MAP2K3, BNIP3L, SLC22A4, CLIC2, TRAK2, GCLC, KEL, RANBP10, TRIM10, TSPO2, UROS, XK, SNCA, MARK3, UBAC1, CA1, CA2, BSG, FOXO3, KLF1, CDC27, KAT2B, NARF, GCLM, FBXO7, H1-0, HEBP1, HBD, NUDT4, FBXO9, RAD23A, CROCCP2, TMCC2, BLVRA, GATA1, RBM38, PRDX2, RNF123, MINPP1, TNS1, CTSE, DMTN, HDGF, XPO7, MKRN1, SMOX, TFDP2, MXI1, HBB, MOSPD1, SLC25A37, SLC30A1, ABCB6, SLC6A8, TFRC, RCL1, TOP1, EPOR, TNRC6B, ACSL6, GAPVD1, MGST3, SLC30A10, CAT, BCAM, HBZ, ACKR1, PSMD9, ISCA1, PDZK1IP1, H4C3, ICAM4, MOCOS, ADD1, FTCD, ADD2, BMP2K, BPGM, CLCN3, E2F2, HAGH, KDM7A, OPTN, RAP1GAP, CTNS, NFE2L1, ELL2, SLC2A1, PIGQ, KLF3, EPB41, LAMP2, TMEM9B, EZH1, CTSB, ATG4A, MFHAS1, MYL4, RNF19A, BTG2, SLC11A2, ADIPOR1, ASNS, DCUN1D1, ALDH1L1, DCAF10, YPEL5, SEC14L1, USP15, RIOK3, SIDT2, FOXJ2, TCEA1, VEZF1, LPIN2, PC, HBBP1, CDR2, CCND3, SLC25A38, TENT5C, FBXO34, BACH1, NR3C1, AQP3, TRIM58, LMO2, PICALM, NCOA4, GDE1, SLC6A9, NNT, ARHGEF12, PPP2R5B, C3, MARCHF2, UCP2, SYNJ1, FN3K, ABCG2, DAAM1, AGPAT4, CCDC28A, GMPS, ACP5, SLC10A3, ALDH6A1, CIR1, RBM5, HTRA2, IGSF3, BTRC, PGLS, SLC66A2,

	SDCBP, LRP10, NEK7, ARL2BP, ENDOD1, P4HA2, HTATIP2, SLC7A11, KHNYN, MBOAT2, ATP6V0A1, CAST, TYR
**Coagulation**	F2, PROC, C1S, MMP14, F10, PLG, C1R, SERPINE1, SERPING1, C2, F12, CFI, MMP2, C3, F9, CTSO, TMPRSS6, MMP9, PROZ, MMP1, CFB, BMP1, VWF, FGA, F11, THBD, FURIN, MMP11, F8, LGMN, HPN, CTSB, MBL2, F13B, TIMP1, CTSV, MMP8, A2M, MMP10, MASP2, FGG, C8A, PLAU, ADAM9, GSN, CAPN2, PLAT, FN1, CTSH, OLR1, SPARC, RGN, C8B, LAMP2, C9, PROS1, CLU, F2RL2, CD9, CFH, PREP, LTA4H, GP9, DUSP14, CTSK, GDA, CASP9, APOC1, CRIP2, KLK8, SERPINC1, ANXA1, ITIH1, PRSS23, FBN1, MST1, F3, KLF7, CTSE, ITGB3, CAPN5, CSRP1, KLKB1, MSRB2, TFPI2, MMP3, MEP1A, GP1BA, APOA1, SERPINA1, TF, PEF1, GNG12, SIRT2, COMP, HTRA1, MAFF, LRP1, PF4, CPQ, MMP15, RABIF, HRG, C8G, APOC2, SERPINB2, RAPGEF3, CPN1, ITGA2, RAC1, DPP4, WDR1, THBS1, FYN, PDGFB, SH2B2, PECAM1, MMP7, DUSP6, CPB2, S100A13, CFD, DCT, LEFTY2, ANG, C1QA, HMGCS2, ISCU, PLEK, APOC3, P2RY1, USP11, TIMP3, ACOX2, HNF4A, S100A1, GNB2, ARF4
**Il2 Stat5 Signaling**	SOCS2, CISH, PIM1, IL2RA, TNFRSF4, SOCS1, TNFRSF9, XBP1, RRAGD, HK2, PHLDA1, IL2RB, CTLA4, NFIL3, CD83, IKZF2, IL10, TNFRSF18, DHRS3, ECM1, ADAM19, SLC2A3, HIPK2, BATF3, BHLHE40, PTGER2, DENND5A, ITIH5, PHTF2, GADD45B, NRP1, NCOA3, CD79B, AHR, TNFRSF1B, NDRG1, BCL2L1, GABARAPL1, LIF, TIAM1, BMPR2, MAP3K8, RHOB, MYC, S100A1, ETFBKMT, CAPG, ST3GAL4, PENK, IRF4, CST7, WLS, TLR7, IKZF4, GBP4, RGS16, SPP1, IL13, SLC29A2, NFKBIZ, IL4R, MXD1, CSF2, FAH, CTSZ, ITGAE, MUC1, MAPKAPK2, TNFRSF21, NT5E, FLT3LG, CCND2, TRAF1, LCLAT1, IL3RA, CYFIP1, BCL2, FGL2, PRNP, EEF1AKMT1, PUS1, ITGAV, NCS1, DCPS, AMACR, FAM126B, PTH1R, ODC1, IGF1R, PTCH1, ENO3, CD81, MAFF, EMP1, CDKN1C, CAPN3, IL1R2, SYT11, TTC39B, ANXA4, BATF, P4HA1, GPR65, SLC1A5, IGF2R, CKAP4, CCR4, CD44, P2RX4, GATA1, KLF6, ARL4A, HOPX, GPR83, ITGA6, CD48, DRC1, SELP, GLIPR2, SMPDL3A, PLSCR1, FURIN, SERPINB6, TNFSF11, GPX4, LRRC8C, CCNE1, CASP3, SH3BGRL2, SNX9, PLEC, BMP2, ICOS, ALCAM, LTB, ENPP1, IL1RL1, MYO1C, IFNGR1, PLIN2, IL18R1, AHNAK, PRKCH, TNFRSF8, SYNGR2, GALM, POU2F1, EOMES, NOP2, PTRH2, RHOH, CDC6, MYO1E, CXCL10, SNX14, IRF6, IL10RA, MAP6, TNFSF10, SPRED2, SELL, SERPINC1, CDCP1, RORA, COCH, CSF1, F2RL2, UCK2, CA2, IFITM3, UMPS, HUWE1, COL6A1, ABCB1, RNH1, IRF8, GUCY1B1, AHCY, PRAF2, GSTO1, TWSG1, CDC42SE2, PLAGL1, APLP1, PLPP1, SPRY4, SCN9A, SHE, PDCD2L, CCND3, LRIG1, SWAP70, SLC39A8, RABGAP1L, TGM2, PNP, AGER, ETV4, CD86
**Bile Acid Metabolism**	SCP2, ABCD3, SLC27A2, HSD3B7, HSD17B4, AKR1D1, ABCD2, PEX1, CYP8B1, PNPLA8, PHYH, SLC27A5, CYP7B1, CYP27A1, PEX26, AGXT, PEX7, CROT, CYP39A1, ABCA3, CYP7A1, HACL1, PIPOX, PEX6, SOD1, GNPAT, HSD3B1, ABCA2, HAO1, PEX16, ABCD1, ACSL1, MLYCD, ABCG8, AQP9, ISOC1, NR1H4, PEX19, NPC1, HSD17B11, AMACR, CYP46A1, PEX13, IDH1, PEX11A, HSD17B6, EPHX2, PRDX5, PXMP2, BBOX1, FADS2, ALDH9A1, APOA1, OPTN, GSTK1, ABCG4, ALDH8A1, NR3C2, ABCA6, RETSAT, FADS1, PECR, PEX12, CAT, GNMT, ABCA9, ACSL5, LONP2, DHCR24, ABCA8, PFKM, DIO1, RXRA, FDXR, TTR, KLF1, SLC29A1, BCAR3, LCK, PEX11G, SLCO1A2, ALDH1A1, SULT1B1, NR1I2, GC, ABCA4, SLC35B2, SLC23A1, NUDT12, ABCA1, PAOX, SLC22A18, DIO2, RBP1, SLC23A2, ATXN1, GCLM, NEDD4, RXRG, AR, BMP6, SERPINA6, LIPE, EFHC1, IDH2, IDI1, CH25H, ABCA5, SULT2B1, SOAT2, TFCP2L1, NR0B2
**Peroxisome**	ABCD3, ACOT8, ACOX1, ACSL1, ECH1, ECI2, EHHADH, GSTK1,

	HSD17B4, MLYCD, PEX11A, RETSAT, SLC27A2, PEX13, PEX14, SCP2, HSD3B7, GNPAT, ABCD2, SLC25A17, PEX2, ACAA1, HAO2, HSD17B11, CRAT, PEX11B, LONP2, IDH1, FIS1, PEX6, ABCB4, SOD1, ABCB1, ISOC1, YWHAH, EPHX2, ABCD1, HMGCL, ACSL5, ALDH9A1, DHCR24, ELOVL5, NUDT19, PRDX5, CTPS1, IDE, SLC23A2, PEX5, BCL10, NR1I2, TSPO, CNBP, MSH2, DHRS3, DIO1, SLC25A4, PRDX1, IDI1, HRAS, MVP, ABCC8, CLN6, CAT, ACSL4, IDH2, ABCC5, SOD2, SLC35B2, FDPS, ALB, FADS1, STS, SMARCC1, ITGB1BP1, SIAH1, SLC25A19, CDK7, RXRG, ALDH1A1, UGT2B17, CADM1, SERPINA6, CLN8, RDH11, CTBP1, HSD11B2, TTR, ERCC3, ATXN1, SULT2B1, CRABP2, CRABP1, TOP2A, SCGB1A1, ERCC1, DLG4, PABPC1, FABP6, ABCB9, CACNA1B, SEMA3C, VPS4B, CEL, ESR2
**Allograft Rejection**	PTPRC, IL12B, TGFB1, IL12A, CD3E, CD3D, CD28, LYN, HCLS1, IL18, CRTAM, IFNG, CD3G, CD86, IL10, UBE2N, BCL10, CD4, LCK, NCK1, C2, HLA-A, ITGB2, HLA-DQA1, CD1D, CD80, HLA-DRA, THY1, TLR1, HLA-G, HLA-DMB, IL7, IL4, TNF, CD247, IL2, HLA-DMA, STAT1, IRF4, SRGN, INHBA, TLR3, ZAP70, CD74, CD40, TRAF2, B2M, BCL3, LTB, IFNGR1, CCR5, CD40LG, HLA-DOA, GLMN, IL6, HLA-E, CD2, CCL5, FAS, FASLG, TLR6, PF4, TGFB2, CD79A, INHBB, ELANE, SPI1, MAP3K7, IL15, CTSS, CD47, PRF1, IL12RB1, LCP2, SOCS1, CDKN2A, STAT4, CD7, HLA-DOB, CD8A, ICAM1, CCL4, GZMB, CSF1, IL11, STAB1, IL2RA, NLRP3, CCND3, EIF3A, SIT1, IFNAR2, HDAC9, CARTPT, TRAT1, CCL22, APBB1, FYB1, IL1B, TIMP1, RPS19, JAK2, KRT1, WARS1, IFNGR2, CCR2, EREG, MMP9, EGFR, IL16, CFP, WAS, ITGAL, KLRD1, RARS1, TLR2, CCND2, IL2RG, ETS1, ITK, NCR1, MAP4K1, CCL19, PSMB10, RPL39, EIF3J, ABCE1, CD8B, F2, ELF4, LY86, FCGR2B, GBP2, PRKCG, RPS9, MTIF2, GZMA, AARS1, CD96, CSK, HIF1A, CCL2, ICOSLG, NPM1, IL4R, CCL11, NME1, FLNA, GPR65, ACHE, EIF3D, IGSF6, F2R, IL13, TAP1, DARS1, IRF7, ACVR2A, CXCR3, PRKCB, CXCL9, PTPN6, NCF4, UBE2D1, LIF, CCR1, MBL2, DEGS1, TPD52, AKT1, RIPK2, IKBKB, GCNT1, SOCS5, IRF8, TAP2, EIF4G3, ABI1, CCL7, IL2RB, BRCA1, FGR, IL18RAP, MRPL3, CXCL13, CAPG, EIF5A, RPS3A, GALNT1, ST8SIA4, CCL13, RPL3L, LY75, TAPBP, NOS2, RPL9, BCAT1, IL9, IL27RA, DYRK3
**Spermatogenesis**	PDHA2, TSSK2, TNP1, ZPBP, DPEP3, ADAM2, ACTL7B, GAPDHS, TUBA3C, LDHC, DDX25, PRM2, TCP11, PIAS2, PAPOLB, OAZ3, PHF7, ODF1, ACRBP, YBX2, CSNK2A2, NAA11, TULP2, ADAD1, SYCP1, DDX4, MLF1, TEKT2, SPATA6, PGK2, HSPA2, ACRV1, CLPB, CRISP2, ZC2HC1C, AKAP4, CCNA1, CCT6B, PACRG, GSG1, THEG, HSPA4L, CST8, DCC, HSPA1L, GSTM3, DNAJB8, IL13RA2, CAMK4, LPIN1, ZC3H14, COIL, PHKG2, KIF2C, RPL39L, SLC2A5, PCSK4, AURKA, MLLT10, NOS1, ART3, CCNB2, NPHP1, CLGN, MAST2, MTOR, CDKN3, ZNRF4, AGFG1, SNAP91, PSMG1, CHFR, TNP2, EZH2, HBZ, ARL4A, SLC12A2, BRAF, VDAC3, POMC, IFT88, TKTL1, NEK2, STAM2, H1-6, GMCL1, MTNR1A, RFC4, DBF4, PRKAR2A, TTK, DMC1, NCAPH, PEBP1, CHRM4, TOPBP1, SCG5, NEFH, MAP7, BUB1, SEPTIN4, PGS1, NPY5R, TSN, CDK1, SHE, PARP2, TNNI3, PCSK1N, IDE, GPR182, RAD17, TLE4, ACE, SIRT1, HTR5A, TALDO1, NF2, CLVS1, CNIH2, GRM8, STRBP, ADCYAP1, ALOX15, MEP1B, SCG3, GFI1, ELOVL3, IL12RB2, DMRT1, GAD1, CFTR, JAM3, IP6K1, HOXB1
**Kras Signaling Up**	ANGPTL4, ITGA2, SPRY2, HBEGF, RBP4, HSD11B1, ETV4, GLRX, DUSP6, SCG5, ETV5, ITGB2, AKT2, PPBP, G0S2, GABRA3, IRF8, BIRC3, FGF9, DCBLD2, INHBA, TFPI, TSPAN1, ADAM8, SLPI, PRKG2, MMP11, MMP10, TMEM158, TNFAIP3, PRDM1, GALNT3, ETS1, MMP9, WNT7A, IGFBP3, SPP1, ETV1, CLEC4A, CCND2,

	TSPAN7, ITGBL1, EMP1, CDADC1, KIF5C, TRIB2, SDCCAG8, PCP4, CFHR2, ALDH1A2, NR0B2, ALDH1A3, AMMECR1, SATB1, GUCY1A1, CSF2, APOD, TOR1AIP2, CMKLR1, TMEM176B, ADGRA2, LAPTM5, CD37, CAB39L, CIDEA, ZNF639, IL1B, GYPC, LY96, FLT4, SPON1, BMP2, PLEK2, IGF2, NR1H4, SNAP25, ACE, PRRX1, C3AR1, TRAF1, TLR8, ID2, TMEM100, PLAUR, GADD45G, CBX8, SCN1B, PTBP2, NAP1L2, AKAP12, PLAT, SCG3, ANO1, IL1RL2, CXCL10, ATG10, YRDC, HDAC9, PEG3, SEMA3B, TNNT2, LIF, CFB, BTC, PPP1R15A, PTPRR, CCL20, ARG1, RETN, KLF4, MMD, PDCD1LG2, H2BC3, HOXD11, TRIB1, F2RL1, ANXA10, TSPAN13, MTMR10, CFH, LAT2, ERO1A, RELN, KCNN4, TMEM176A, MAP4K1, PTGS2, IL33, MAFB, LCP1, NGF, CA2, SERPINA3, RGS16, CTSS, USP12, CPE, SPARCL1, ABCB1, USH1C, CSF2RA, BTBD3, IL2RG, DNMBP, IL10RA, EREG, PRELID3B, EPHB2, FBXO4, CROT, MPZL2, ANKH, CBR4, DOCK2, GPRC5B, RABGAP1L, MALL, STRN, ST6GAL1, PIGR, VWA5A, PSMB8, F13A1, NRP1, SOX9, JUP, ADGRL4, ZNF277, EPB41L3, PCSK1N, FUCA1, PLVAP, ADAM17, AVL9, ADAMDEC1, HKDC1, MAP7, IL7R, RBM4, BPGM, ENG, GFPT2, PLAU, GNG11, PTCD2, MAP3K1, CBL, CXCR4, NIN, IKZF1, WDR33, MYCN, FCER1G, PECAM1, CCSER2, SNAP91, EVI5, TNFRSF1B, GPNMB, TPH1
**Kras Signaling Dn**	CDH16, SPTBN2, FGFR3, NOS1, PDE6B, SIDT1, NTF3, SCN10A, TAS2R4, DTNB, HTR1D, MAST3, SLC6A3, CLDN8, TGM1, PCDHB1, THRB, YPEL1, RYR2, GDNF, CAMK1D, AMBN, LFNG, SERPINA10, ALOX12B, CALCB, FGGY, SPRR3, ATP6V1B1, EDN1, UPK3B, GAMT, PRODH, RYR1, GPR19, SLC29A3, EDN2, GPRC5C, C5, ZC2HC1C, EDAR, KCNMB1, TENT5C, STAG3, KRT4, SLC25A23, INSL5, GP1BA, KMT2D, ABCG4, MYH7, BRDT, PTGFR, KCNN1, SELENOP, IFI44L, PROP1, LGALS7, BMPR1B, PDK2, DCC, SNCB, COL2A1, UGT2B17, NPY4R, DLK2, WNT16, SLC6A14, ITGB1BP2, SLC12A3, YBX2, CKM, CPB1, MAGIX, ARHGDIG, CALML5, KRT1, MTHFR, ABCB11, MYOT, CELSR2, KLK7, THNSL2, CHRNG, NR4A2, CD40LG, KRT13, GP2, CD207, CCR8, ZBTB16, PRKN, CPA2, MEFV, CCNA1, SLC38A3, KLK8, CYP39A1, KCNQ2, SLC30A3, CHST2, ITIH3, EGF, MSH5, TEX15, CLPS, ENTPD7, CLSTN3, CYP11B2, CLDN16, HSD11B2, COQ8A, HNF1A, FGF22, TFCP2L1, OXT, KCND1, MACROH2A2, NRIP2, RGS11, CPEB3, TG, PTPRJ, NUDT11, FSHB, IDUA, TNNI3, GTF3C5, TFF2, SSTR4, COPZ2, PDCD1, SLC16A7, KCNE2, MFSD6, KLHDC8A, CNTFR, IL5, NPHS1, SCGB1A1, CCDC106, PAX3, VPREB1, TSHB, CACNA1F, AKR1B10, P2RX6, KRT15, PAX4, BTG2, GPR3, GRID2, TCL1A, PLAG1, PKP1, IRS4, IL12B, EPHA5, SOX10, SNN, CACNG1, SPHK2, IGFBP2, CAPN9, TCF7L1, TGFB2, SMPX, LYPD3, PNMT, SYNPO, MX1, IFNG, NR6A1, ACTC1, RSAD2, ADRA2C, BARD1, HTR1B, FGF16, TENM2, CDKAL1, SHOX2, SGK1, RIBC2, SKIL, NGB, ASB7, MYO15A, SLC5A5, KRT5, ZNF112, TFAP2B, VPS50, CD80, ATP4A, ARPP21, SERPINB2, TLX1, EFHD1, P2RY4
**T-cell Functional Gene Sets**	
**Naïve**	IL7R, CCR7, SELL, FOXO1, KLF2, KLF3, LEF1, TCF7, ACTN1, FOXP1
**Activation:Effector function**	FAS, FASLG, CD44, CD69, CD38, NKG7, KLRB1, KLRD1, KLRF1, KLRG1, KLRK1, FCGR3A, CX3CR1, CD300A, FGFBP2, ID2, ID3, PRDM1, RUNX3, TBX21, ZEB2, BATF, IRF4, NR4A1, NR4A2, NR4A3, PBX3, ZNF683, HOPX, FOS, FOSB, JUN, JUNB, JUND, STAT1, STAT2, STAT5A, STAT6, STAT4, EOMES
**Exhaustion**	PDCD1, LAYN, HAVCR2, LAG3, CD244, CTLA4, LILRB1, TIGIT, TOX, VSIR, BTLA, ENTPD1, CD160, LAIR1

**TCR Signaling**	CALM1, CALM2, CALM3, CAST, CD247, CD3D, CD3E, CD3G, CSK, DOK1, DOK2, FYN, LCK, NFATC2, NFATC1, NFATC4, NFATC3, PLEK, PAG1, PTPN11, PTPN2, PTPN22, PTPN4, PTPN6, PTPN7, PTPRC, PTPRCAP, S100A10, S100A11, S100A13, S100A4, S100A6, ZAP70, DUSP1, DUSP2, DUSP4, DUSP5, DUSP10, LAT, PLCG1, PLCG2, PPP3CA, PPP3CC, FOS, FOSB, FOSL1, FOSL2, JUN, JUNB, JUND, NR4A1, NR4A2, NR4A3, BATF, IRF4, SH2D2A
**Cytotoxicity**	GZMA, GZMB, GZMH, GZMK, GZMH, GNLY, PRF1, IFNG, TNF, SERPINB1, SERPINB6, SERPINB9, CTSA, CTSB, CTSC, CTSD, CTSW, CST3, CST7, CSTB, LAMP1, LAMP3, CAPN2
**Cytokine/Cytokine receptor**	CSF1, IL10RA, IL16, IL17RA, IL18RAP, IL21R, IL2RB, IL2RG, IL32, IL9R, ADAM10, ADAM8, METRNL, CD70
**Chemokine/Chemokine receptor**	CCR4, CCR5, CCR7, CXCR3, CXCR4, CXCR5, CXCR6, CCL3, CCL4, CCL4L1, CCL4L2, CCL5, CXCL13, CXCL8, XCL1, XCL2
**Senescence**	KLRK1, IFNA1, IFNAR1, TAB1, SESN2
**Anergy**	NT5E, IZUMO1R, LAG3, NRP1, DGKA, CBLB, RNF128, ITCH, NFATC2, EGR2, EGR3, NR4A1, TOB1
**NFKB Signaling**	NFKB1, NFKB2, NFKBIA, NFKBIB, NFKBIZ, CHUK, IKBKB, IKBKG, REL, RELA, RELB
**Stress response**	HIKESHI, P4HB, UBA52, MAPK1, RPA2, RPA3, TNRC6A, TNRC6B, TNRC6C, NUP210, RBX1, UBB, UBC, CREBBP, ETS1, LAMTOR1, EED, LAMTOR2, HSP90AA1, RBBP4, LAMTOR4, LAMTOR5, PSMB10, CDKN1A, ANAPC11, PSMA1, ATG4D, PSMA2, WIPI2, PSMA3, TFDP2, PSMA4, ATM, CYCS, PSMA5, WDR45, ANAPC16, PSMA6, EHMT1, PSMA7, EHMT2, TERF1, PSMC1, ATR, PSMC3, SOD1, PSMC4, PSMC5, PSMC6, PSME1, ATG3, NCF1, PSME2, ATG5, CHMP2A, CHMP2B, GSK3B, RPS19BP1, TPR, MDM4, ELOB, CDC26, ELOC, ST13, RPS6KA1, EEF1A1, RPS6KA3, CHMP4A, DNAJB1, CAMK2G, RPS27A, TXN2, PRDX1, PRDX2, DNAJB6, FKBP4, PRDX3, MAP2K3, MTMR14, ATG12, PRDX5, RHEB, PRDX6, GSTP1, ATG14, RELA, GPX1, VHL, HSPA1A, UBE2D1, TERF2IP, HSPA1B, UBE2D2, TXN, UBE2D3, HSPH1, CYBA, CDK4, HSF1, CDK6, MAPKAPK3, CITED2, MAP1LC3B, PSMD12, HIF1A, HMGA1, PSMD13, MINK1, PSMD14, HSP90AB1, PTGES3, CXCL8, HSBP1, NFKB1, UVRAG, CAT, PSMB1, CDKN2C, PSMB2, CDKN2D, FOS, PSMB3, CCS, JUN, GABARAPL1, GABARAPL2, PSMB6, KDM6B, PSMB8, PSMD2, TNIK, PSMB9, PSMD3, RANBP2, ATOX1, SEM1, PSMD6, GABARAP, PSMD7, PSMF1, CHMP3, STAT3, PSMD8, TINF2, PSMD9, CHMP7, CCAR2, HSPA4, YWHAE, BAG1, HSPA5, HSPA6, BAG3, DYNLL1, HSPA8, RB1CC1, BAG5, ANAPC5, DYNLL2, VCP, DNAJC2, CABIN1, CEBPB
**MAPK Signaling**	MAP2K1, MAP2K2, MAP2K3, MAP3K4, MAP3K5, MAP3K8, MAP4K1, MAP4K5, MAPK1, MAPK3, MAPK11, MAPK13, MAPK14, MAPK1IP1L, MAPKAPK3, MAPK8, MAPK9, MAP2K7, MAPK10, MAP3K7
**Adhesion**	ITGA1, ITGA4, ITGAE, ITGAL, ITGAM, ITGB1, ITGB2, ITGB7, SELL, SELPLG, S1PR1, VCAM1, ICAM2, ICAM3
**IFN Response**	IFIT1, IFIT2, IFIT3, IFIT5, STAT1, STAT2, MX1, IRF1, IRF4, IRF7, IRF8, IRF9, ISG15, ISG20, IFITM1, IFITM2, IFITM3, OAS1, OAS2, OAS3, JAK1, JAK2, SOCS1, SOCS3, TRIM14, TRIM21, TRIM22, APOL1, APOL2, APOL6, IFNGR1, GBP1, GBP2, GBP4, GBP5, GBP3, BST2, CMPK2, DDX58, DDX60, DDX60L, IFI30, IFI35,

	IFI44, IFI44L, IFI6, IFIH1, PARP10, PARP12, PARP14
**Oxidative phosphorylation**	ATP1B3, ATP2A3, ATP2B1, ATP2B4, ATP5A1, ATP5B, ATP5C1, ATP5D, ATP5E, ATP5EP2, ATP5F1, ATP5F1A, ATP5F1B, ATP5F1C, ATP5F1D, ATP5F1E, ATP5G2, ATP5G3, ATP5H, ATP5I, ATP5IF1, ATP5J, ATP5J2, ATP5L, ATP5MC1, ATP5MC2, ATP5MC3, ATP5MD, ATP5ME, ATP5MF, ATP5MG, ATP5MPL, ATP5O, ATP5PB, ATP5PD, ATP5PF, ATP6AP1, ATP6AP2, ATP6V0B, ATP6V0C, ATP6V0D1, ATP6V0E1, ATP6V0E2, ATP6V1D, ATP6V1F, ATP6V1G1, ATP8B2, ATP8B4, COX16, COX17, COX4I1, COX5A, COX5B, COX6A1, COX6B1, COX6C, COX7A2, COX7B, COX7C, COX8A, CYC1, MT.ATP6, MT.ATP8, MT.CO1, MT.CO2, MT.CO3, MT.CYB, MT.ND1, MT.ND2, MT.ND3, MT.ND4, MT.ND4L, MT.ND5, MT.ND6, NDUFA1, NDUFA10, NDUFA11, NDUFA12, NDUFA13, NDUFA2, NDUFA3, NDUFA4, NDUFA5, NDUFA6, NDUFAB1, NDUFAF3, NDUFAF4, NDUFAF8, NDUFB10, NDUFB11, NDUFB2, NDUFB3, NDUFB4, NDUFB6, NDUFB7, NDUFB8, NDUFB9, NDUFC1, NDUFC2, NDUFS2, NDUFS3, NDUFS5, NDUFS6, NDUFS7, NDUFS8, NDUFV1, NDUFV2, SDHA, SDHB, TCIRG1, UQCRB, UQCRC1, UQCRC2, UQCRFS1, UQCRH, UQCRQ, SURF1, SURF4
**Glycolysis**	SLC2A3, SLC2A8, PFKFB3, ALDOA, ENO1, GAPDH, GPI, PGAM1, PGK1, PKM, TPI1, GYG1, MDH1, MDH2, LDHA, LDHB, ADH5, IDH2
**Fatty acid metabolism**	ACAA1, ACADM, ACADVL, ACSL5, ALDH16A1, ALDH9A1, ECH1, ECHS1, HADHA, HSD17B10, SLC27A2, DECR1, PEX16, SCP2
**Pro-apoptosis**	BCL2L11, BID, BIN1, BIN2, BAX, BAK1, CASP8, CASP3
**Anti-apoptosis**	BCL11B, BCL2L1, BAG3, BAG4, BIRC3, GADD45B
**T Cell Exhaustion Geneset**	
**T Cell Exhaustion**	PDCD1, LAYN, HAVCR2, LAG3, CD244, CTLA4, LILRB1, TIGIT, TOX, VSIR, BTLA, ENTPD1, CD160, LAIR1

Table S6. Top-ranked genes of different gene expression programs identified by cNMF.

GEP1	GEP2	GEP3	GEP4	GEP5	GEP6	GEP7	GEP8	GEP9	GEP10
SNCG	TPT1	SLC35F1	EPHA6	ADH7	APOO	S100A8	C20orf85	CEACAM6	MAL
HLA-DRB5	SNHG5	L3MBTL4	MT-ND3	CD9	UBD	S100A9	WDR38	UPK1B	SPNS2
RPL8	RPL37	IRAK3	LINC02315	PERP	MT-ATP8	IL36G	TMEM190	HOPX	C6orf15
TPI1	RPL38	TTTY14	LOC107986400	KRT8	RPL7A	PI3	ENSG00000263450	CEACAM5	PRSS27
GAPDH	OOEP	SGCZ	CCSER1	AKR1C1	RPLP0	S100A12	SNTN	TMPRSS11B	KRT78
H4C3	PDE3B	NOL4	MT-ATP6	AKR1C3	TNF	MMP1	ECRG4	SPRR3	NCCRP1
CHCHD2	RPL39	PIP5K1B	GABRG3	KRT19	PCBP2	SERPINB4	MORN5	A2ML1	SPRR1B
RAC1	CHST11	PLCB4	MT-ND4	PRDX1	MT-ND4L	MMP10	ABCC6P2	ATP12A	NDRG2
COX4I1	FYN	SOX14	MT-ND2	GSTM3	CD74	S100A7	C22orf15	LINC01559	C15orf62
NLRP1	RPLP2	KCNMA1	PCDH7	AKR1C2	RHOB	C1orf68	C7orf57	GPRC5D	VSIG2
RPS26	RPL37A	LINC01210	LINC02506	ENSG00000227088	ZFP36L2	LAMC2	ZMYND10	LIPH	SPRR2A
MMP7	RPS24	PARD3B	RERE	NTS	ALDOA	S100A7A	SRGAP3-AS2	SPINK5	SPINK7
RPS2	RPS17	CACNB4	MT-CO3	MAGEA4	ENSG00000257322	S100A2	CLXN	ENSG00000268628	TGM7
TDRD12	STEAP1B	PMAIP1	PTGS2	NHERF1	EGR1	INHBA	SRD5A2	LMO7	PSORS1C2

GPNMB	RPL36A	PRTG	WDR49	EPIC1	EEF1A1	PLAUR	ARMC3	ENSG00000291282	FAM25A
RPL13	PTPRC	RNF145	RBFOX3	GNGT1	PPP1R15A	PLAU	C9orf24	UTRN	H2AJ
LINC02945	TENM4	SINHCAF	LINC02345	AKR1B10	HLA-DQA1	SERPINB1	STMND1	SCEL	RNF39
OAZ1	TMSB4X	KYNU	TMEM108	NKAIN2	BRD2	KRT6B	CFAP73	MALL	C15orf48
ARPC1B	FBXL7	NIBAN1	IFI27	RNF217-AS1	TNFAIP3	IL36RN	CAPS	EMP1	PSCA
RPS27A	RPS29	ARL15	MT-CYB	ZFPM2-AS1	PNRC1	SOD2	AKAP14	H2AJ	KRT80
PTMA	RPS11	IL15	NEAT1	LOC105378798	ZFP36	IL1RN	ANKRD66	ENSG00000287339	VGLL1
HSPB1	ARHGAP15	LINC02384	PPM1L	AQP3	PLA1A	SERPINB3	FAM216B	BCAT1	CST6
NDUFA4	NKG7	MEGF10	ANXA1	BRDT	ATF3	HEPHL1	HOATZ	FAM3D	GABARAPL1
BTF3	THEMIS	RPS4Y1	LINC01876	CCDC190	BOLA2	PLA2G2F	C6orf118	PRSS27	S100P
HLA-DQB1	WIPF1	RNF152	SEMA6D	WIF1	NPM1	SERPINB7	TPPP3	PLAC8	TP53INP2
RPL41	SDK1	SET	CASC15	LINC02994	HSPA8	DEFB4A	ADGB	TGM6	C1orf116
RPS6	CD44	TYMP	PUM1	IMPA2	CDKN1A	S100A6	FAM166B	TMPRSS11E	AVPI1
ATP5MC3	PPP1R16B	TXNL4A	FAF1	MGST1	C11orf98	DEFB4B	DYNLRB2	VGLL3	LGALS3
COX5B	RPL26	UBE2E2	CACNA2D1	ENSG000002580	ACTG1	RNASE7	FOXN3-AS3	LINC02289	ANXA9

				84					
KRT5	DISC1	CCND2	NAALADL2-AS2	MAGEA3	ZFP36L1	S100A10	CCL15	IRAK4	SPRR2D
S100A14	RPL28	ISOC1	KCNB2	MAGEA1	DNAJC12	S100A11	CAPSL	TRIML1	H1-0
ATP5F1B	SKAP1	CHI3L1	FGF7P3	GTSF1	NACA	ANXA1	LRRC46	SCNN1A	GPRC5A
PARVB	ARID1B	SIL1	KREMEN2	CD177	CLDN4	IFITM3	DYNLT5	FETUB	CSF2RB
LDHA	TOX	RPL15	SELENOM	SOX15	RPL18	SAA1	ENSG00000282199	PRSS22	IL36RN
PA2G4	RPL21	NLGN4Y	SMYD3	ALDH3A1	CXCL3	TJP2	DNAI1	NDRG2	KRT23
LGALS1	LDLRAD4	TM7SF3	LERFS	GSTA1	TUBB4B	ENSG00000291250	LOC107985579	C19orf33	GCHFR
ENO1	CD52	SRSF9	ST6GAL1	MAGEA6	RPL6	IFITM1	CFAP221	STN1	SLC25A23
GSTP1	CD3D	ROBO1	MLF1	LY6K	ZNF296	APOBEC3B	MS4A8	RHOU	SPRR3
HLA-A	CD2	PLTP	HMGA2	YWHAZ	EEF2	KRT17	CCN2	CLDN7	LOC102724482
PKM	RPS13	NDUFV2	EFCAB2	PITX1	EIF1	ADAMDEC1	LOC124906839	S100A10	EMP1
EEF1D	CD96	KIAA1549L	SERPINE2	PTHLH	UBC	HBEGF	TUBA4B	ENSG00000257925	TIMP2
ALDH2	FMO3	BCL2	NOVA1	MAGEA12	SNHG1	LUCAT1	CCDC78	S100P	RNF222
EEF1G	LOC729732	SMARCC1	MT-CO1	FABP5	SORD	BMAL2	PIFO	KRT6A	PRSS22

TUBA1B	MYO3B	DNM3	AKT3	S100A6	OCA2	KRT6A	HYDIN	LINC00520	LINC01405
ATP5MF	SHISA6	RBBP8	MT-CO2	LINC02428	JUNB	PHLDA2	STRIT1	C1orf116	KIFC3
UBB	RPL23	MEGF11	SDCCAG8	KRT5	SNHG12	SLPI	CFAP90	QKILA	GDA
RPS20	EPSTI1	PCAT1	BAZ1A	LYPD3	DNAJA1	FTH1	RSPH9	BCAS1	EPS8L1
STAR	GUSBP5	AKT3	CFH	TPD52L1	FOS	LCE3D	RGS22	TMEM154	SERPINB7
POU6F2	IKZF3	UBE2E1	MCL1	SLCO1B3	RPS10	SFN	TSPAN1	ENSG00000289850	CCDST
PPP1R14B	ENSG00000259995	ATP1B1	MET	TACSTD2	NFKBIA	CSTB	CNGA3	CLIC5	LMO7

Abbreviations: GEP, gene expression program.
